# Editorial Note: Undergoing lignin-coated seeds to cold plasma to enhance the growth of wheat seedlings and obtain future outcome under stressed ecosystems

**DOI:** 10.1371/journal.pone.0334274

**Published:** 2025-10-10

**Authors:** The PLOS One Editors

After this article [1] was published, concerns were raised about the reproducibility of the NMR methods and results. Specifically, that insufficient detail is provided on NMR sample preparation, and that there are no replicate samples.

The corresponding author stated that solid-state NMR samples were prepared by first freezing several seeds using liquid nitrogen. They stated that the frozen seeds were then ground into a fine powder using a mortar and pestle and the resulting powder was packed into a 4 mm zirconia (ZrO₂) rotor for NMR analysis. The corresponding author also stated that the process is non-invasive and straightforward, and that the reproducibility of solid-state NMR measurements is expected to be high.

A member of the *PLOS One* Editorial Board advised that without a protocol that ensures reproducibility by preparation of multiple replicates from each treatment group, the conclusions from the interpretation of the NMR spectra are not supported, as it is not known if differences in the NMR spectra should be attributed to different treatments or to variations in the sample preparation.

It was also raised that the name of the NMR spectrometer listed in [1] is incorrect. The corresponding author stated that all NMR spectra in [1] were recorded using a Bruker 400 MHz AVANCE III NMR spectrometer.

During editorial follow-up, the corresponding author provided the original data underlying [1] to bring the article into compliance with PLOS’ Data Availability policy (S1–S7 Files).

The *PLOS One* Editors issue this Editorial Note to inform readers of the above limitations to be considered in the interpretation of the NMR results, and to provide the underlying data provided in post-publication discussions.

## Supporting information

S1 File Underlying data supporting the NMR results in [1]. (ZIP)

S2 File Underlying data supporting Fig 2. (ZIP)

S3 File Underlying data supporting Table 1 and Fig 3. (ZIP)

S4 File Underlying data supporting Table 1 and Fig 3. (ZIP)

S5 File Underlying data supporting Table 2. (ZIP)

S6 File Underlying data supporting the XPS results in [1]. (OPJ)

S7 File Underlying data supporting the XPS results in [1]. (OPJ)
