# Supplementary figures and images for: Editorial Note: Undergoing lignin-coated seeds to cold plasma to enhance the growth of wheat seedlings and obtain future outcome under stressed ecosystems
Source: PLoS One. 2025 Oct 10;20(10):e0334274. doi: 10.1371/journal.pone.0334274 (PMC12513580; doi:10.1371/journal.pone.0334274)

## Slide 1
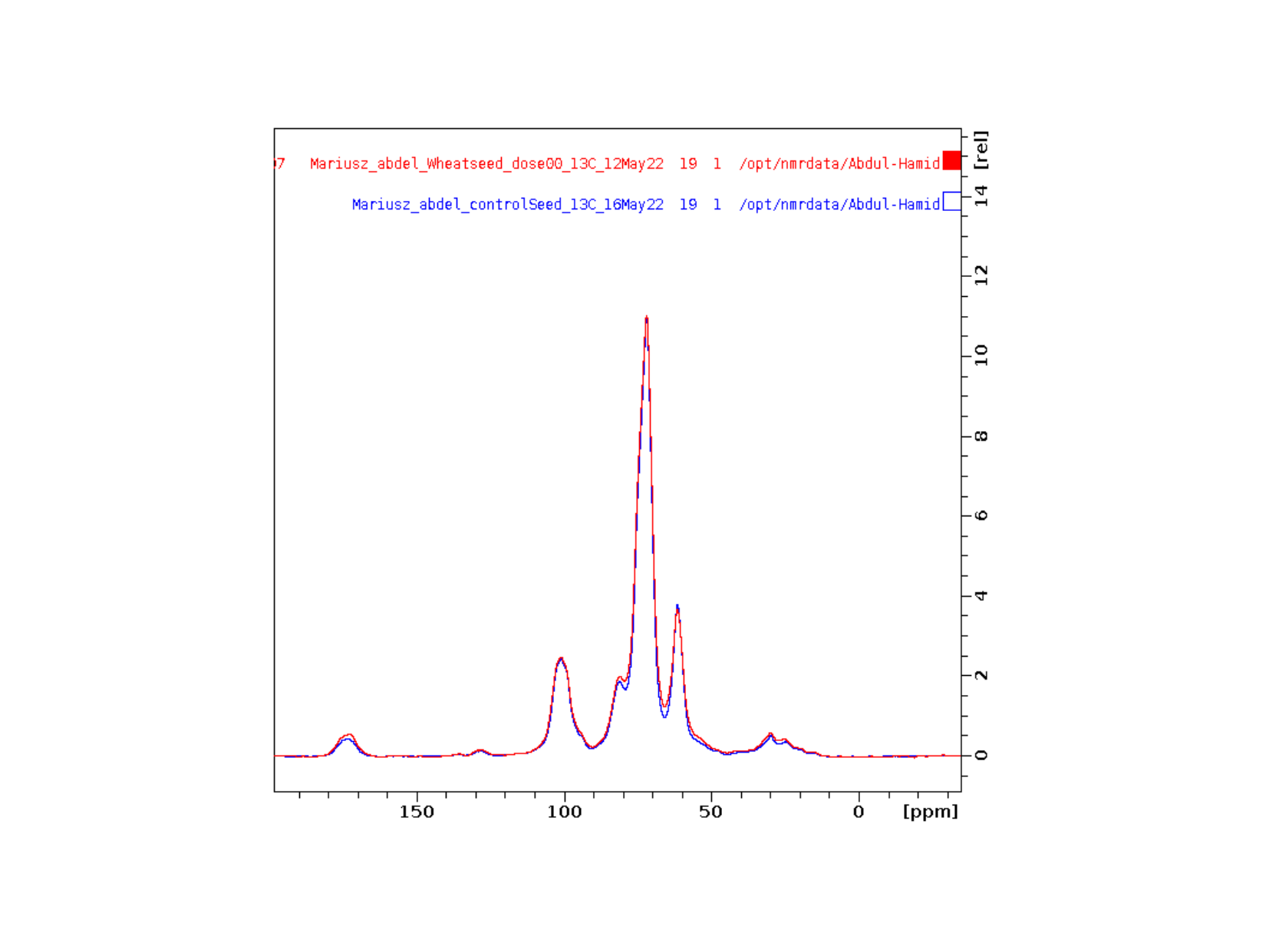

## Slide 2
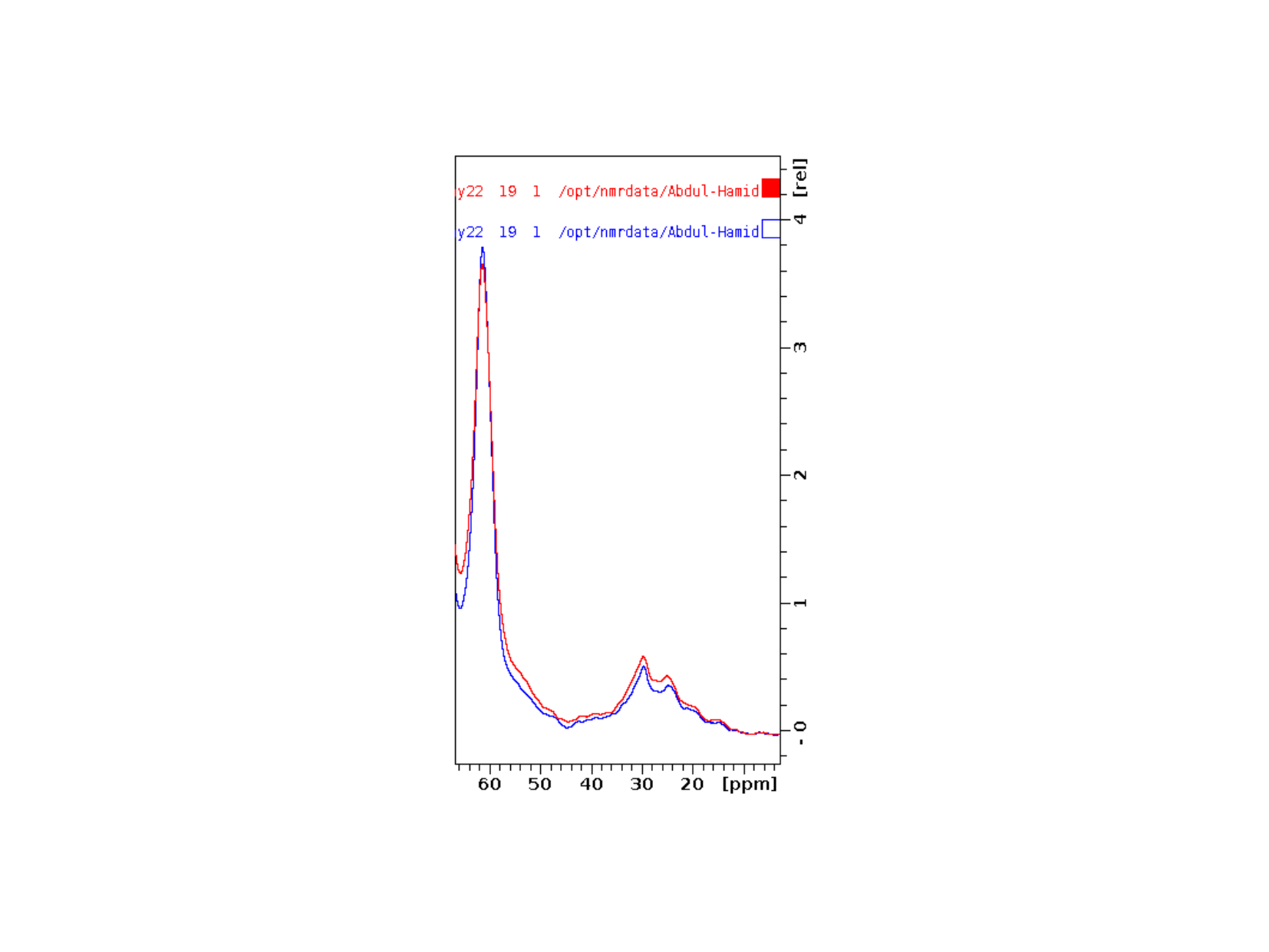

## Slide 3
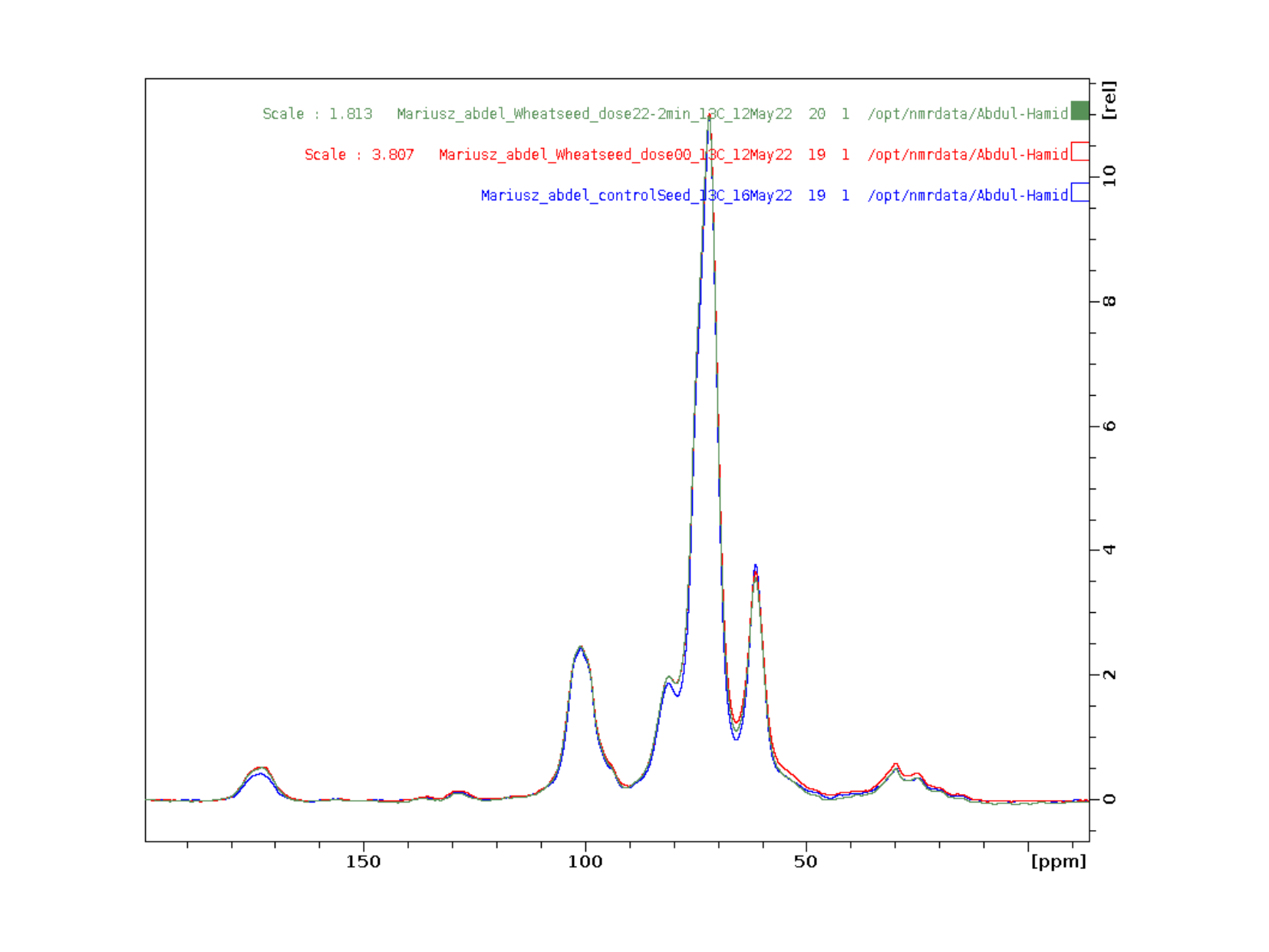

## Slide 4
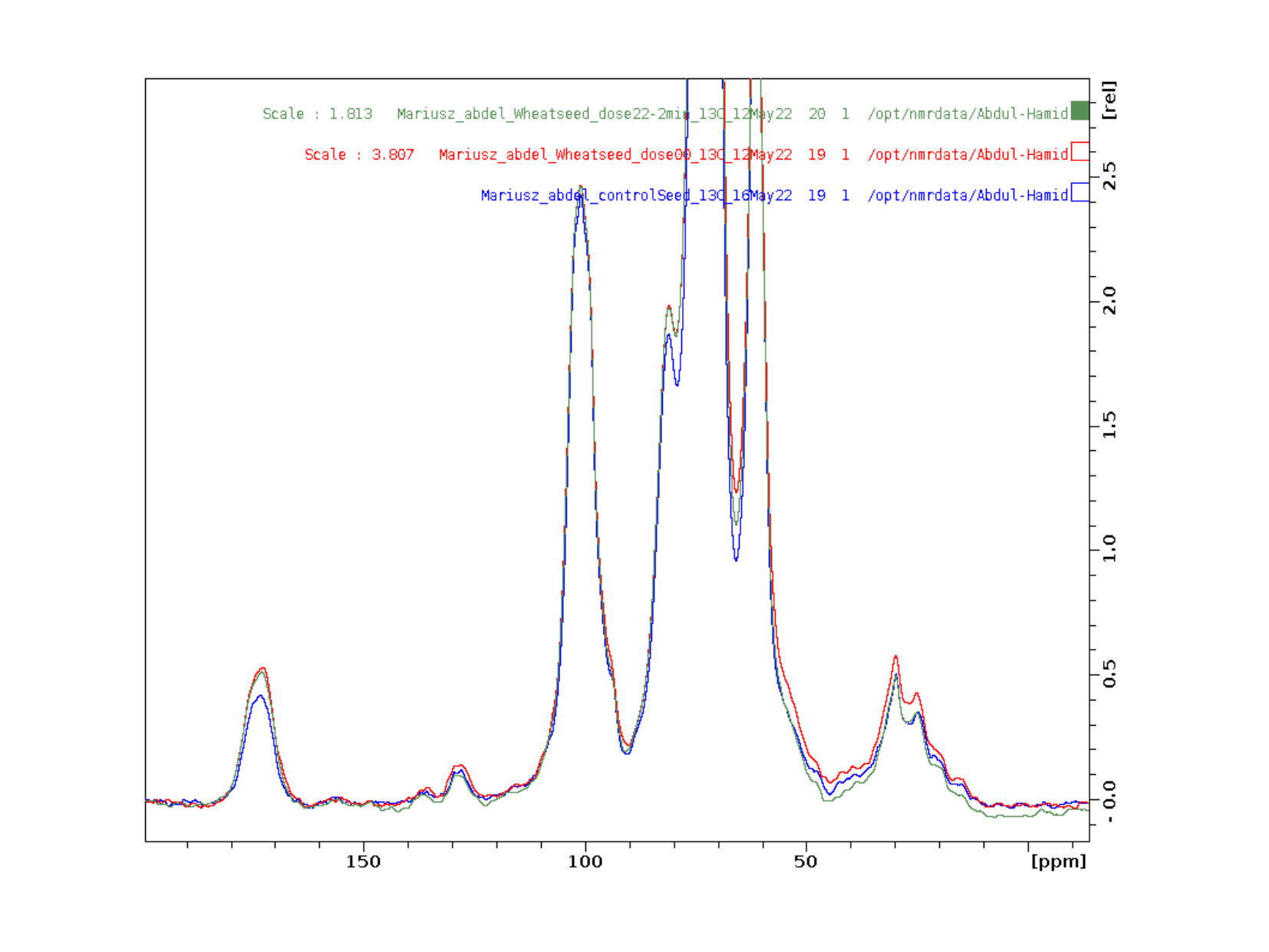

Supplement: S1 File — (ZIP) [file pone.0334274.s001.zip › Fw_ NMR data/NMR Abel-Fatah_report_17June22.pptx]

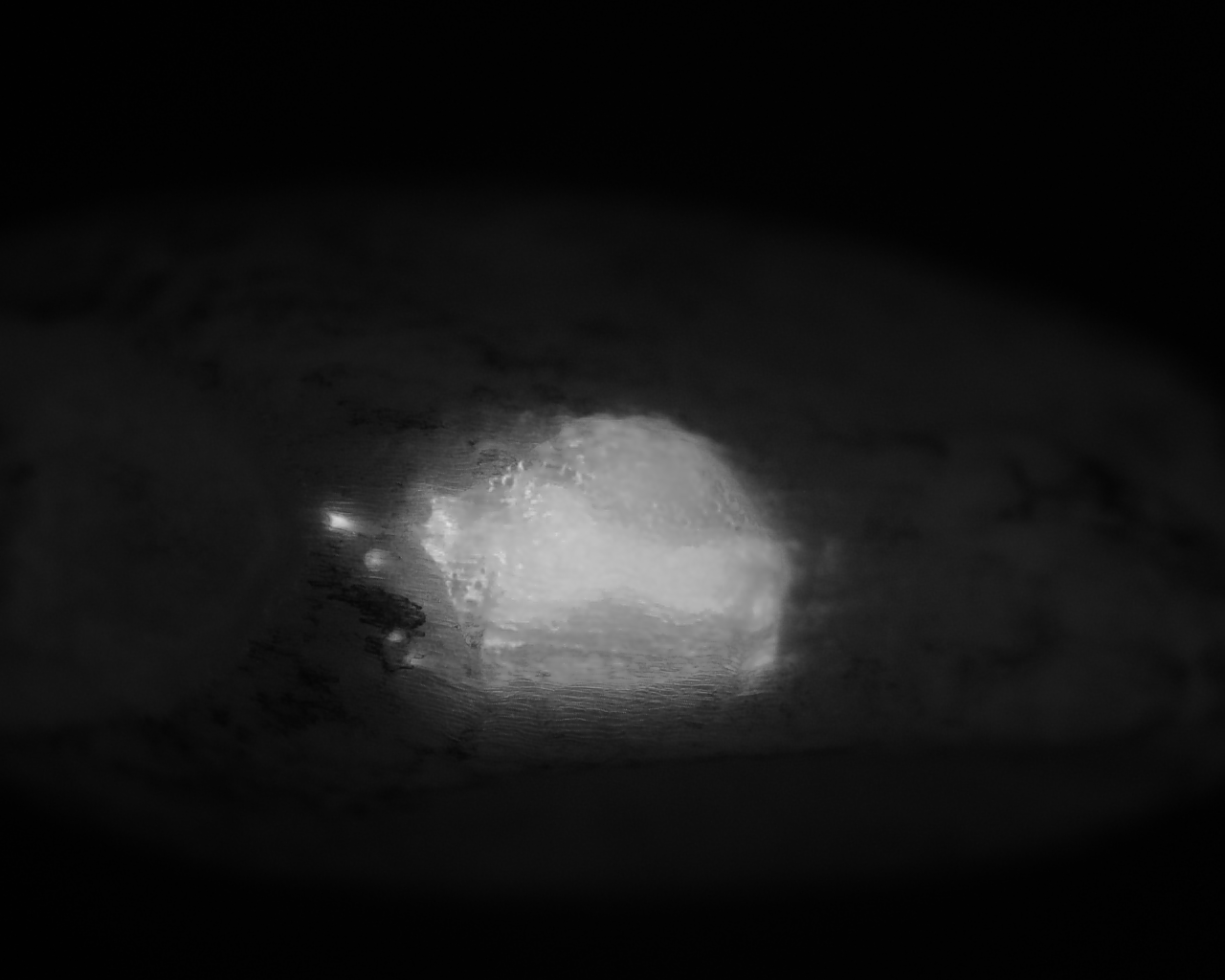

Supplement: S3 File — (ZIP) [file pone.0334274.s003.zip › control3.tif]

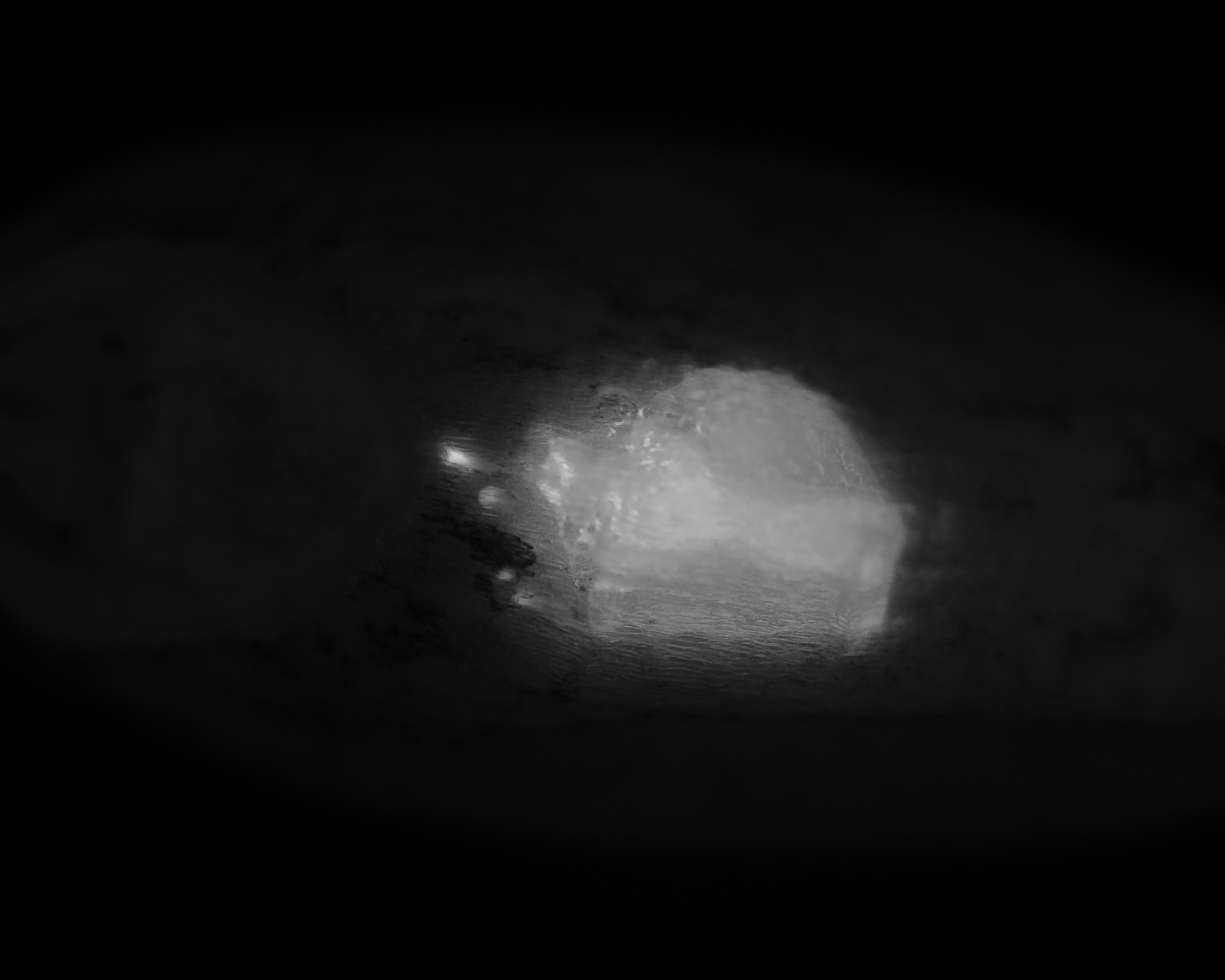

Supplement: S3 File — (ZIP) [file pone.0334274.s003.zip › Control4.tif]

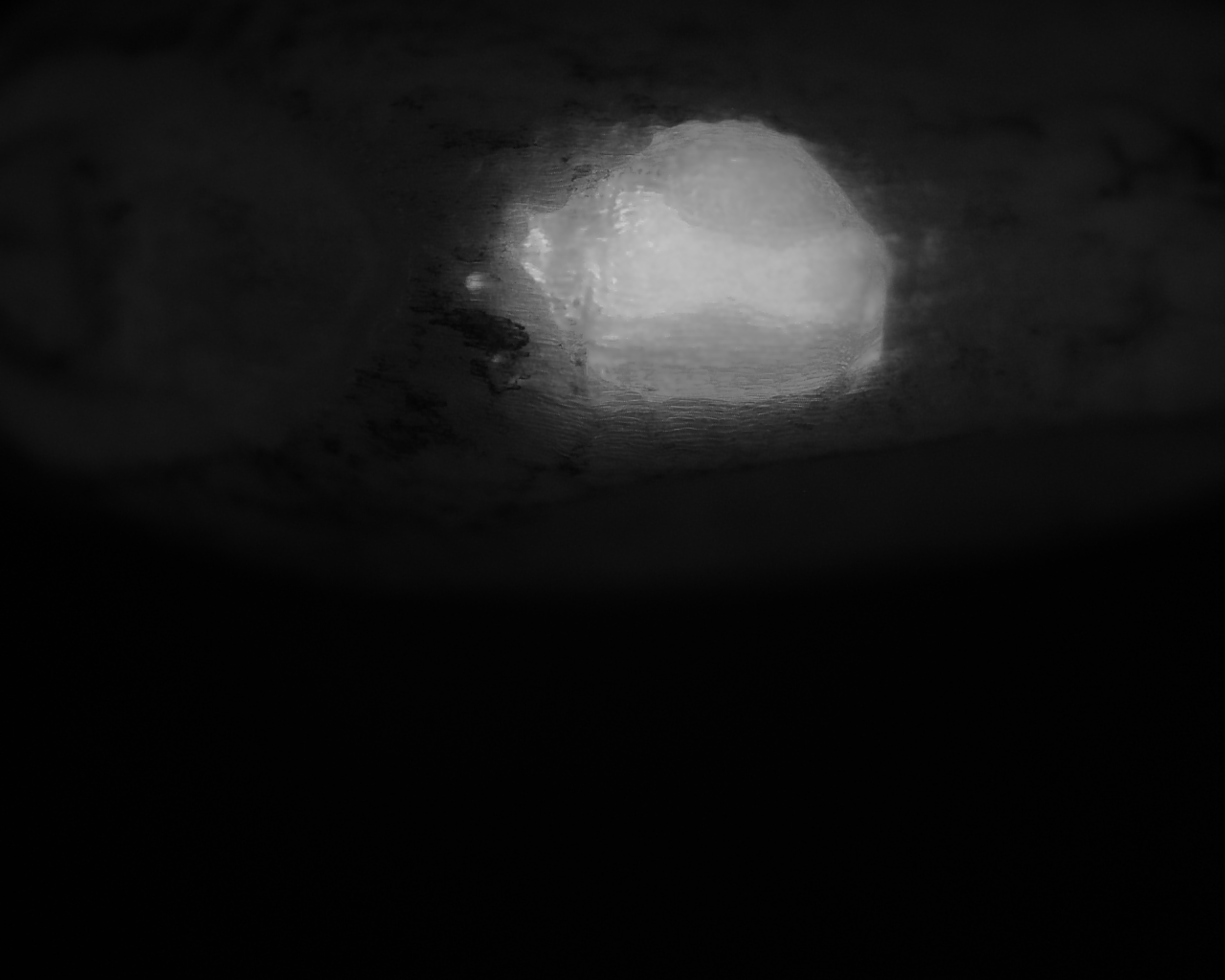

Supplement: S3 File — (ZIP) [file pone.0334274.s003.zip › ctrl.tif]

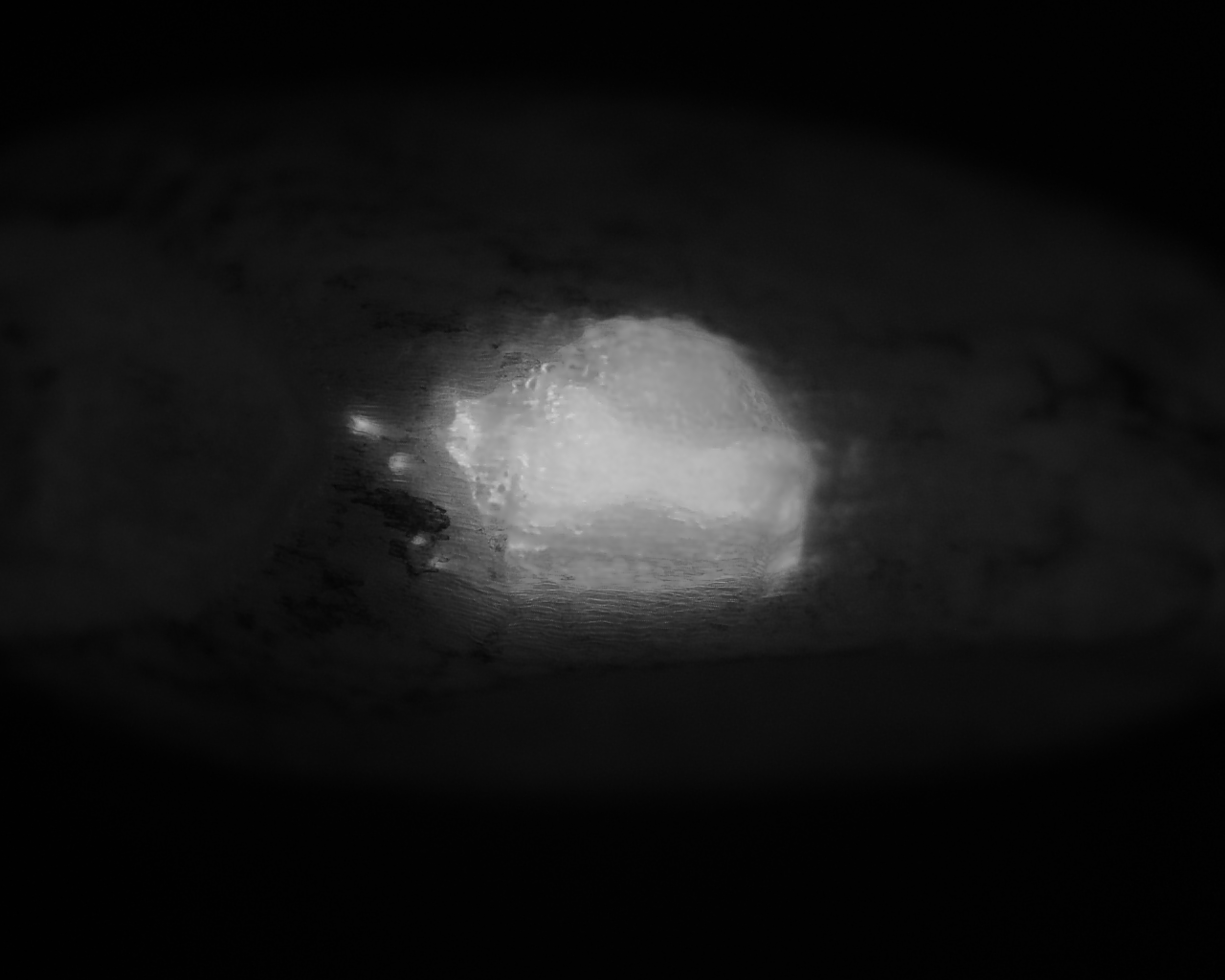

Supplement: S3 File — (ZIP) [file pone.0334274.s003.zip › ctrl2.tif]

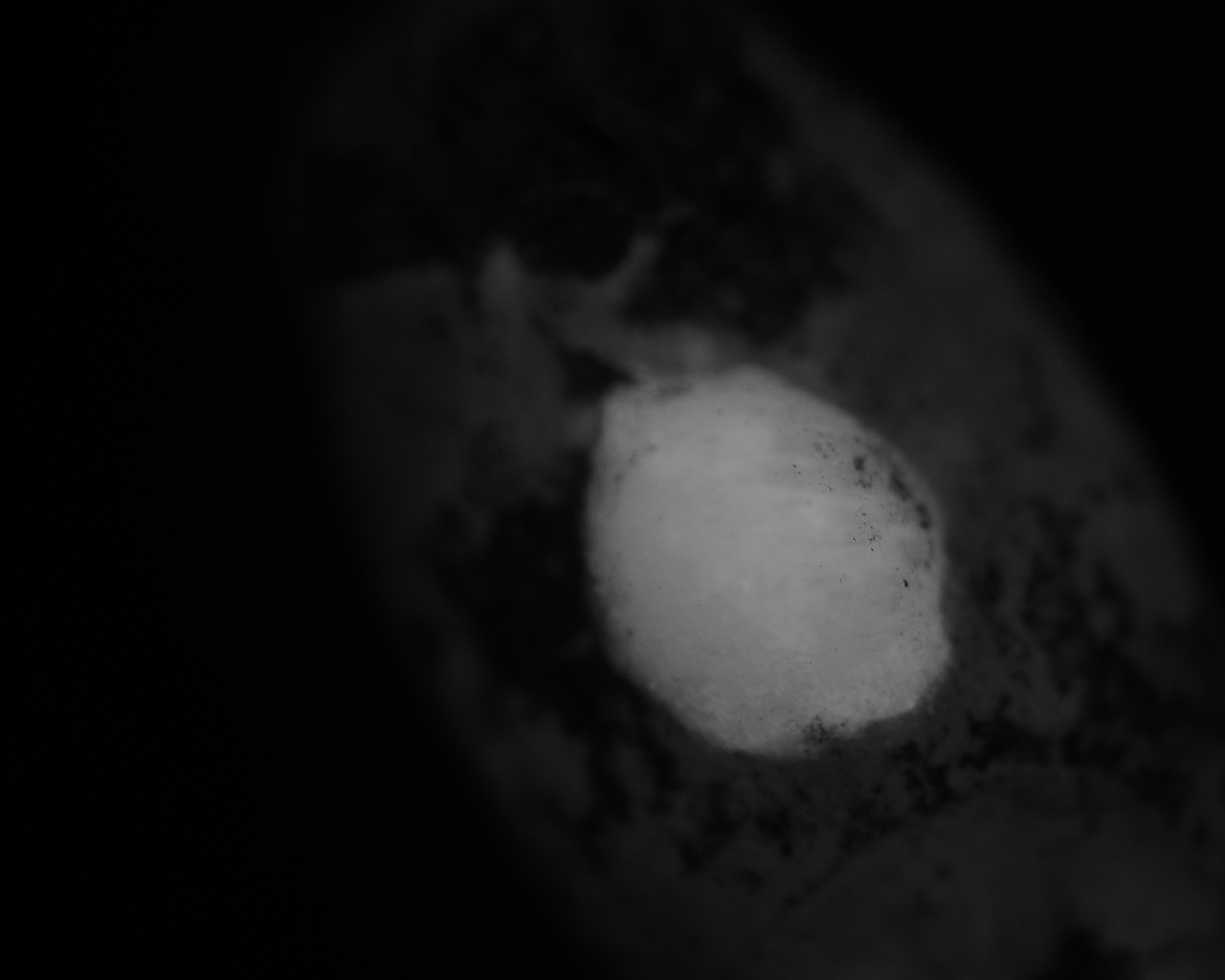

Supplement: S3 File — (ZIP) [file pone.0334274.s003.zip › D00 1.tif]

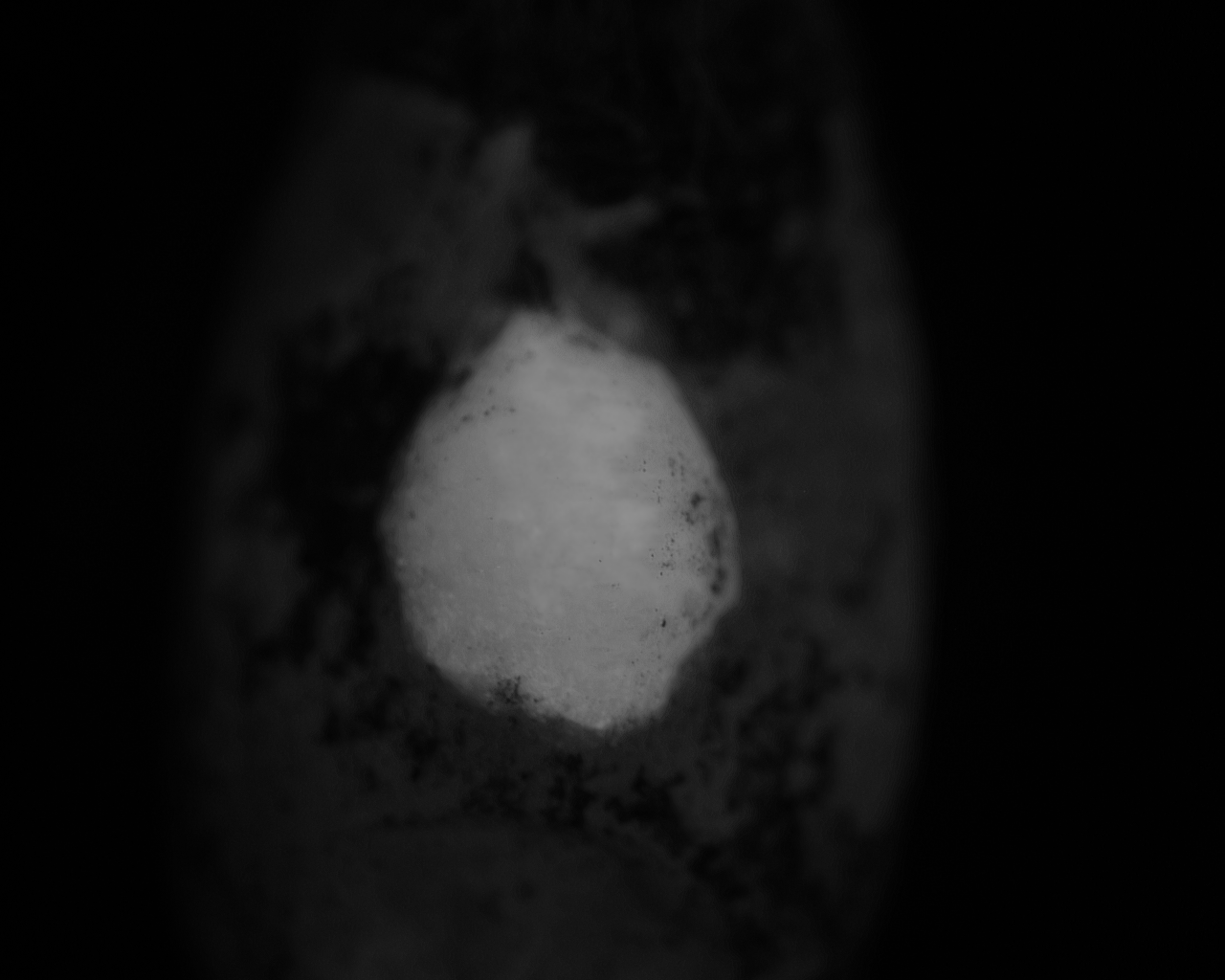

Supplement: S3 File — (ZIP) [file pone.0334274.s003.zip › D00 2.tif]

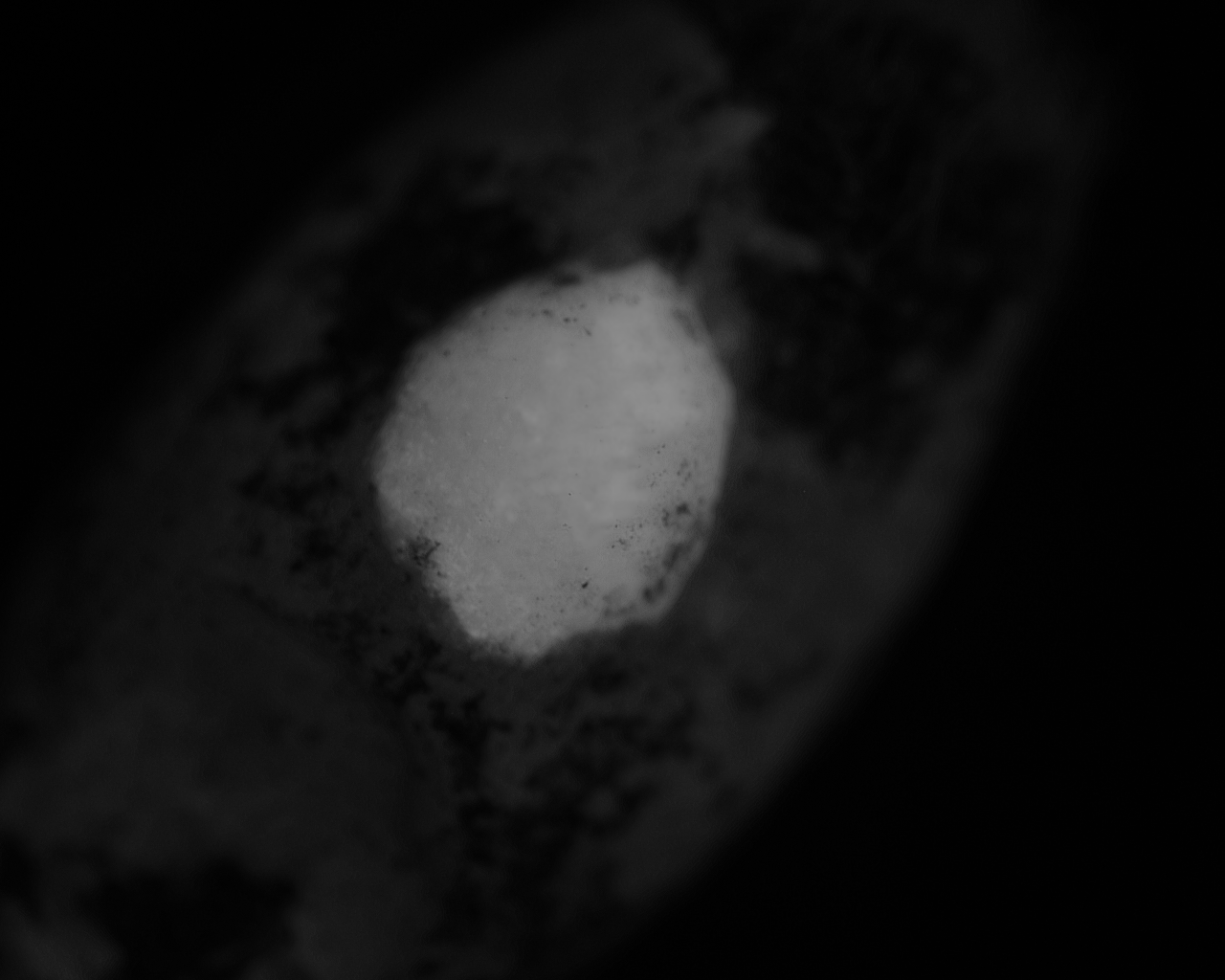

Supplement: S3 File — (ZIP) [file pone.0334274.s003.zip › D00 3.tif]

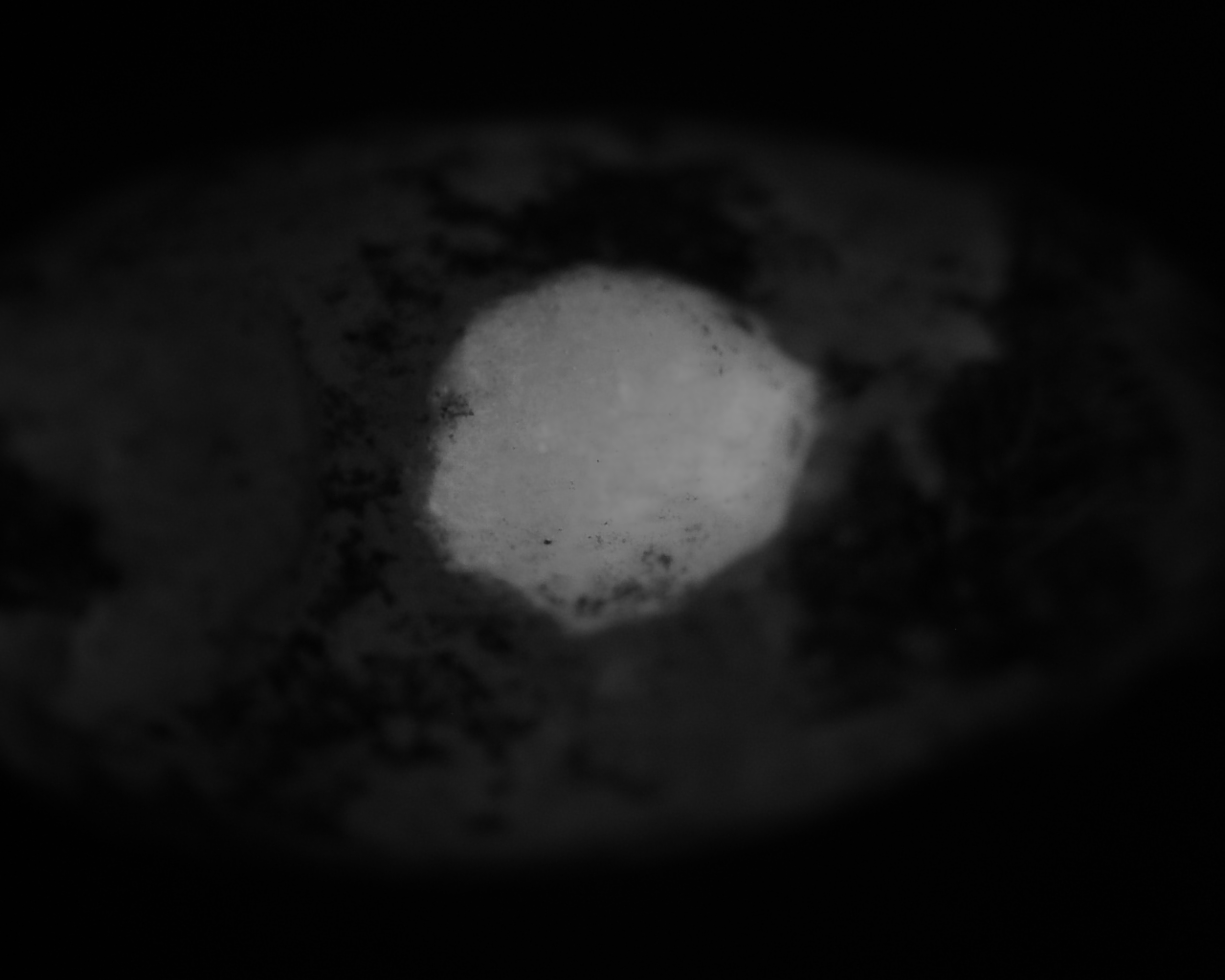

Supplement: S3 File — (ZIP) [file pone.0334274.s003.zip › D00 4.tif]

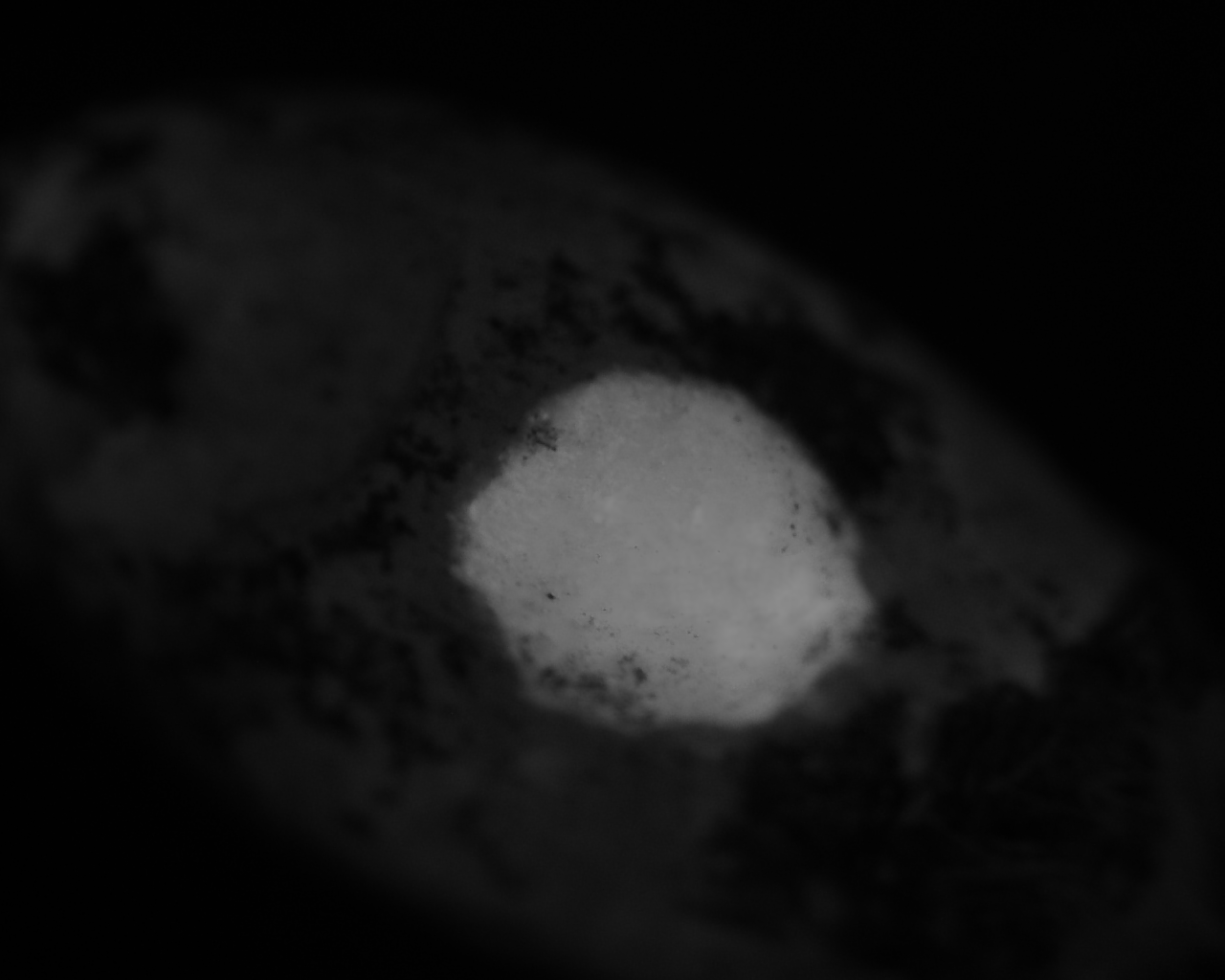

Supplement: S3 File — (ZIP) [file pone.0334274.s003.zip › D00 5.tif]

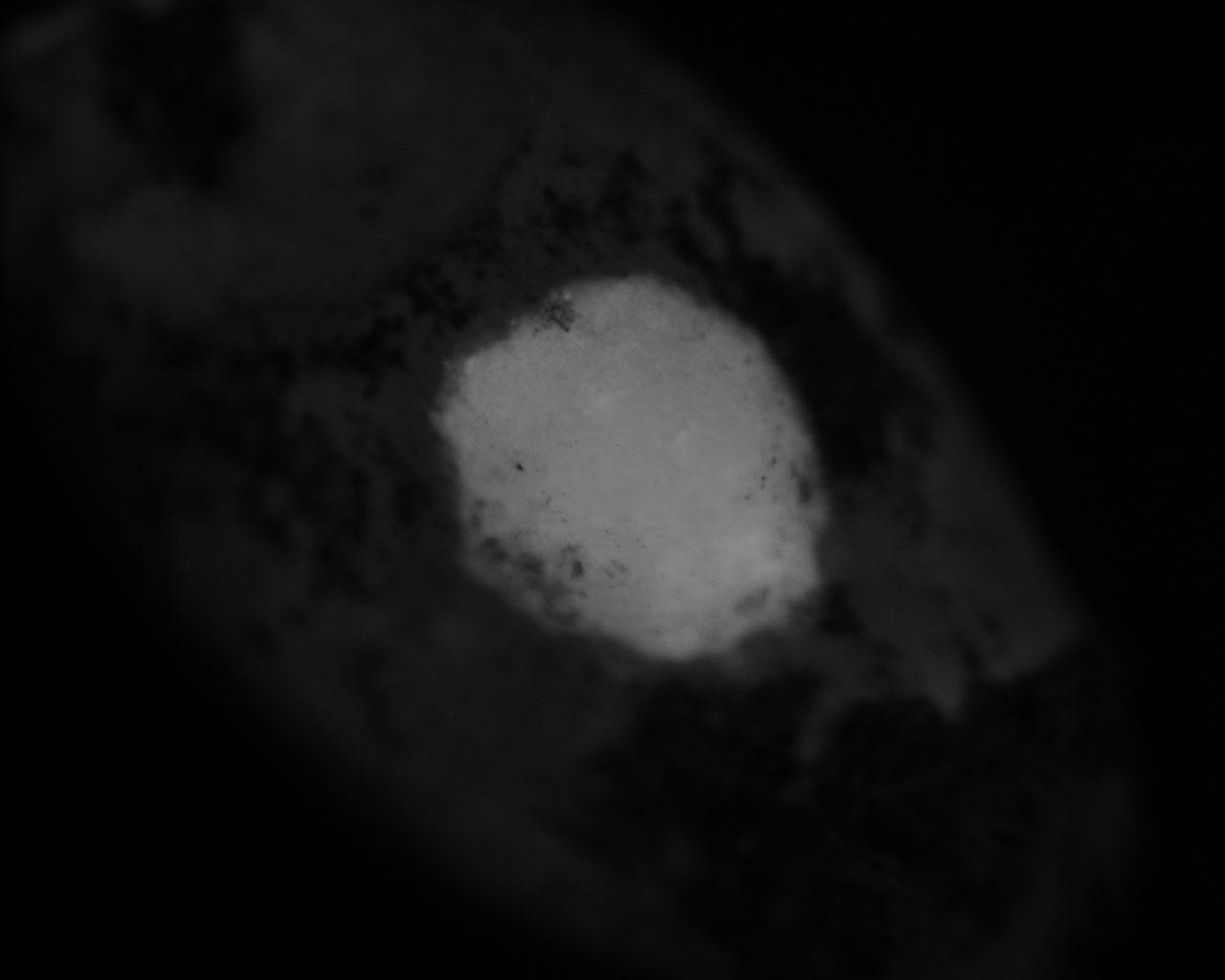

Supplement: S3 File — (ZIP) [file pone.0334274.s003.zip › D00 6.tif]

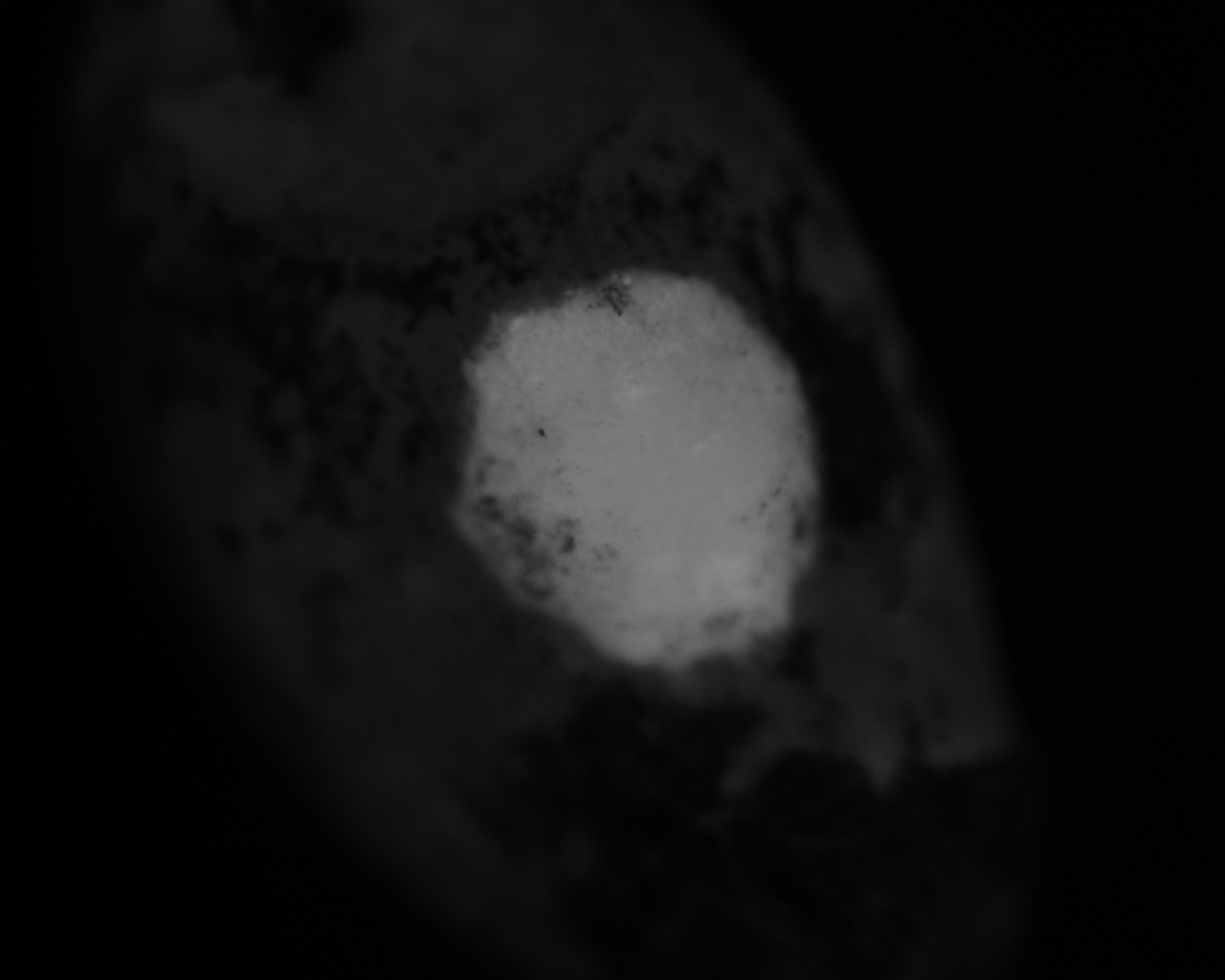

Supplement: S3 File — (ZIP) [file pone.0334274.s003.zip › D00 7.tif]

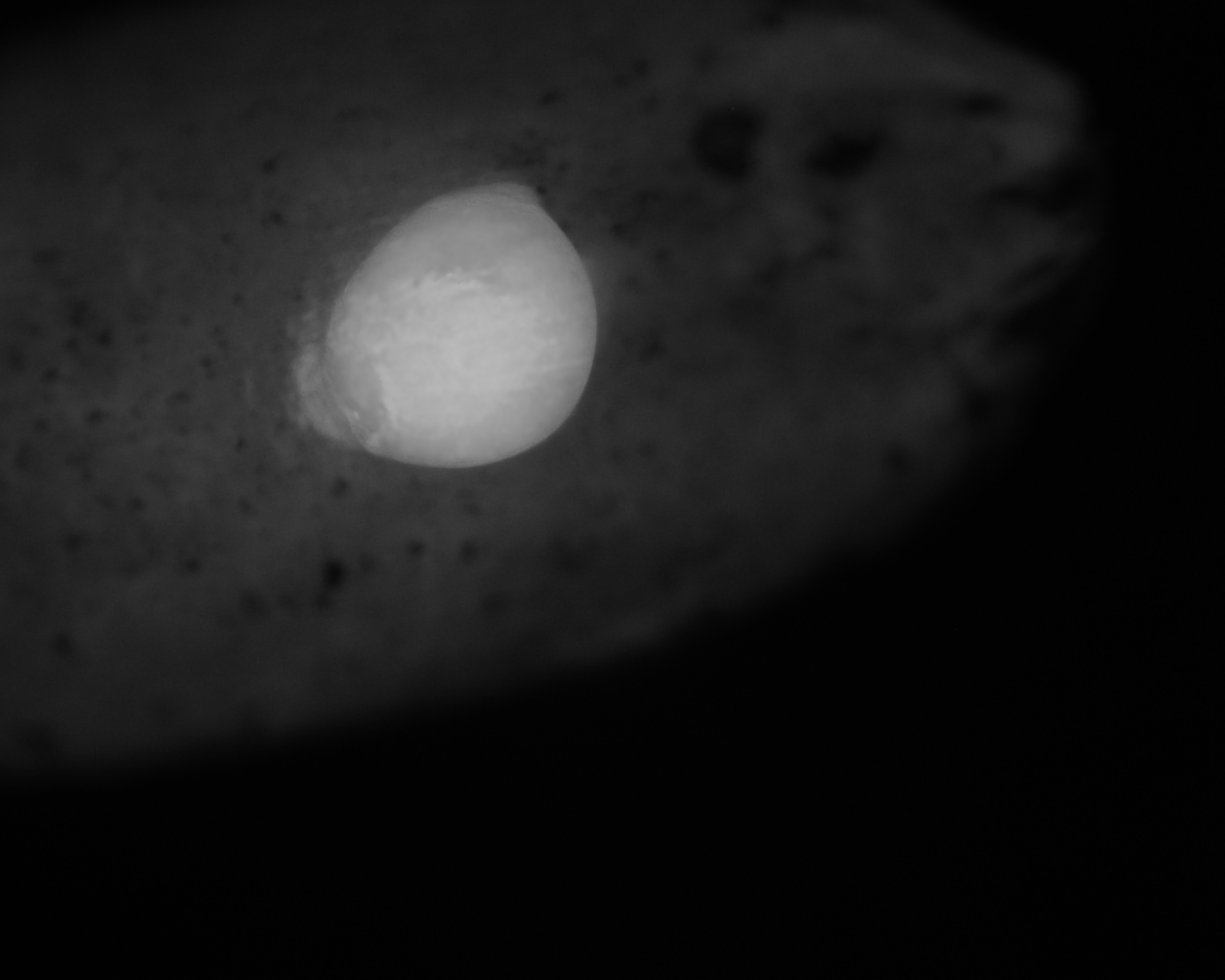

Supplement: S3 File — (ZIP) [file pone.0334274.s003.zip › D22 1.tif]

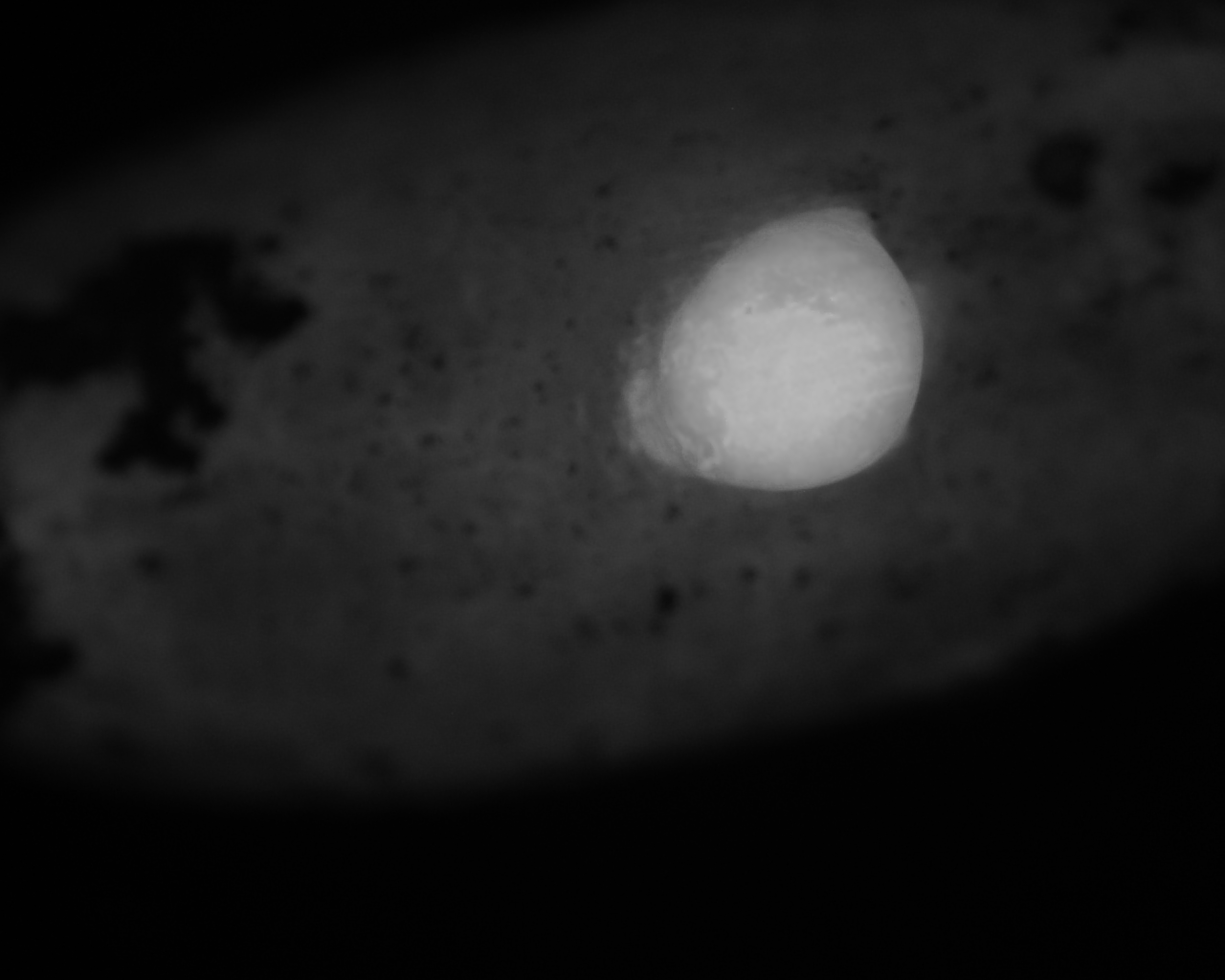

Supplement: S3 File — (ZIP) [file pone.0334274.s003.zip › D22 2.tif]

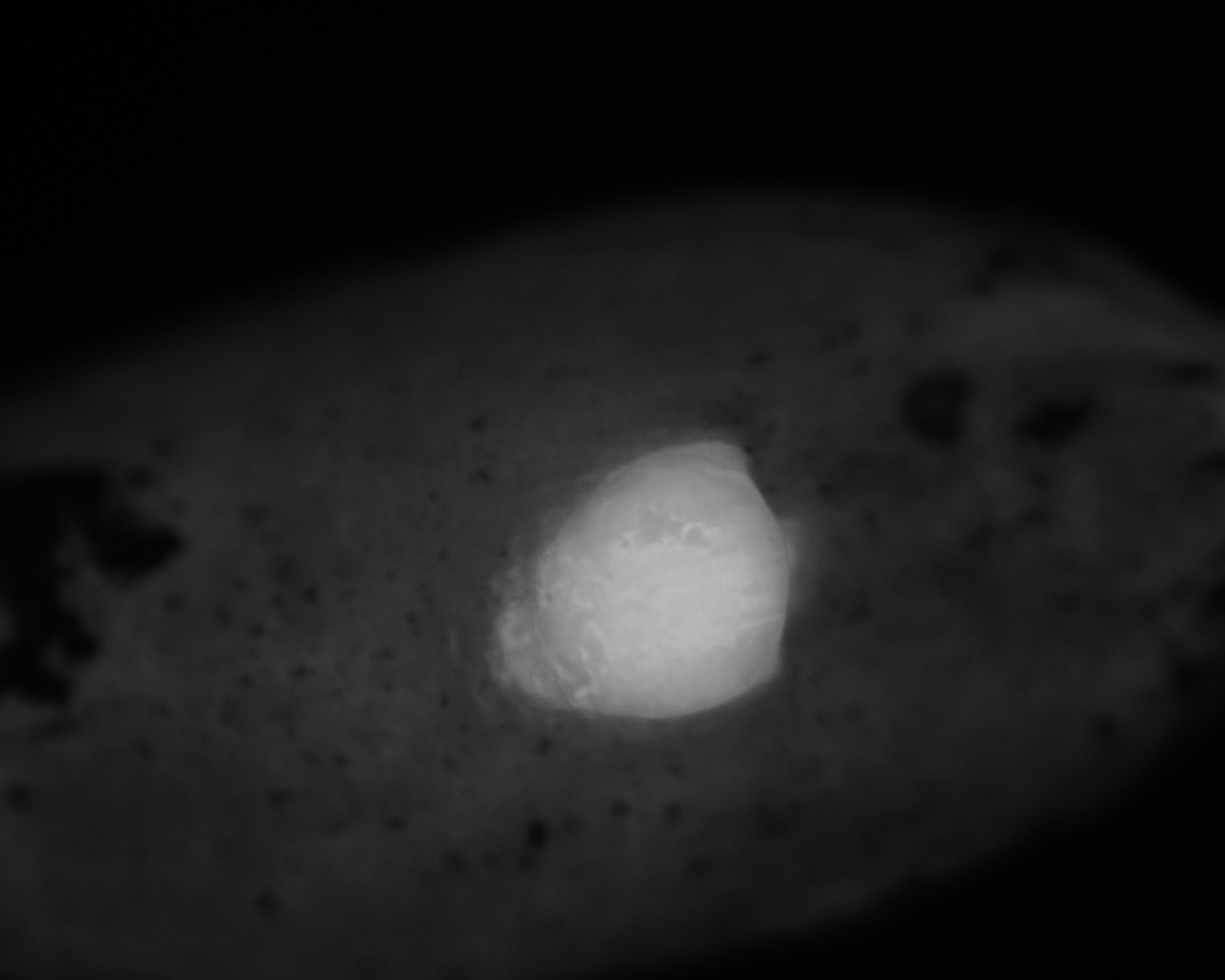

Supplement: S3 File — (ZIP) [file pone.0334274.s003.zip › D22 3.tif]

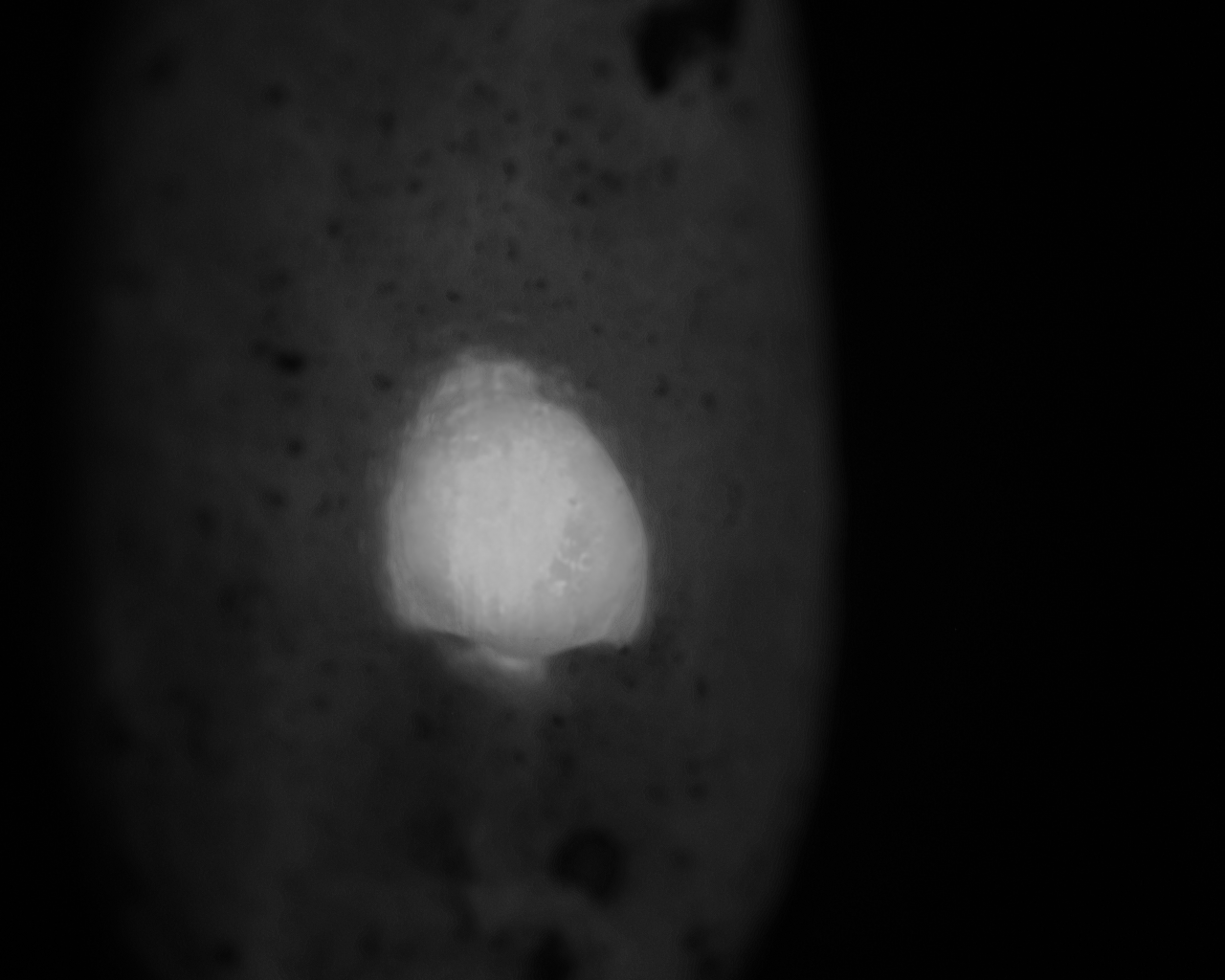

Supplement: S3 File — (ZIP) [file pone.0334274.s003.zip › D22 4.tif]

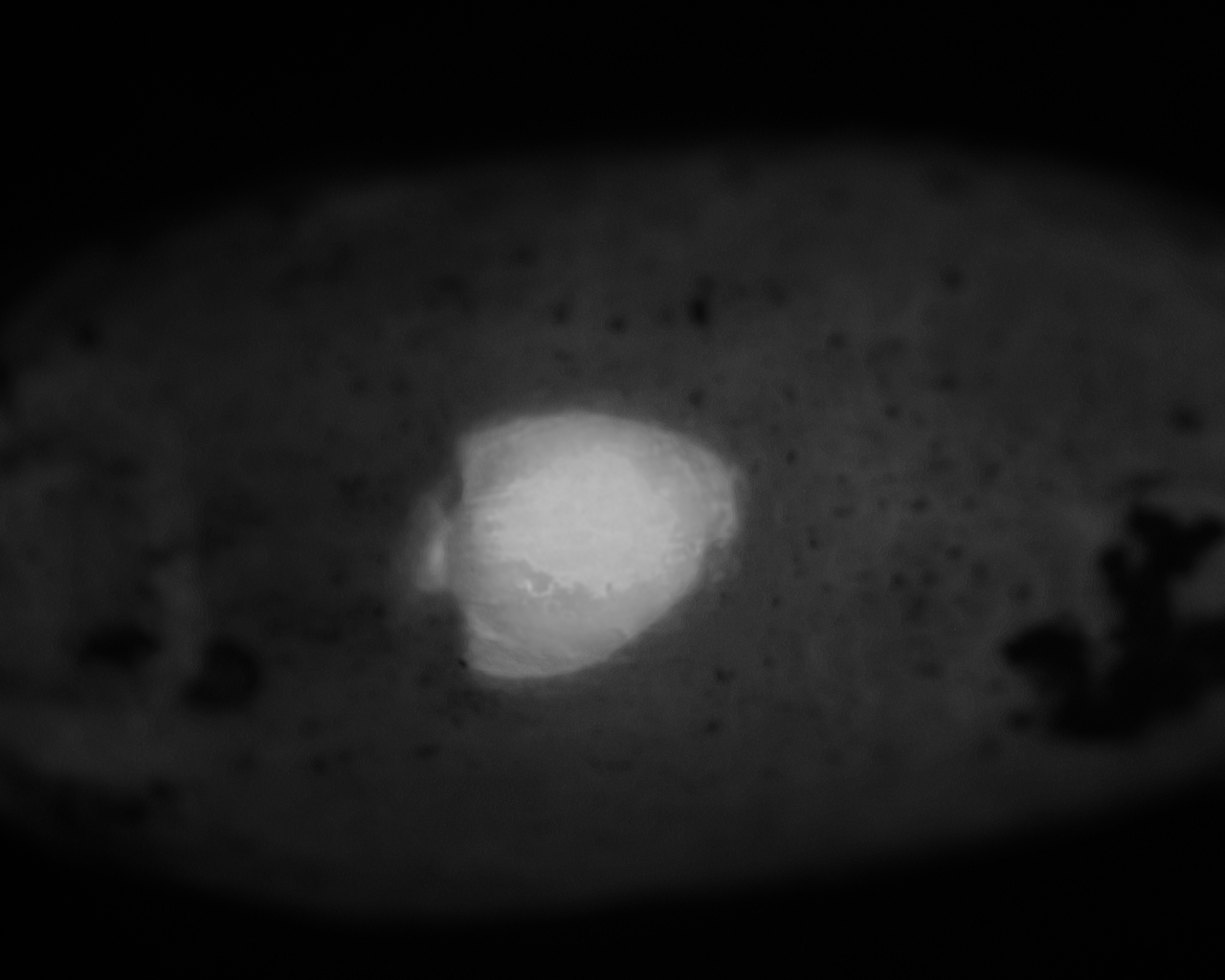

Supplement: S3 File — (ZIP) [file pone.0334274.s003.zip › D22 5.tif]

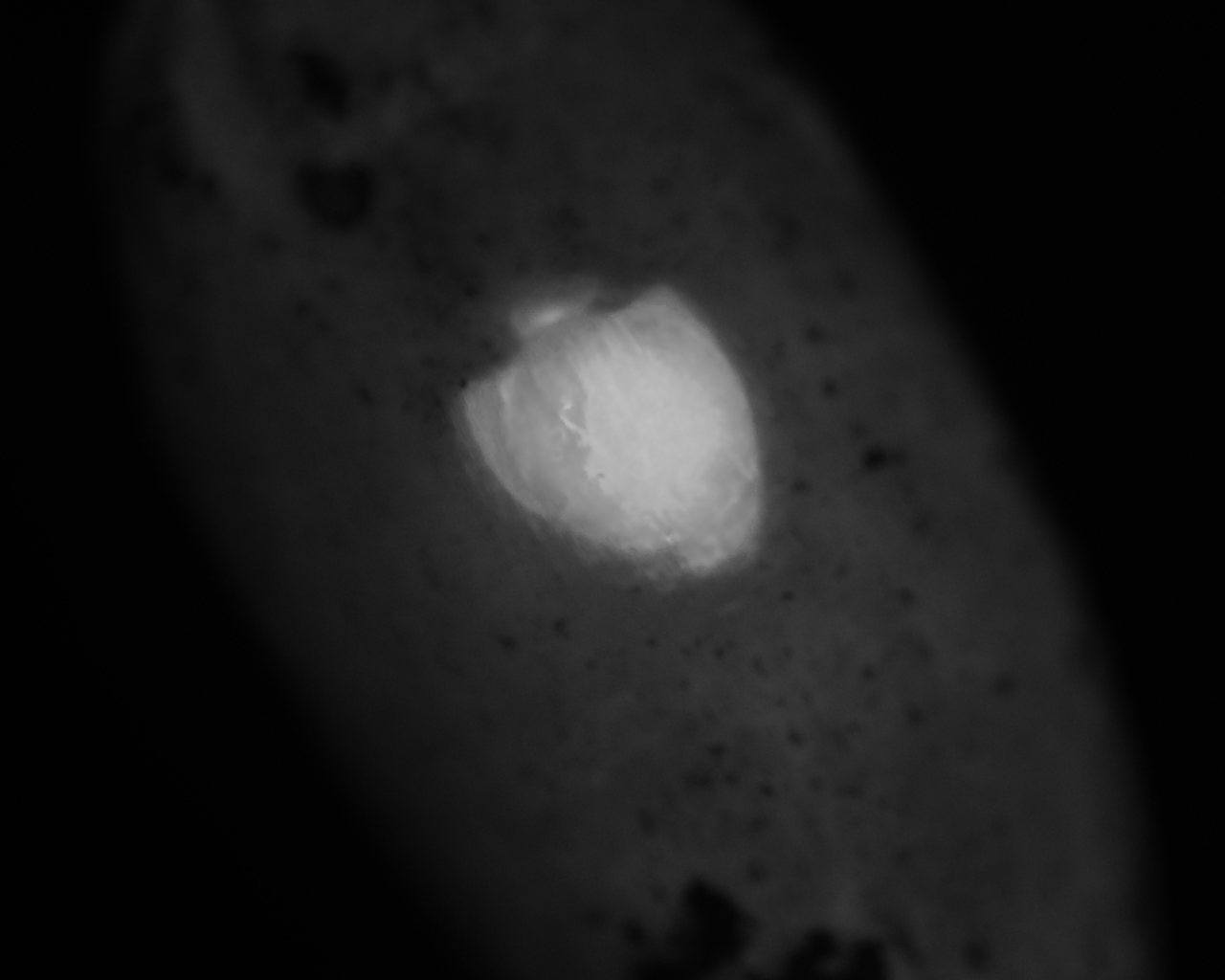

Supplement: S3 File — (ZIP) [file pone.0334274.s003.zip › D22 6.tif]

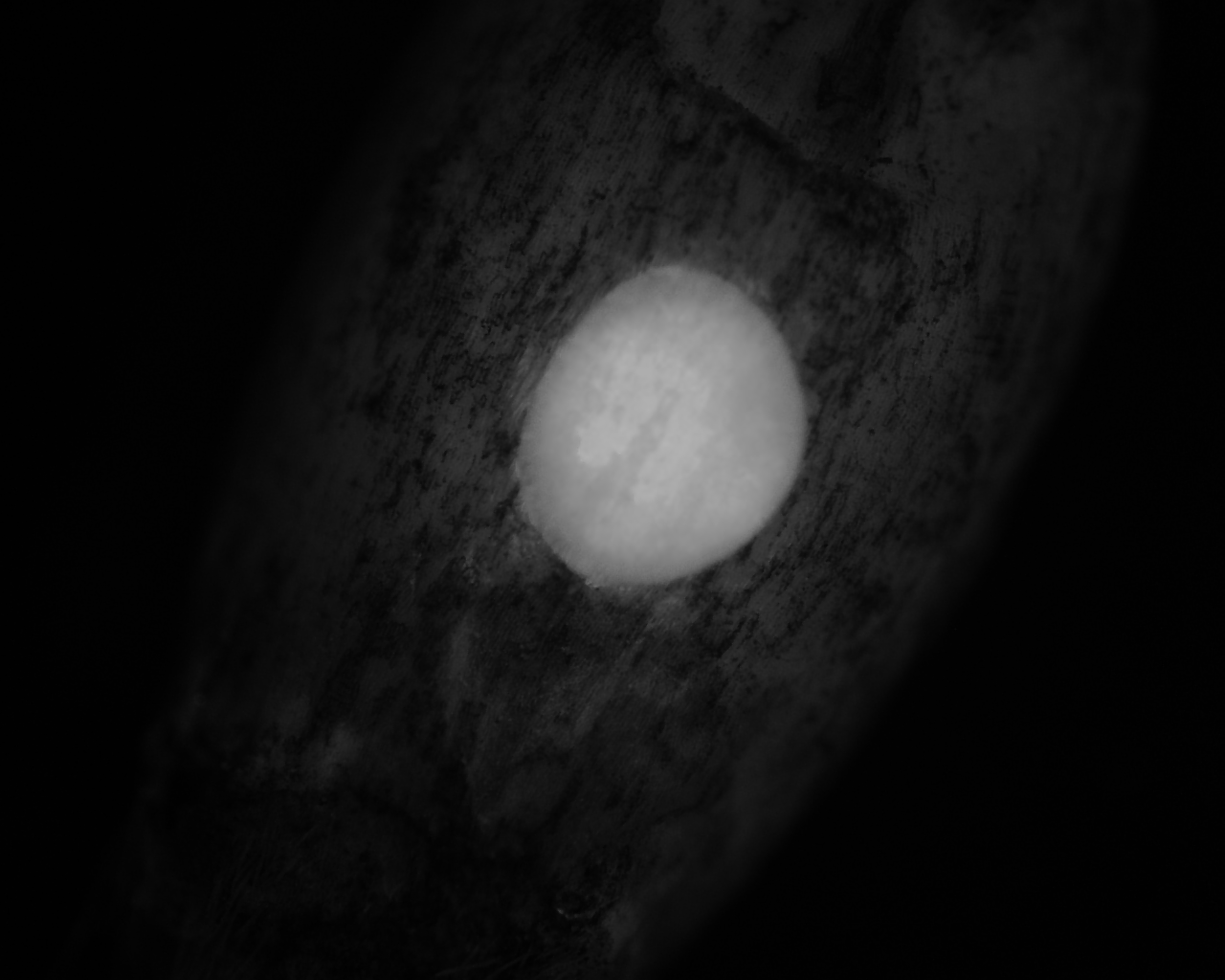

Supplement: S3 File — (ZIP) [file pone.0334274.s003.zip › D33 1.tif]

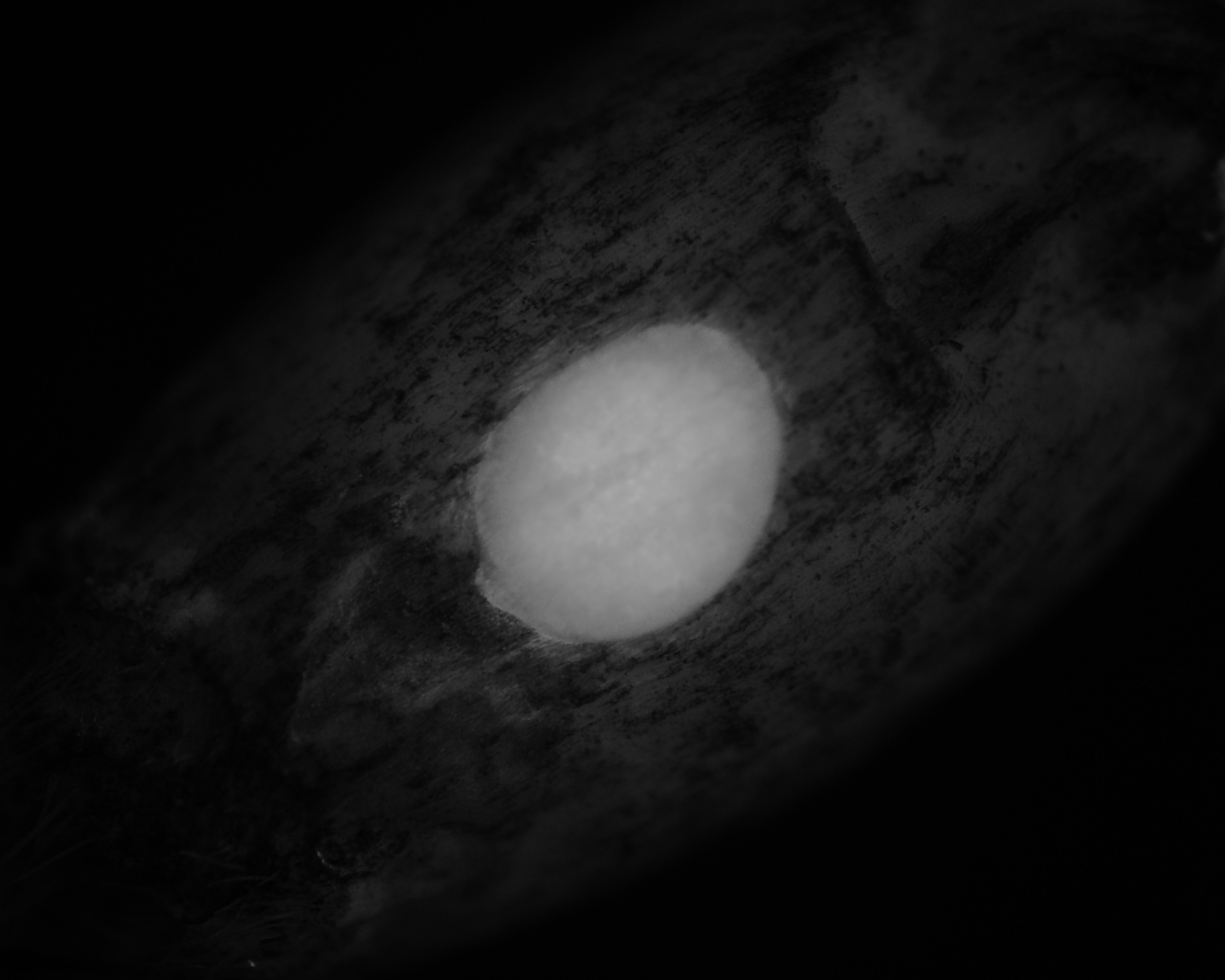

Supplement: S3 File — (ZIP) [file pone.0334274.s003.zip › D33 2.tif]

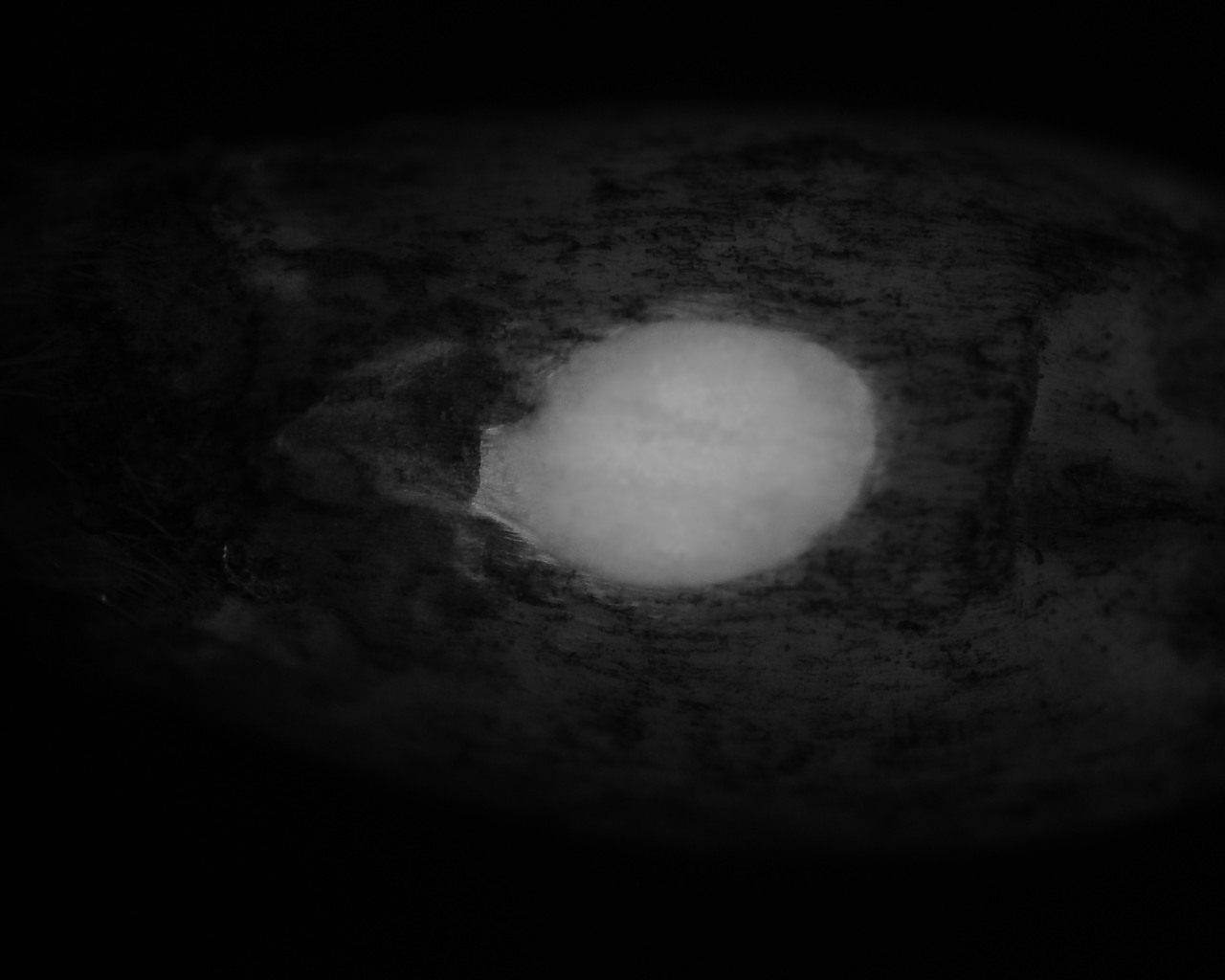

Supplement: S3 File — (ZIP) [file pone.0334274.s003.zip › D33 3.tif]

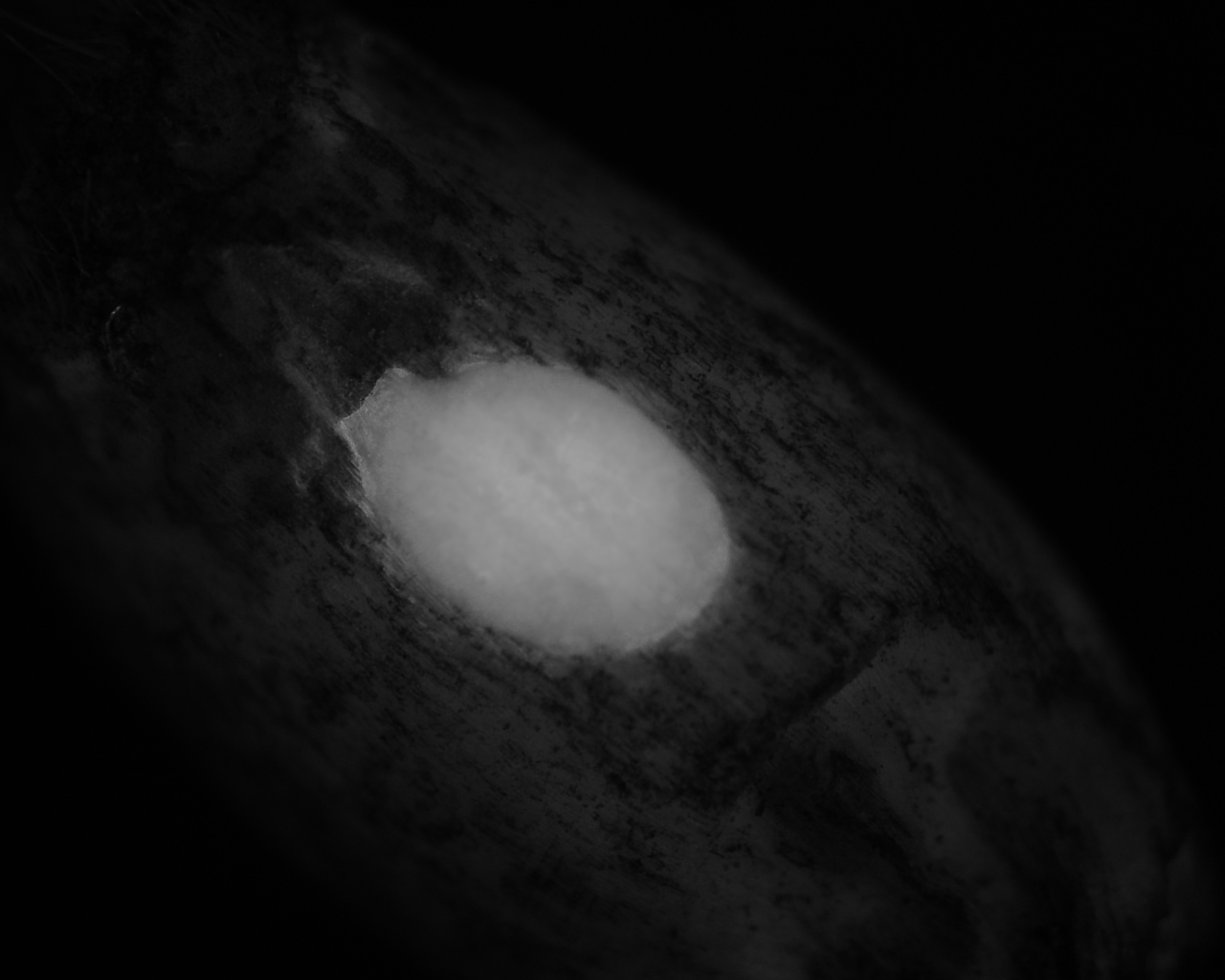

Supplement: S3 File — (ZIP) [file pone.0334274.s003.zip › D33 4.tif]

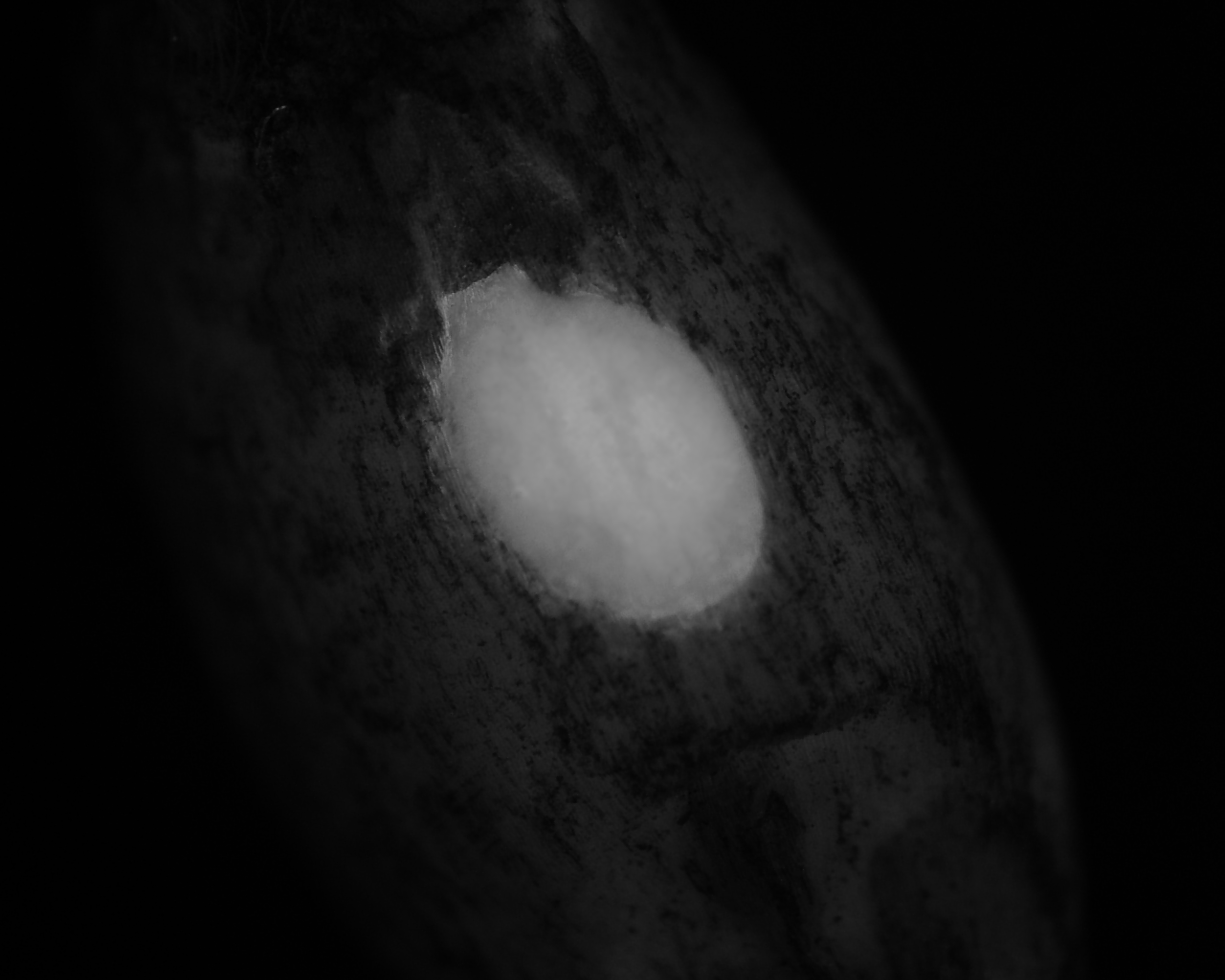

Supplement: S3 File — (ZIP) [file pone.0334274.s003.zip › D33 5.tif]

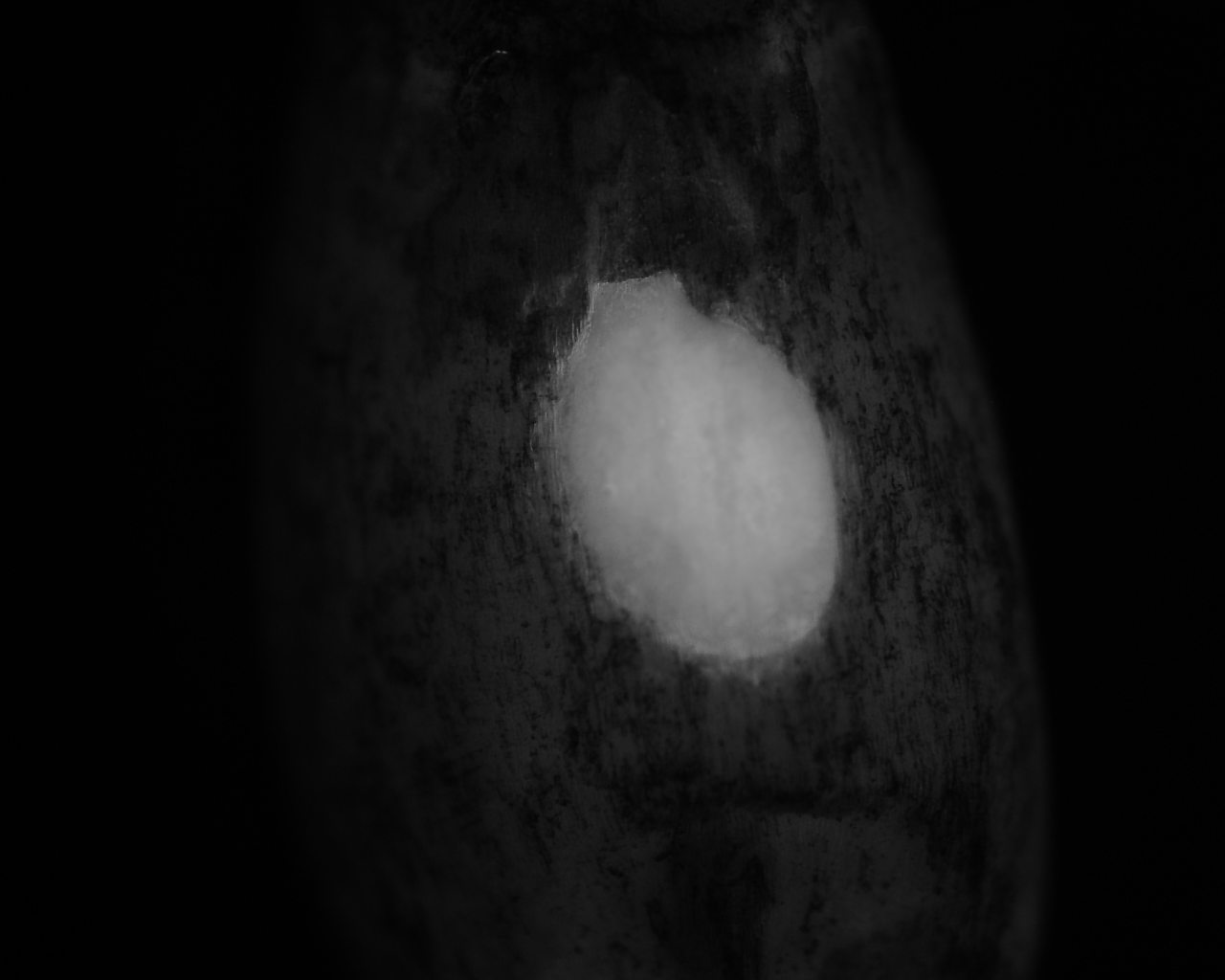

Supplement: S3 File — (ZIP) [file pone.0334274.s003.zip › D33 6.tif]

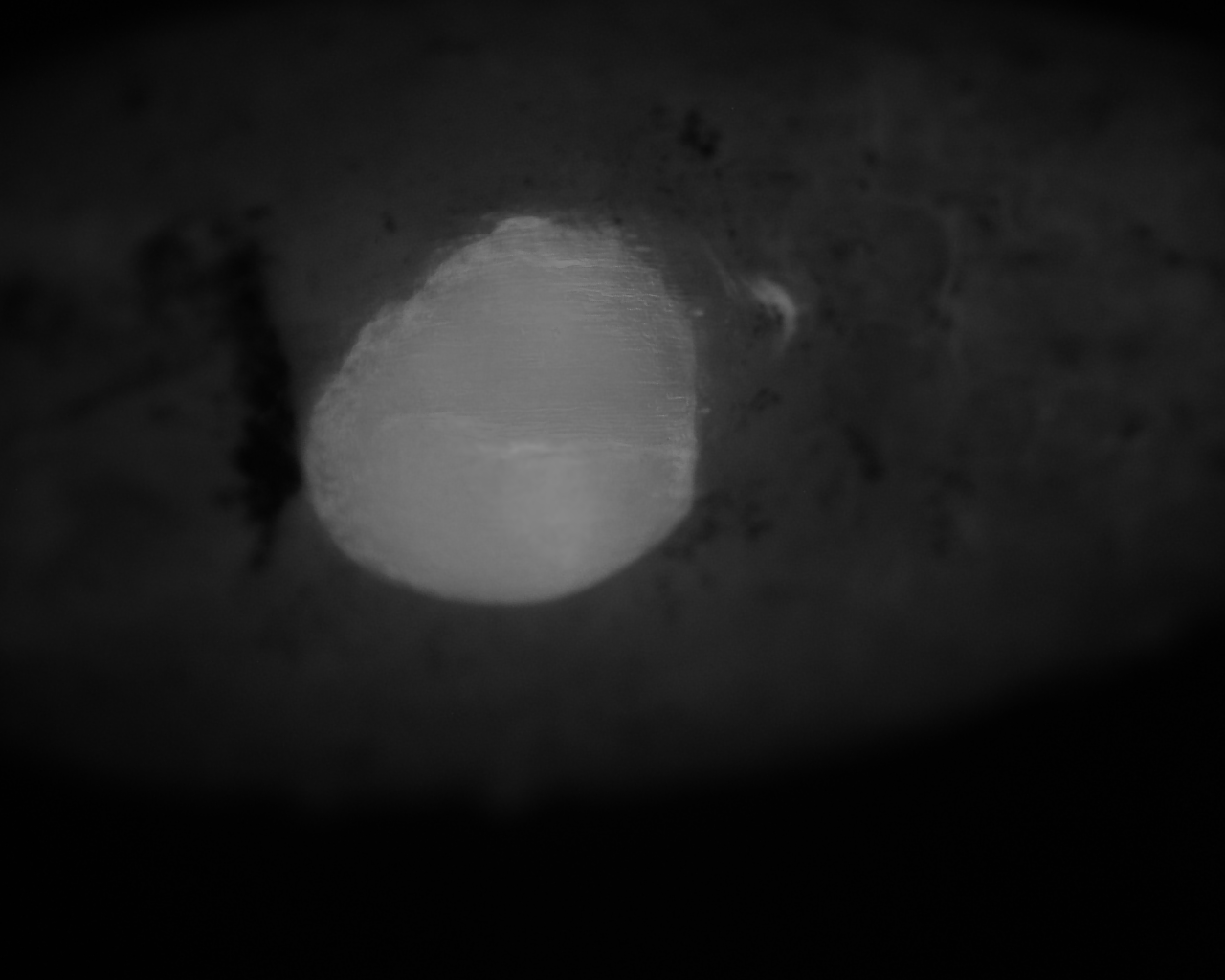

Supplement: S3 File — (ZIP) [file pone.0334274.s003.zip › D44 1.tif]

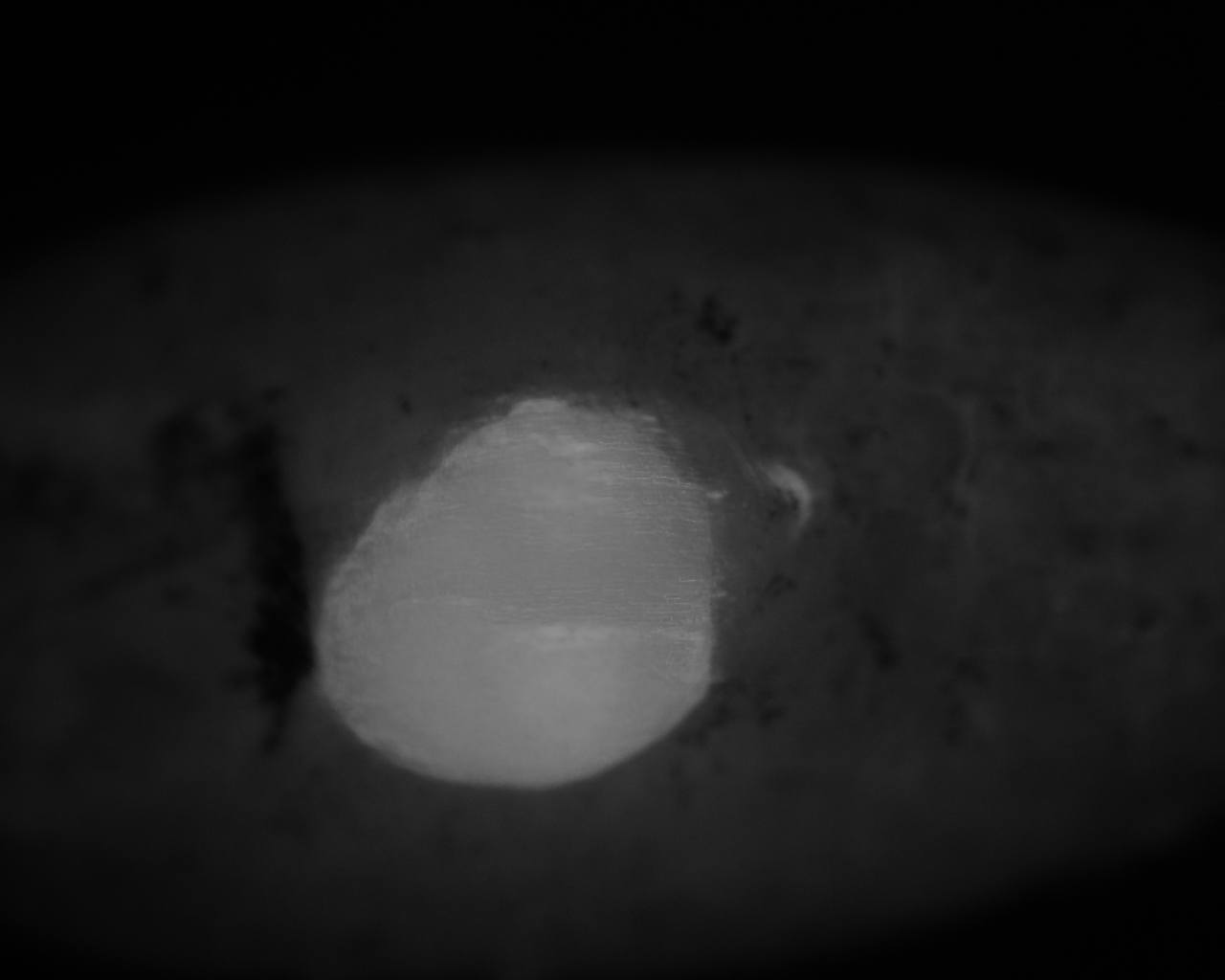

Supplement: S3 File — (ZIP) [file pone.0334274.s003.zip › D44 2.tif]

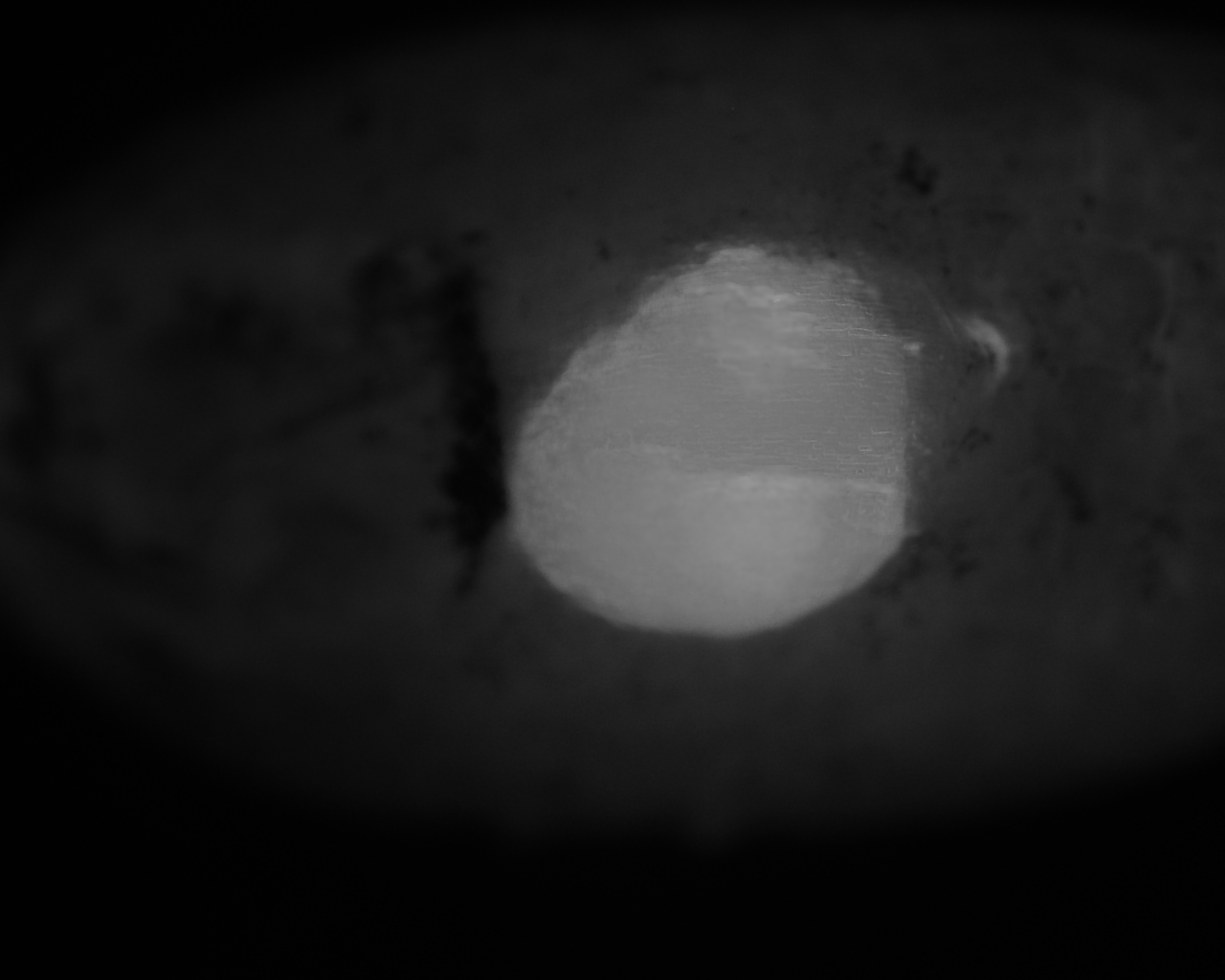

Supplement: S3 File — (ZIP) [file pone.0334274.s003.zip › D44 3.tif]

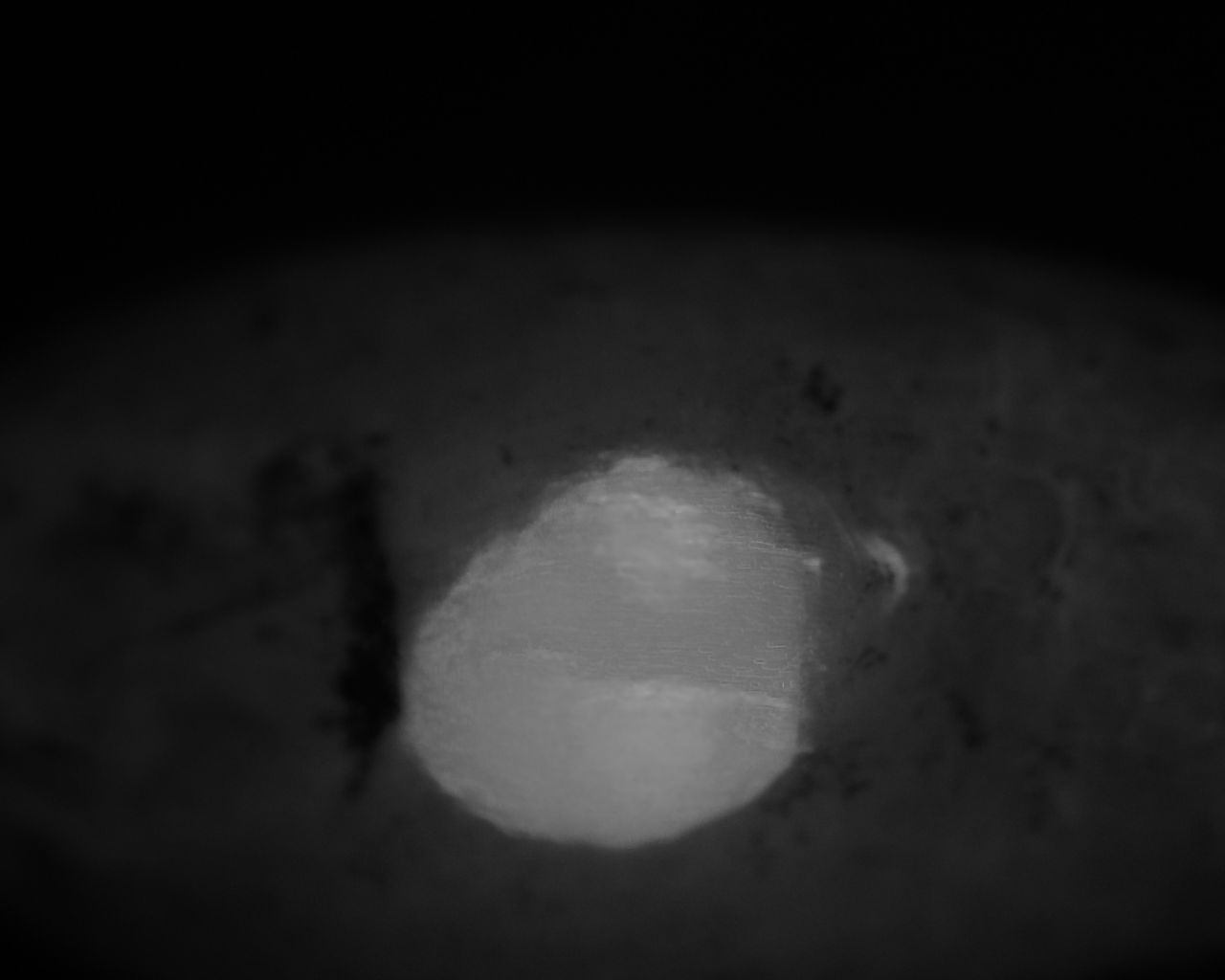

Supplement: S3 File — (ZIP) [file pone.0334274.s003.zip › D44 4.tif]

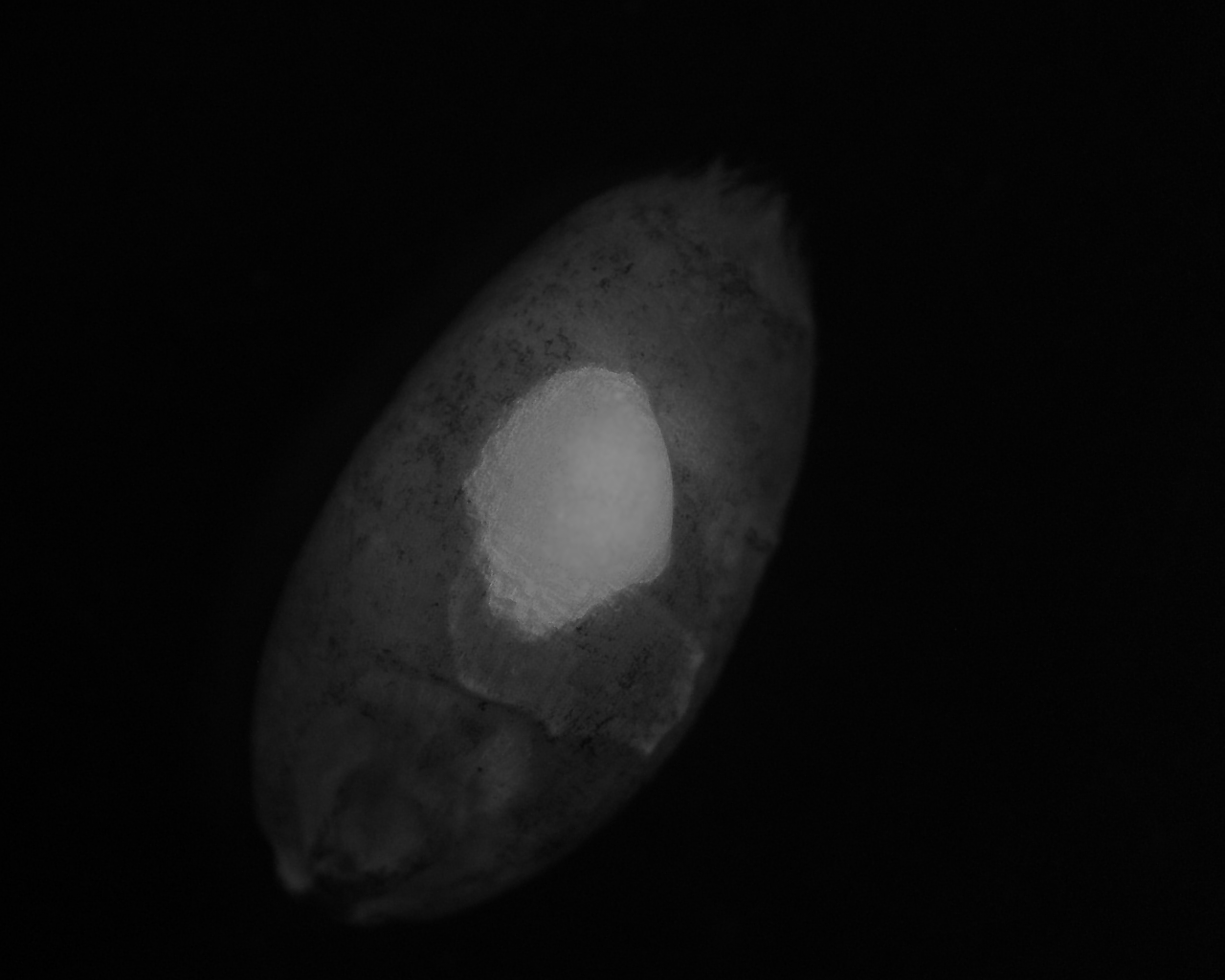

Supplement: S3 File — (ZIP) [file pone.0334274.s003.zip › D55 1.tif]

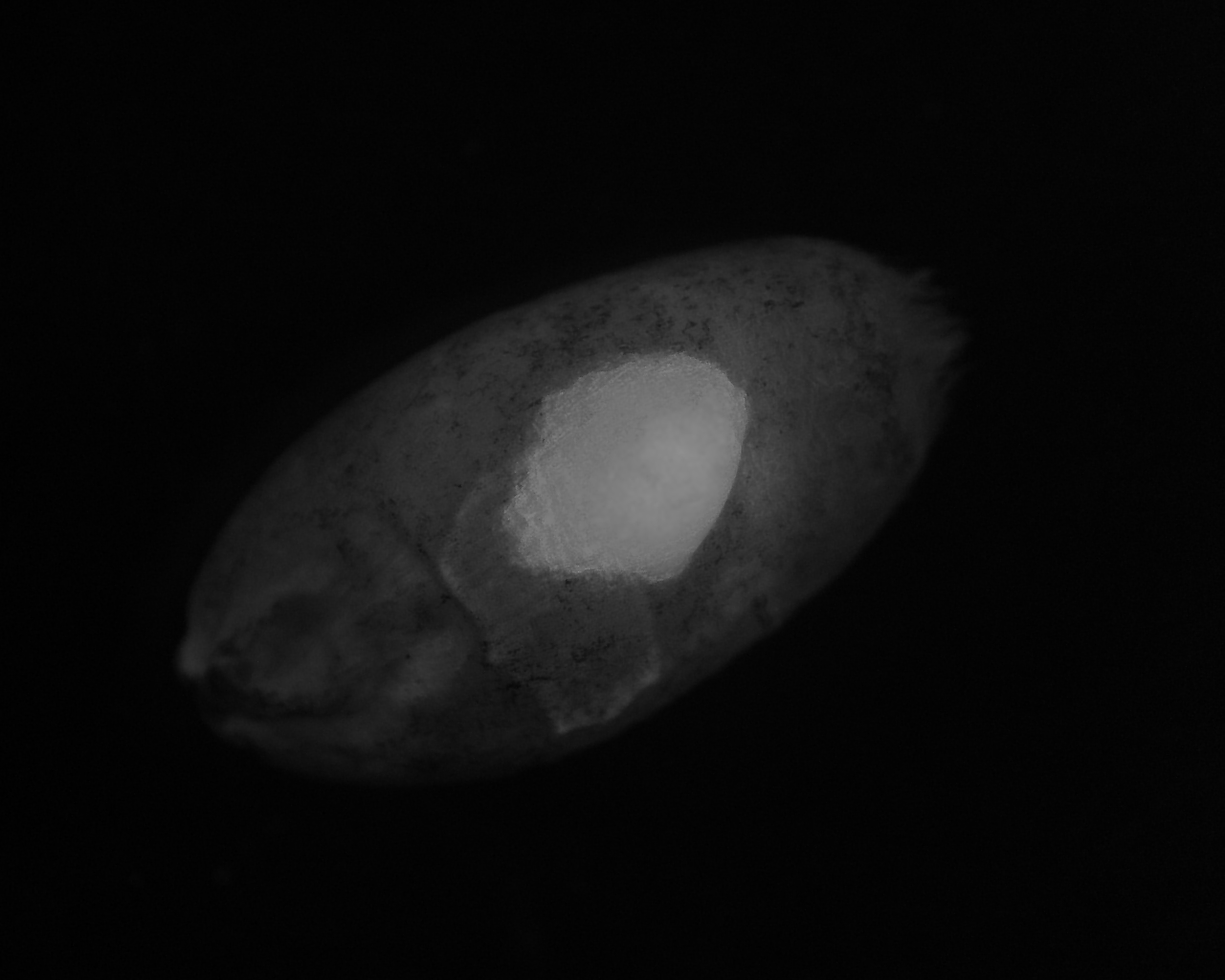

Supplement: S3 File — (ZIP) [file pone.0334274.s003.zip › D55 2.tif]

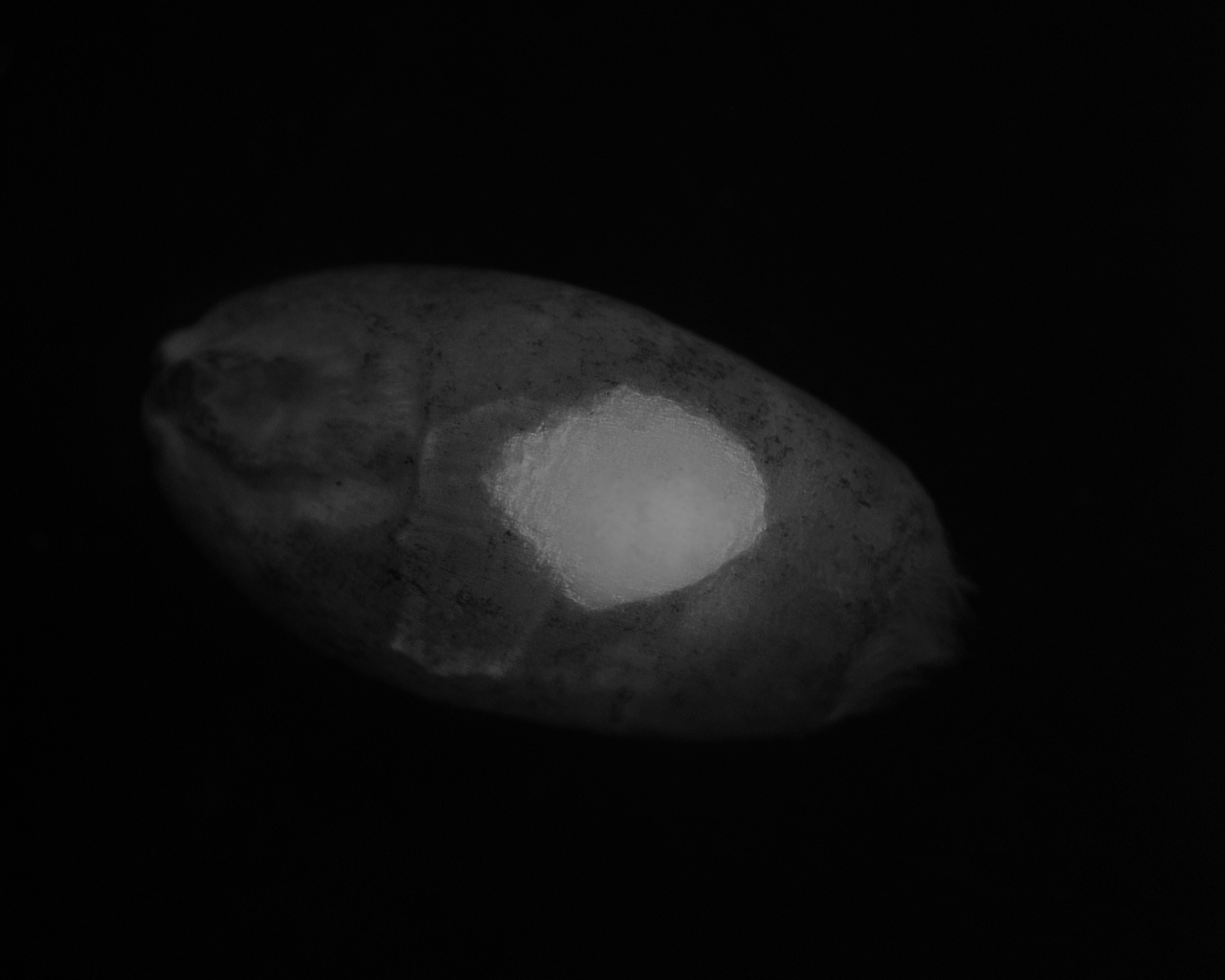

Supplement: S3 File — (ZIP) [file pone.0334274.s003.zip › D55 3.tif]

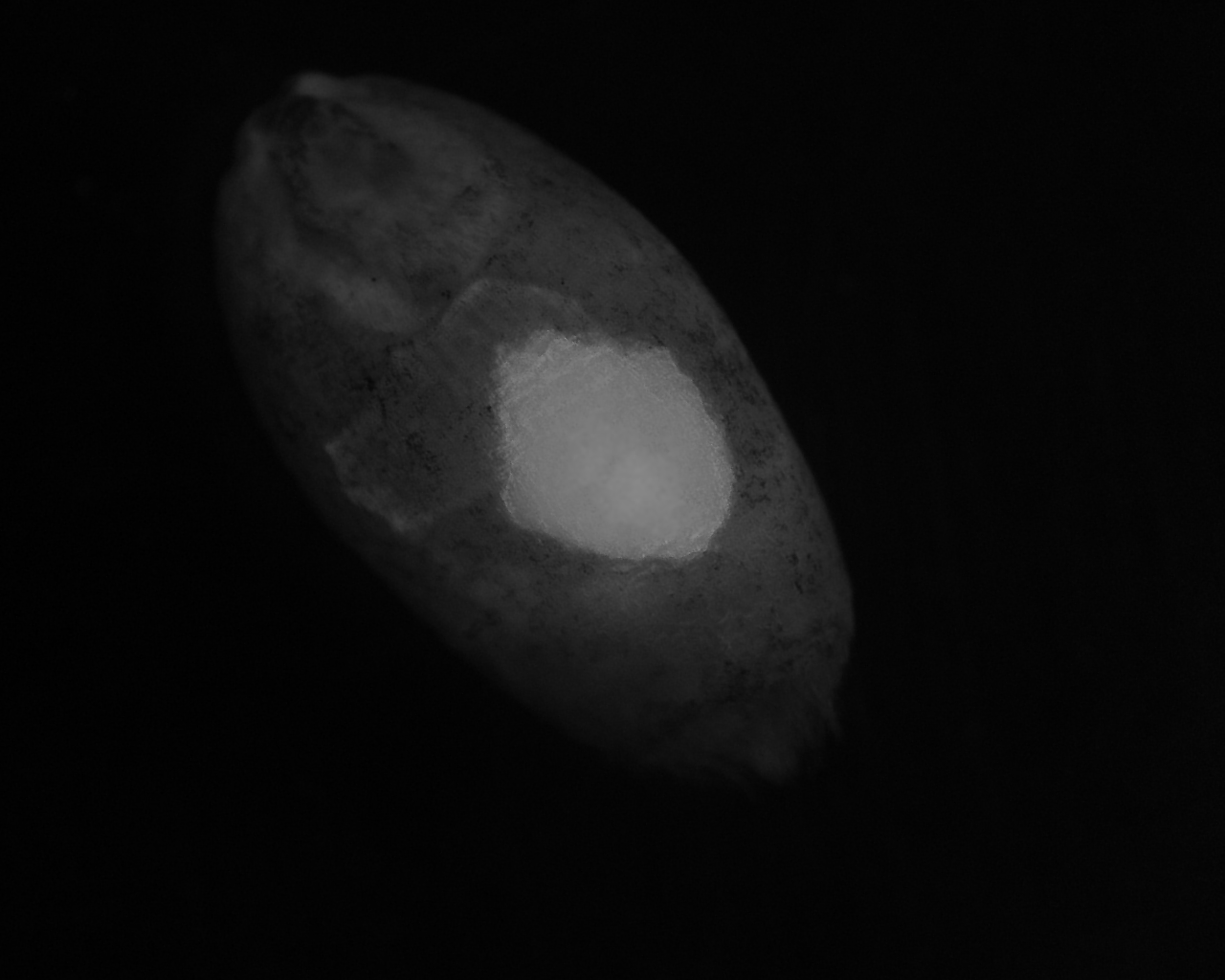

Supplement: S3 File — (ZIP) [file pone.0334274.s003.zip › D55 4.tif]

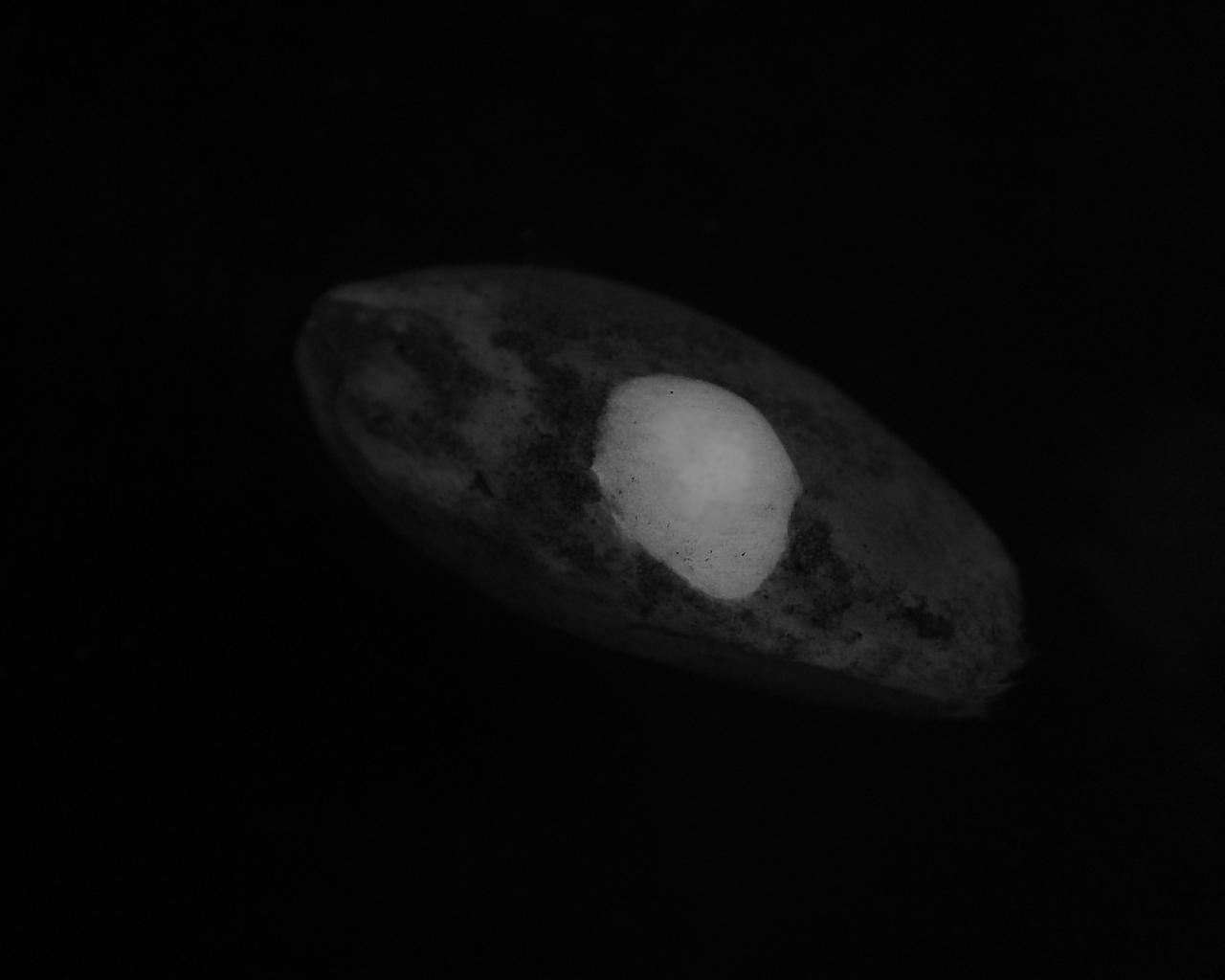

Supplement: S3 File — (ZIP) [file pone.0334274.s003.zip › LD00 1.tif]

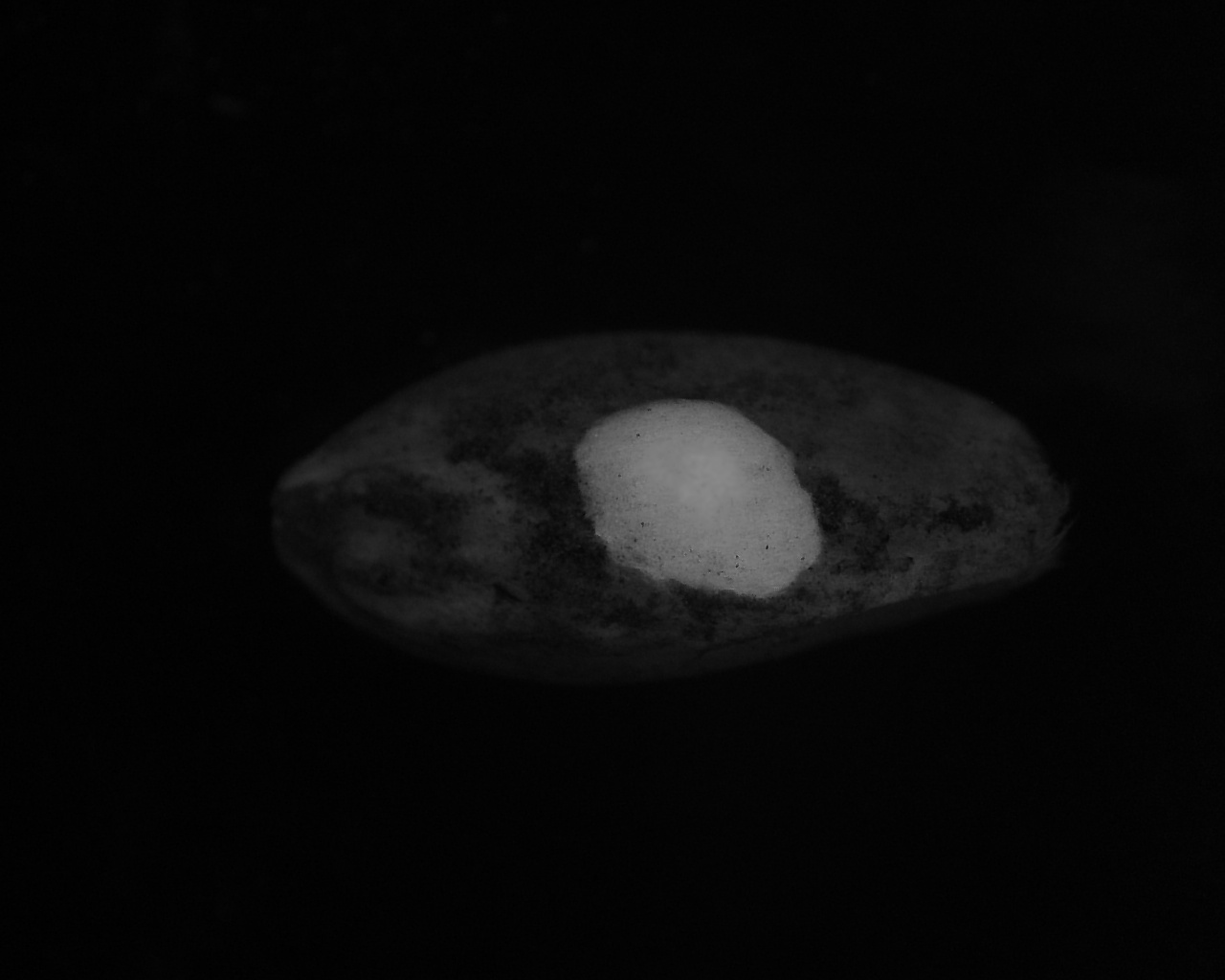

Supplement: S3 File — (ZIP) [file pone.0334274.s003.zip › LD00 2.tif]

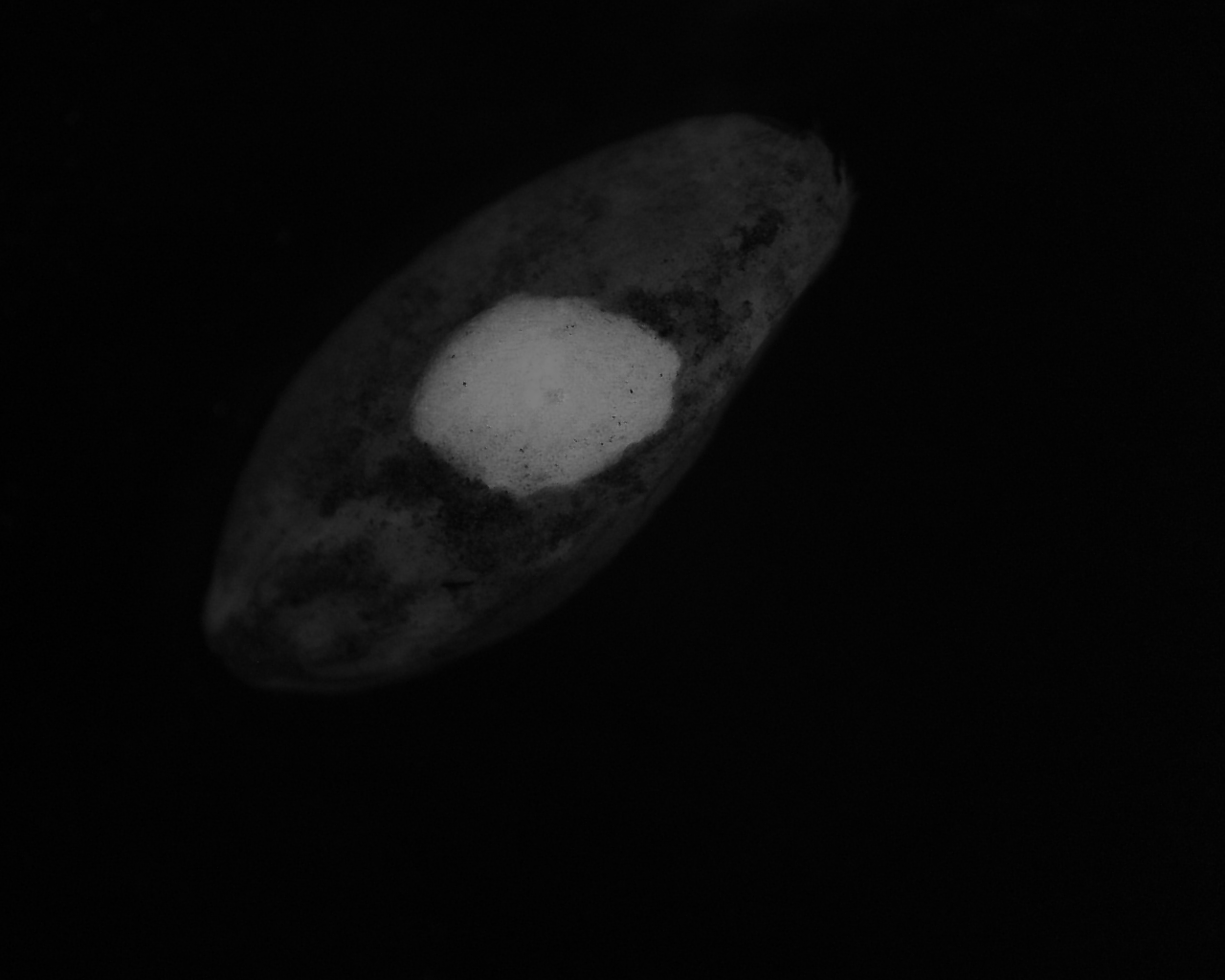

Supplement: S3 File — (ZIP) [file pone.0334274.s003.zip › LD00 3.tif]

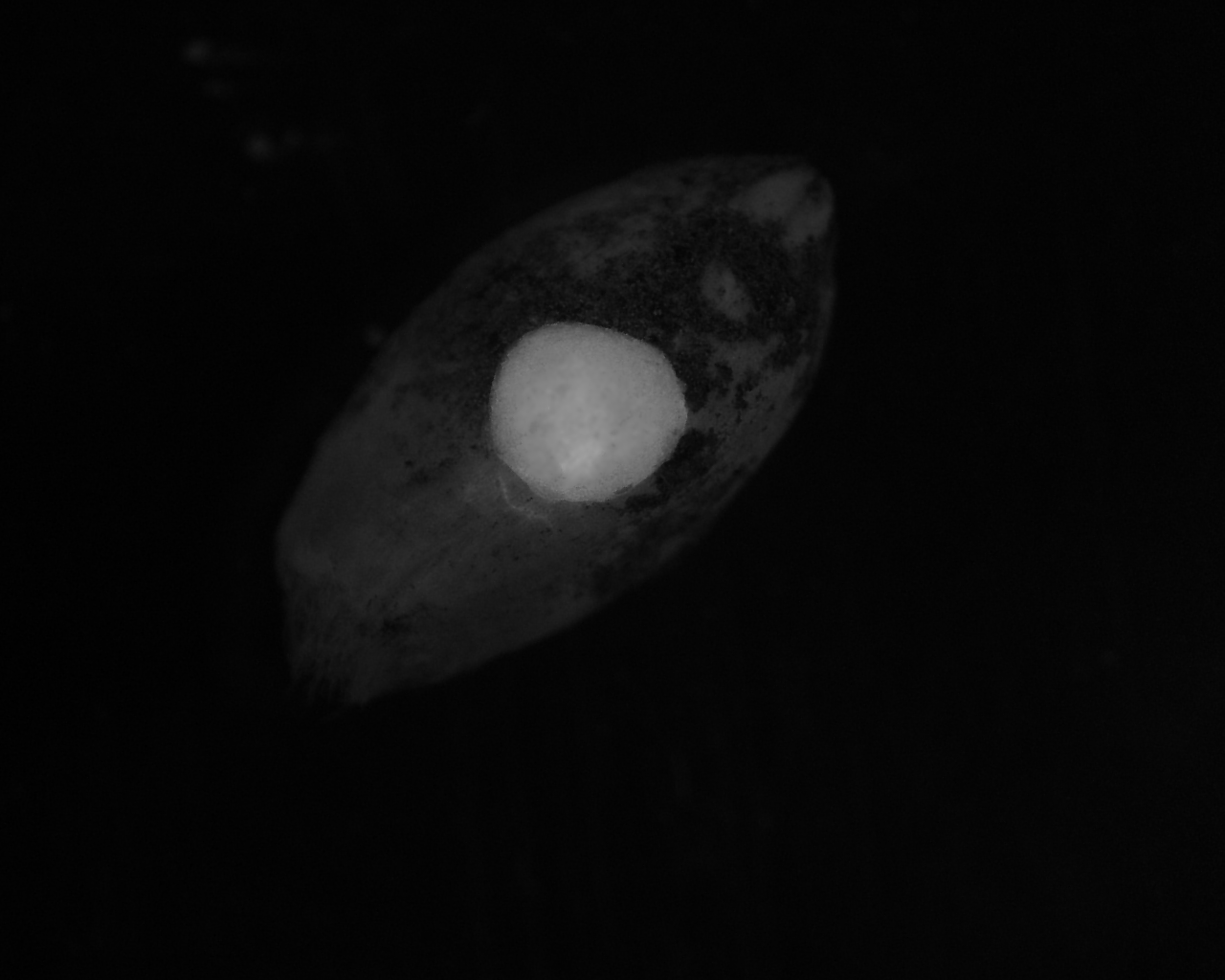

Supplement: S3 File — (ZIP) [file pone.0334274.s003.zip › LD22 1.tif]

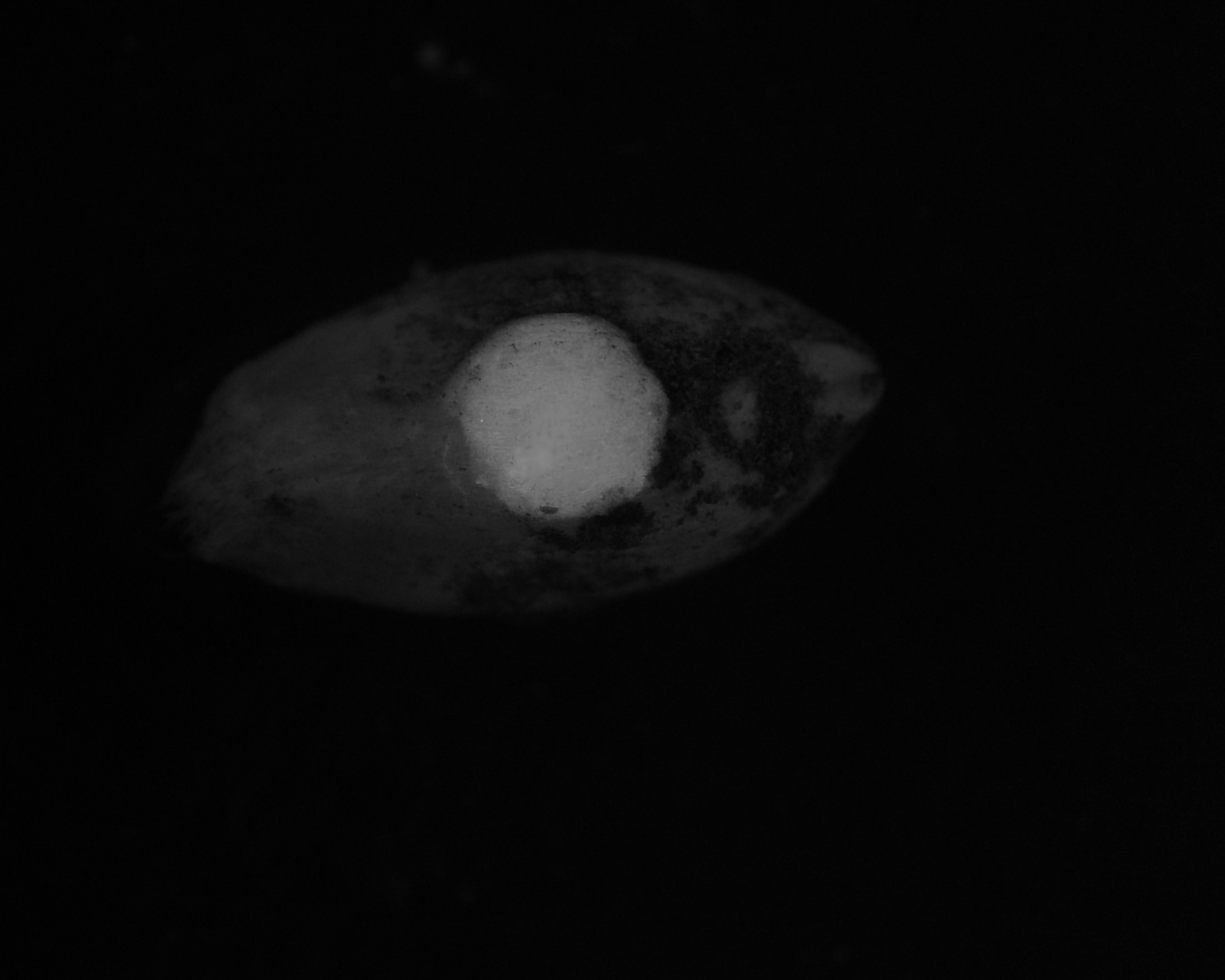

Supplement: S3 File — (ZIP) [file pone.0334274.s003.zip › LD22 2.tif]

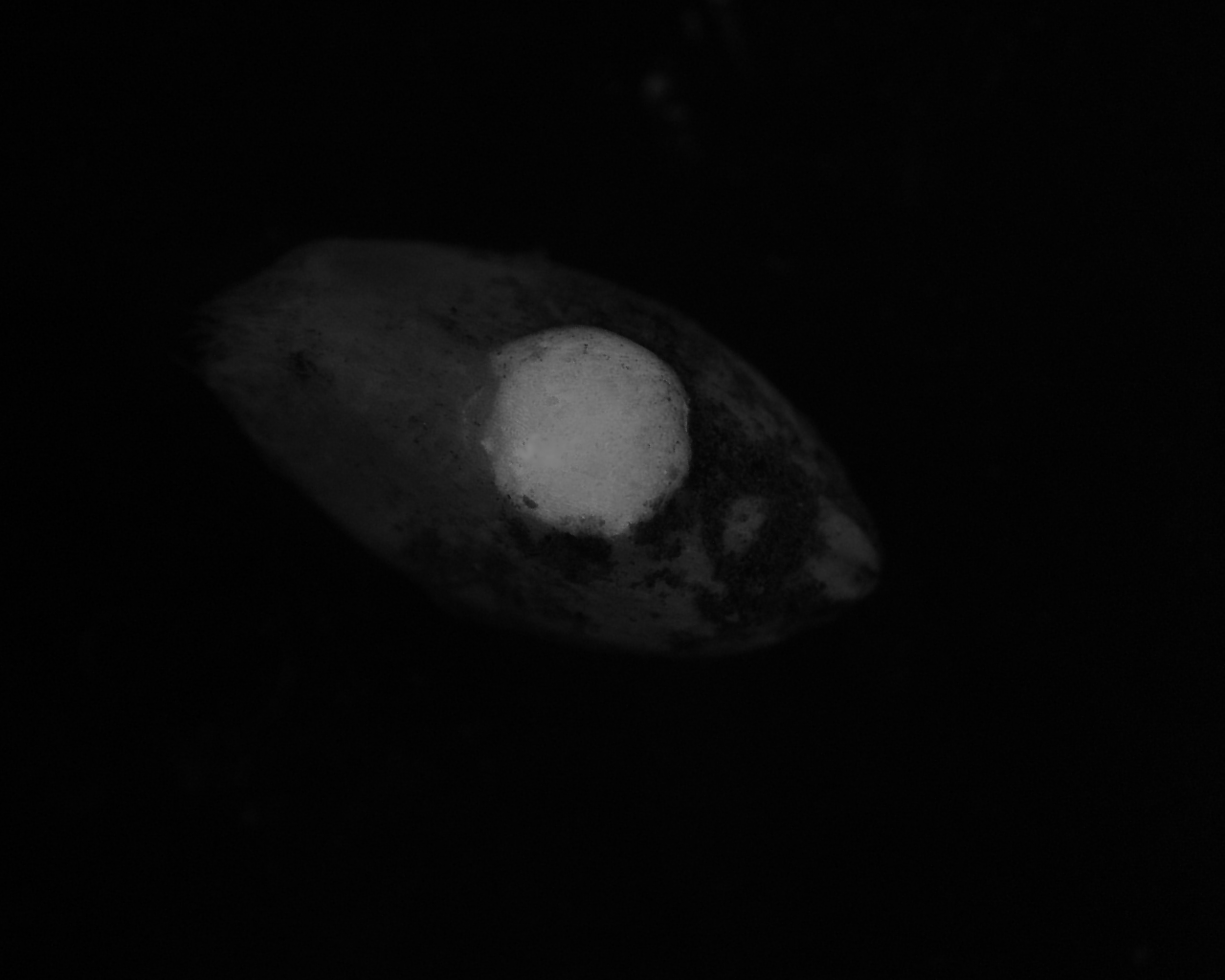

Supplement: S3 File — (ZIP) [file pone.0334274.s003.zip › LD22 3.tif]

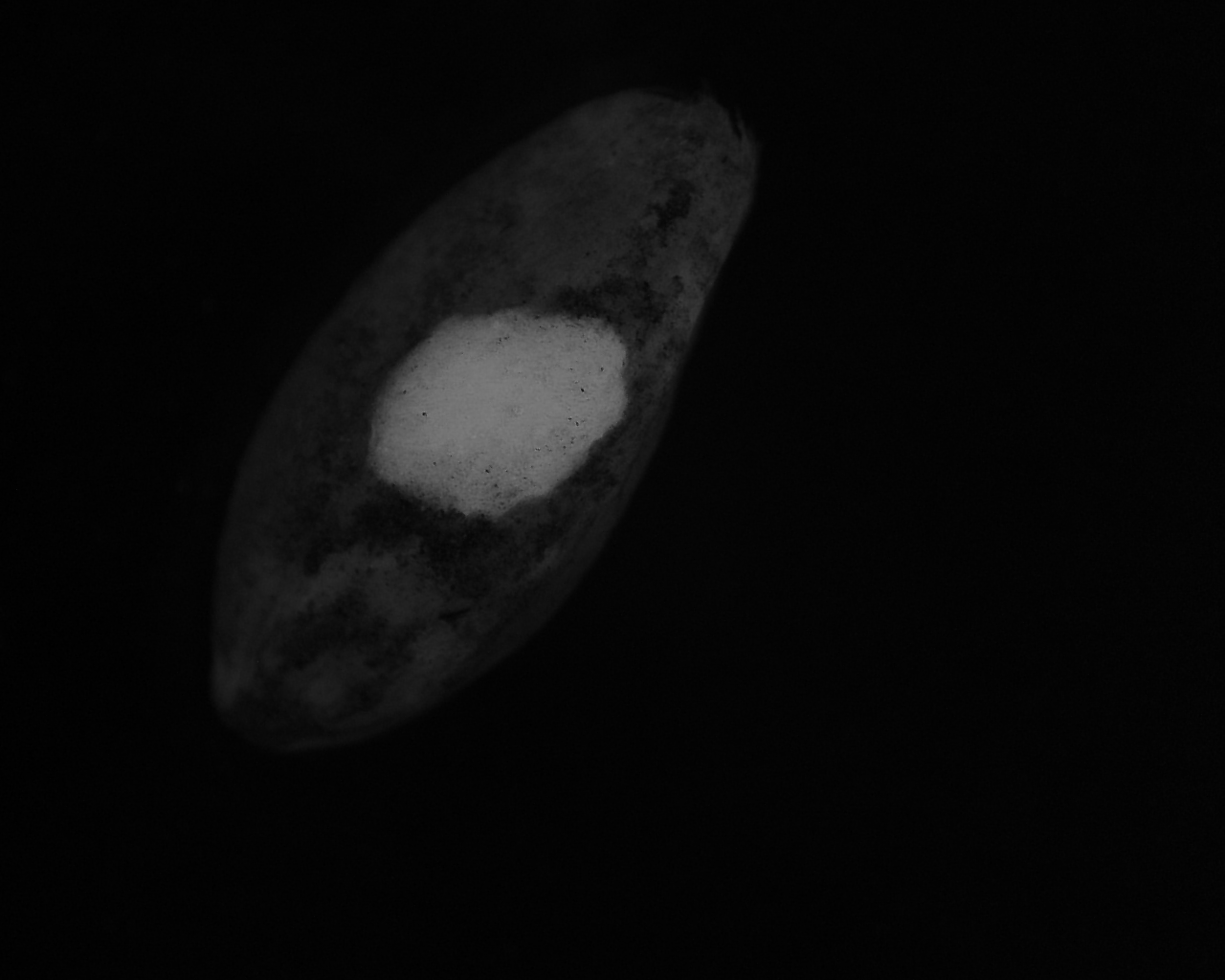

Supplement: S3 File — (ZIP) [file pone.0334274.s003.zip › LD22 4.tif]

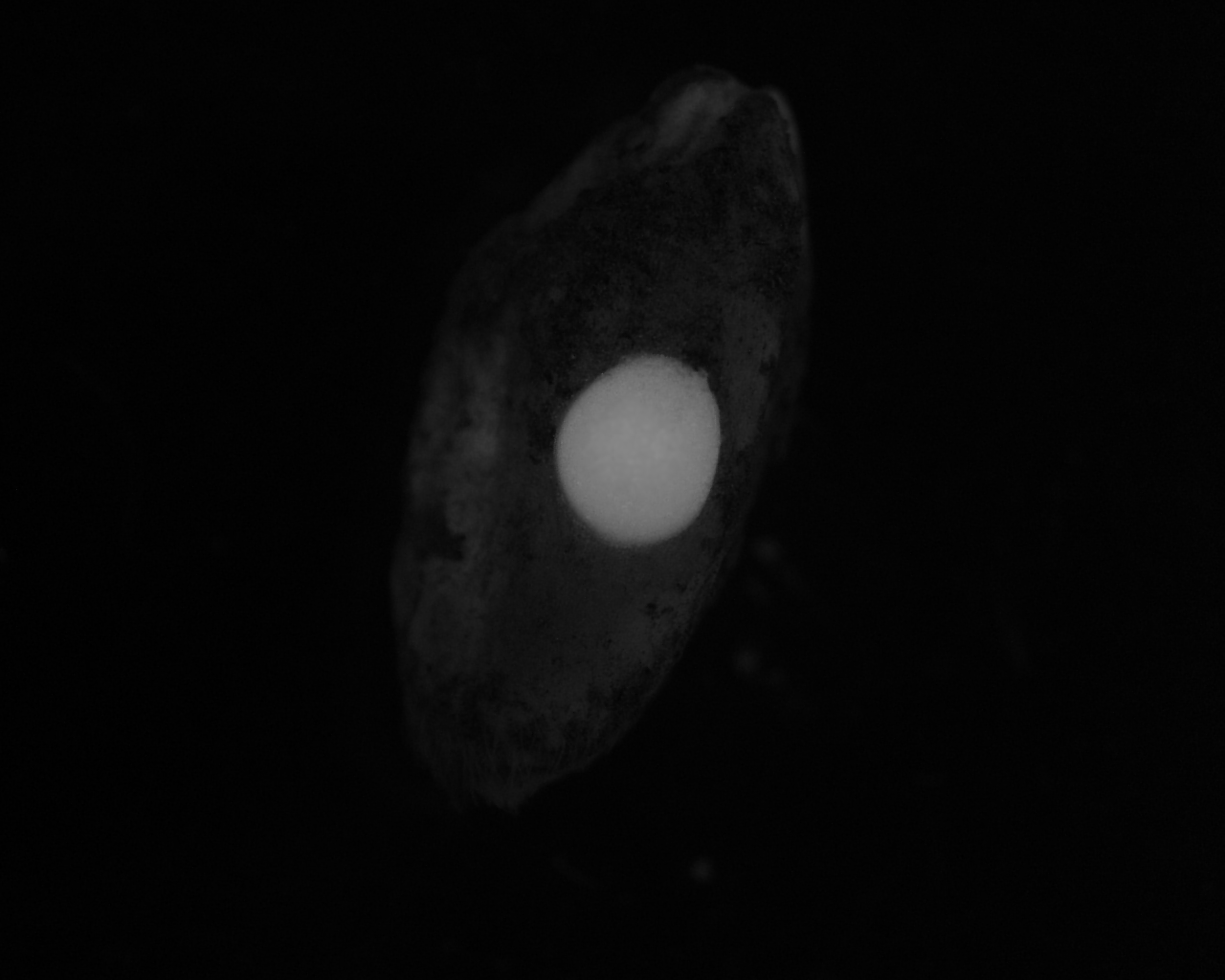

Supplement: S3 File — (ZIP) [file pone.0334274.s003.zip › LD33 1.tif]

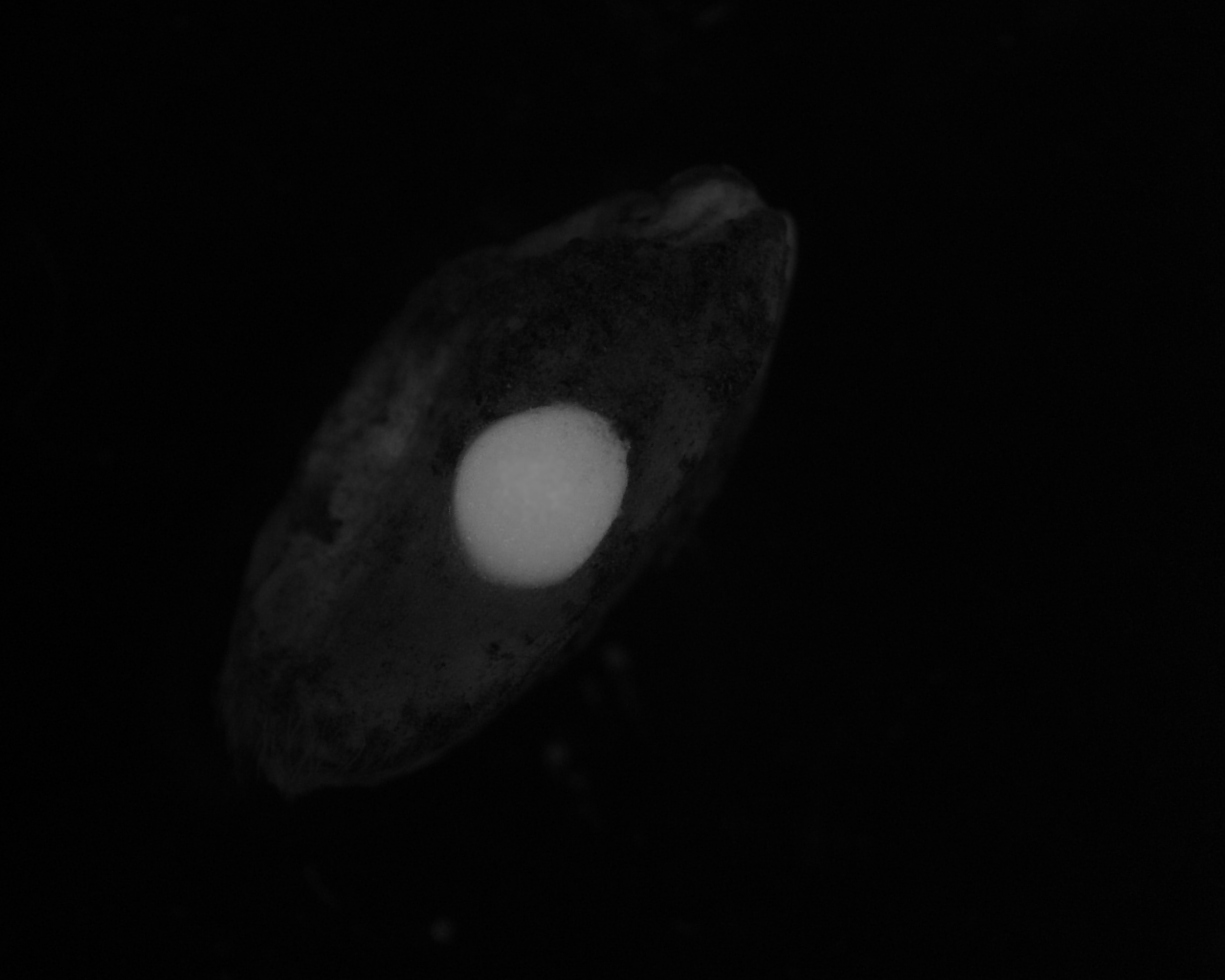

Supplement: S3 File — (ZIP) [file pone.0334274.s003.zip › LD33 2.tif]

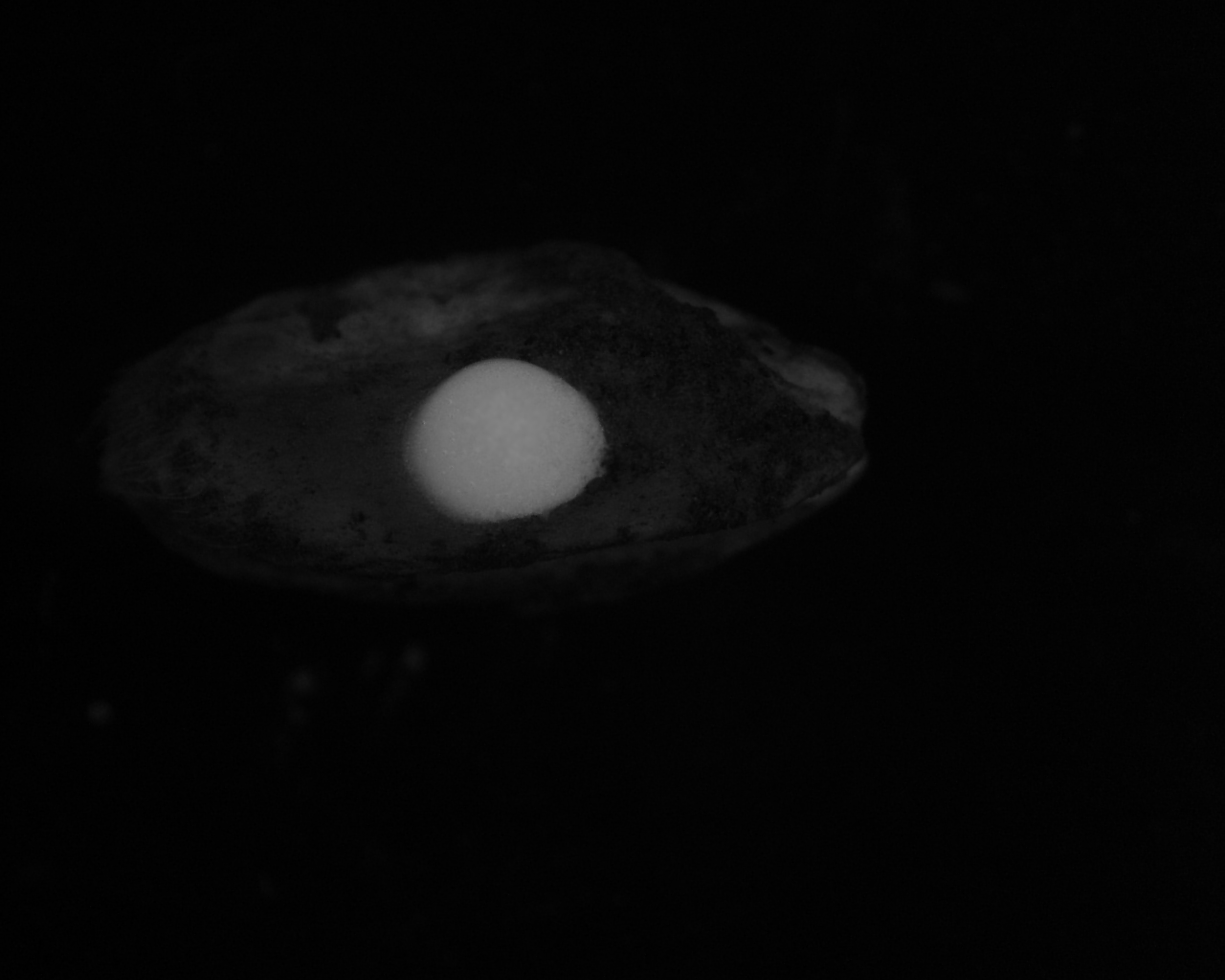

Supplement: S3 File — (ZIP) [file pone.0334274.s003.zip › LD33 3.tif]

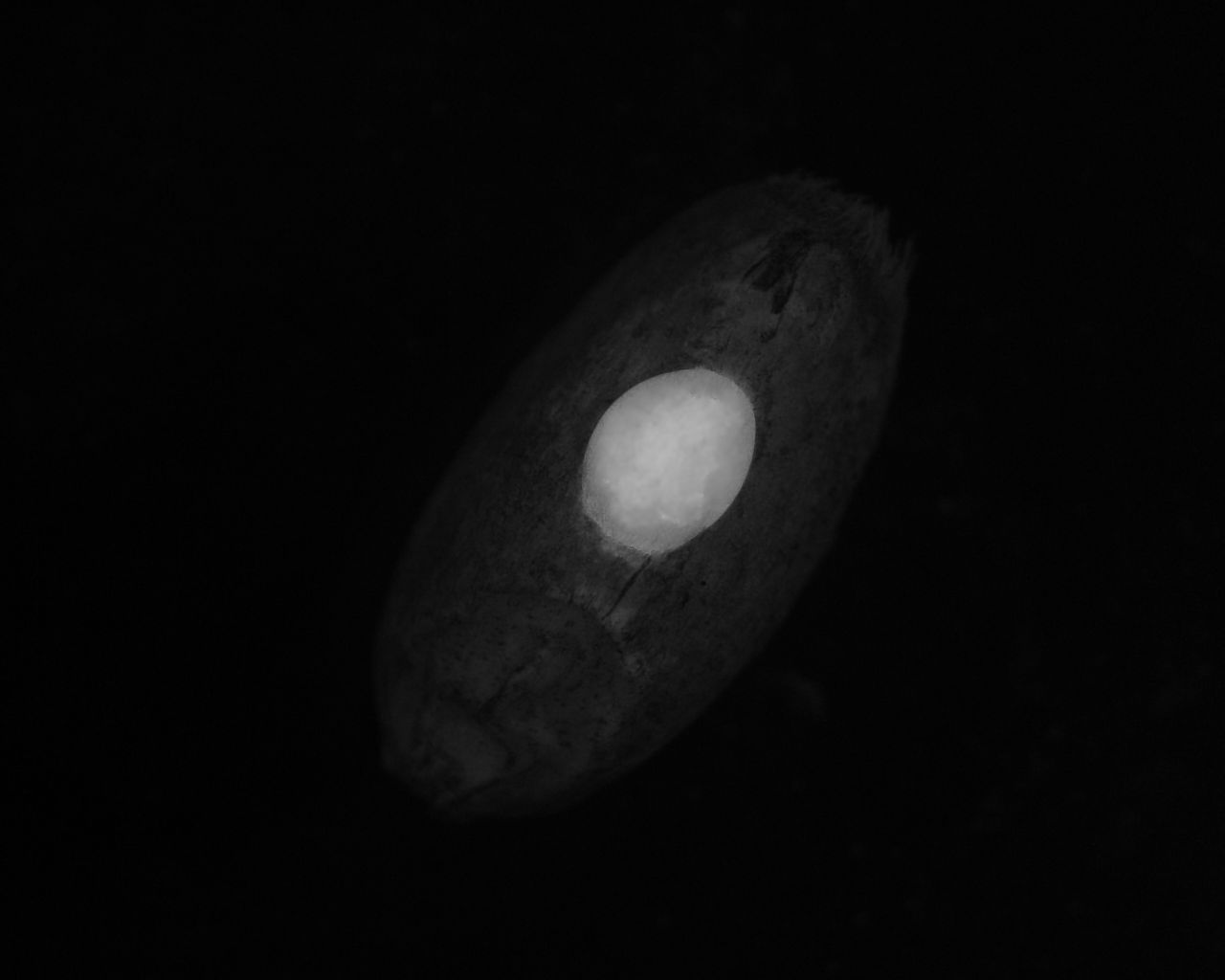

Supplement: S3 File — (ZIP) [file pone.0334274.s003.zip › LD44 1.tif]

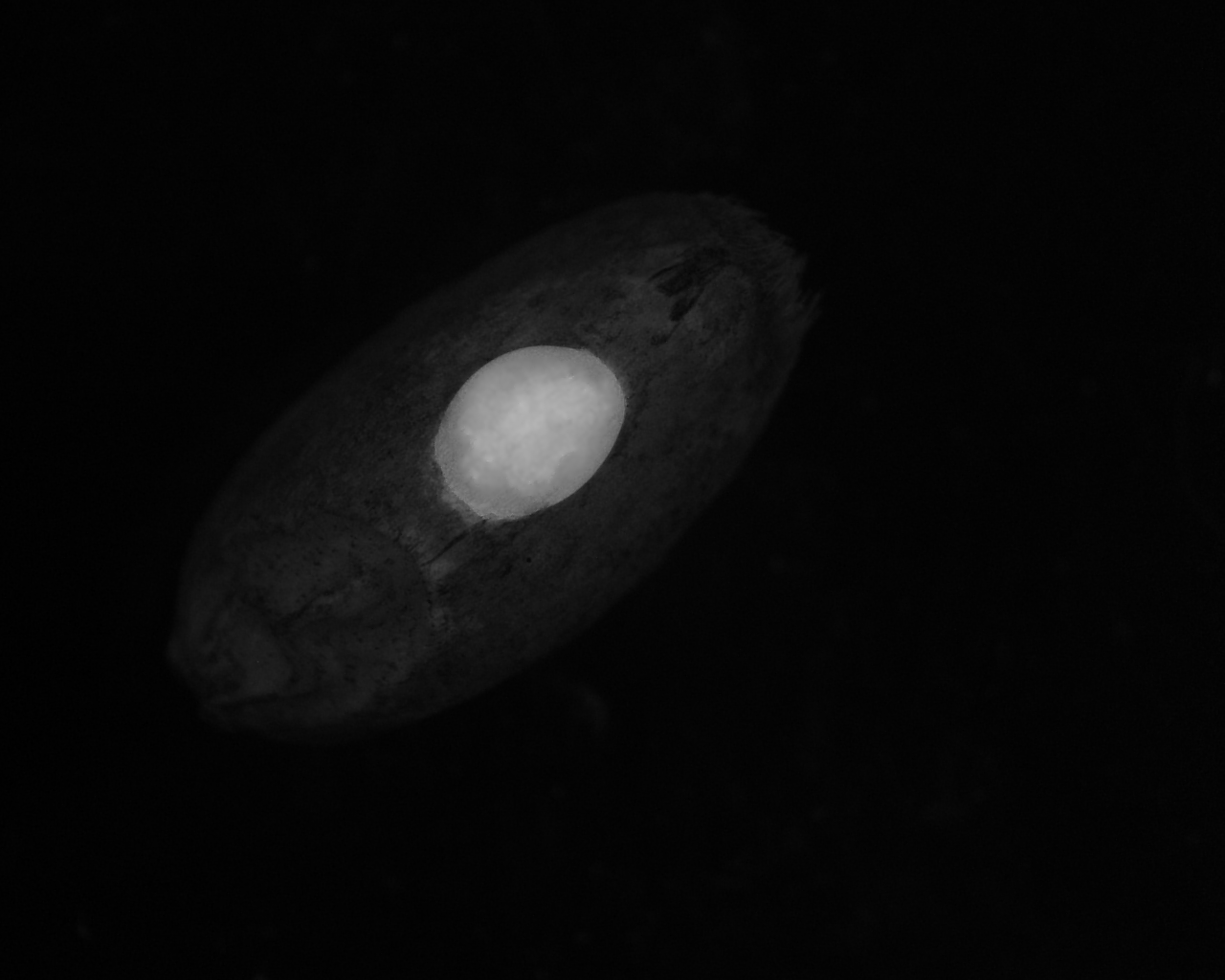

Supplement: S3 File — (ZIP) [file pone.0334274.s003.zip › LD44 2.tif]

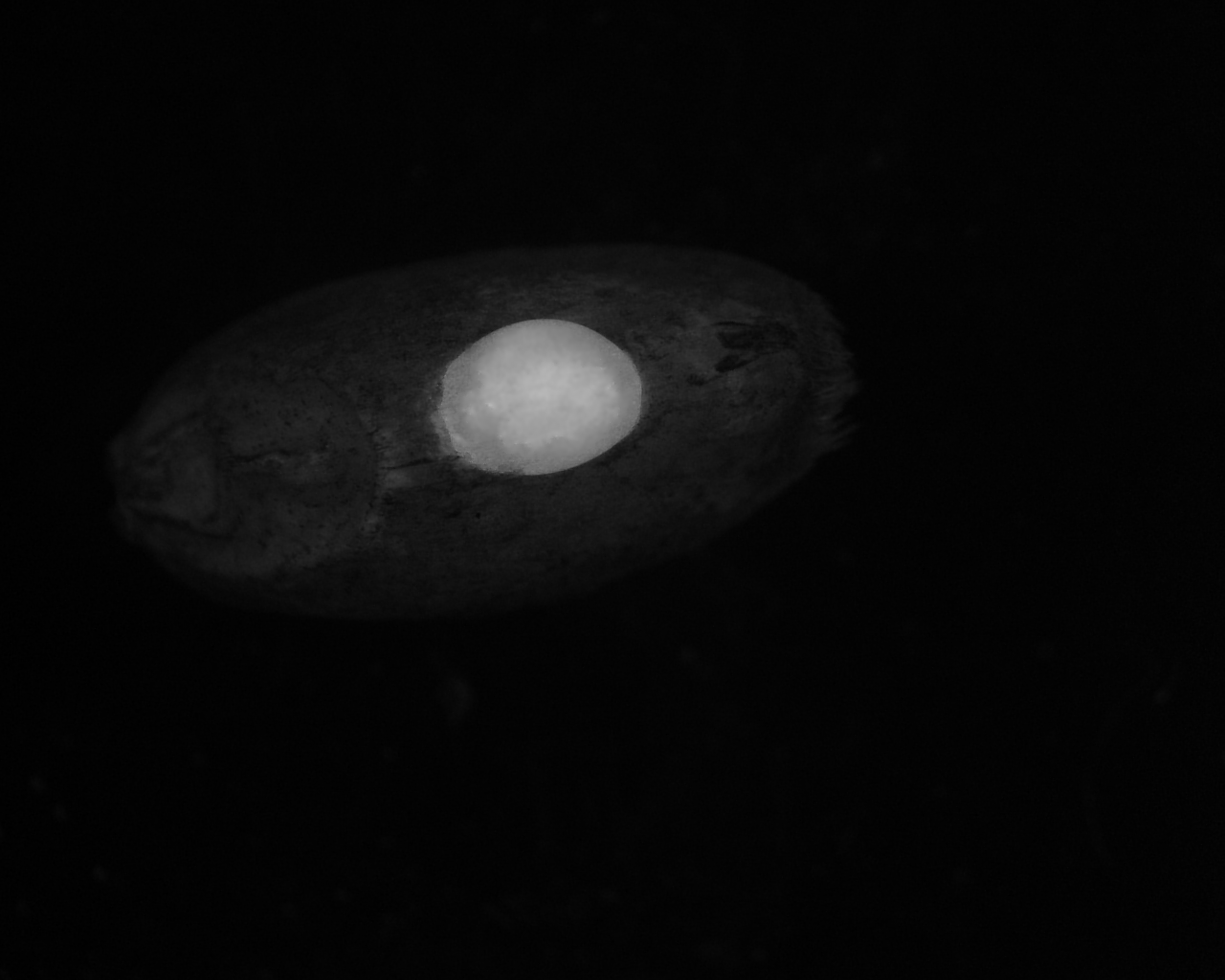

Supplement: S3 File — (ZIP) [file pone.0334274.s003.zip › LD44 3.tif]

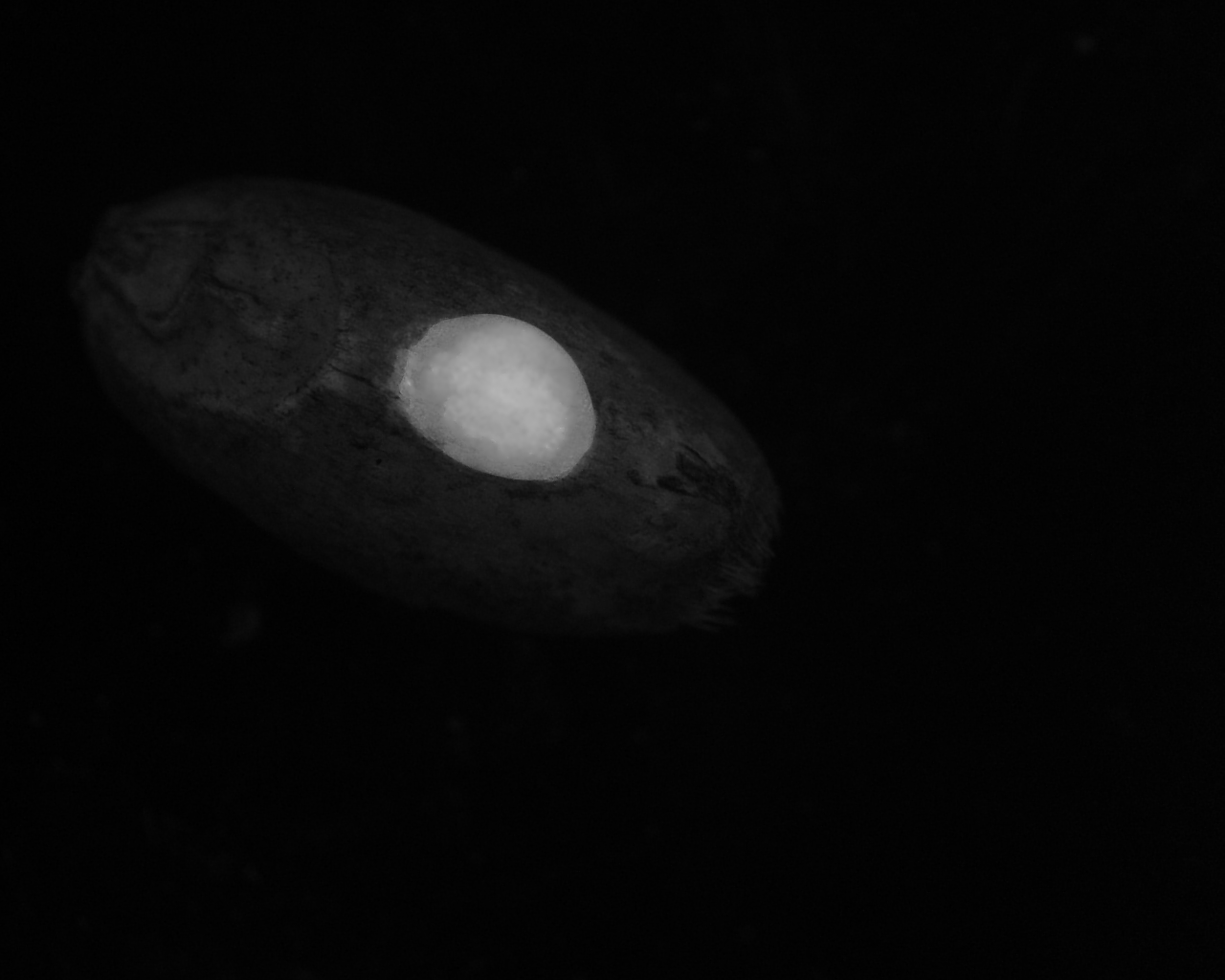

Supplement: S3 File — (ZIP) [file pone.0334274.s003.zip › LD44 4.tif]

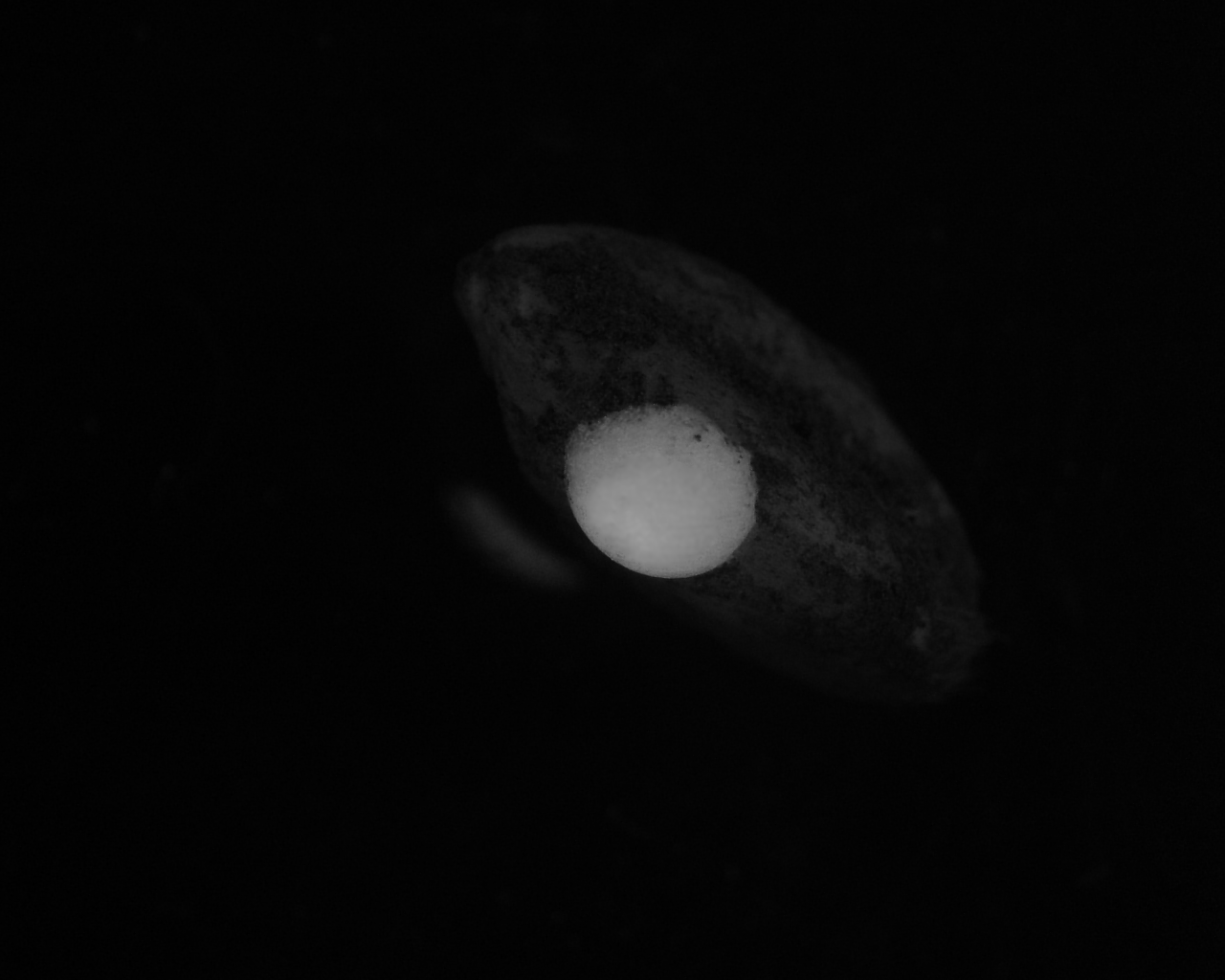

Supplement: S3 File — (ZIP) [file pone.0334274.s003.zip › LD55 1.tif]

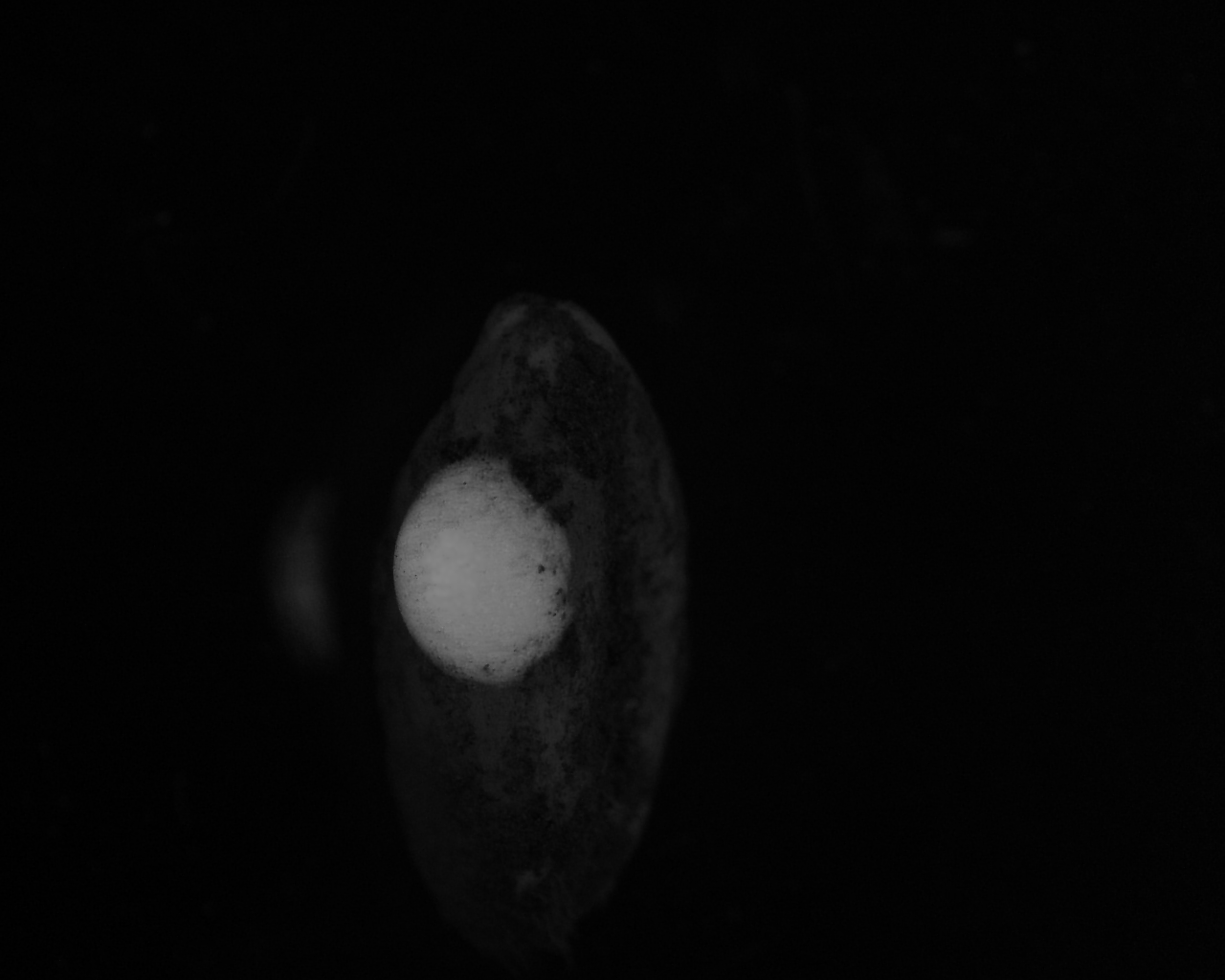

Supplement: S3 File — (ZIP) [file pone.0334274.s003.zip › LD55 2.tif]

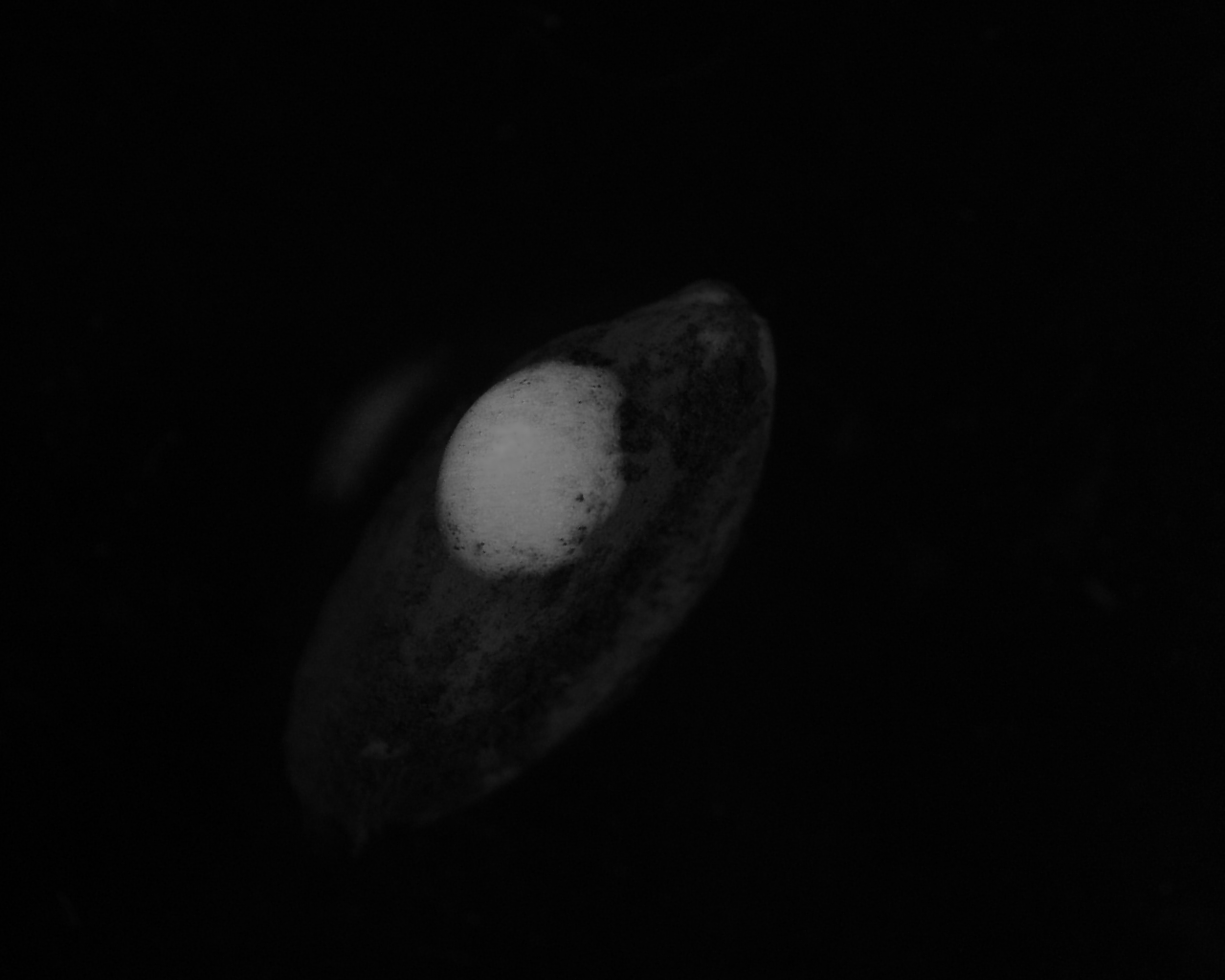

Supplement: S3 File — (ZIP) [file pone.0334274.s003.zip › LD55 3.tif]

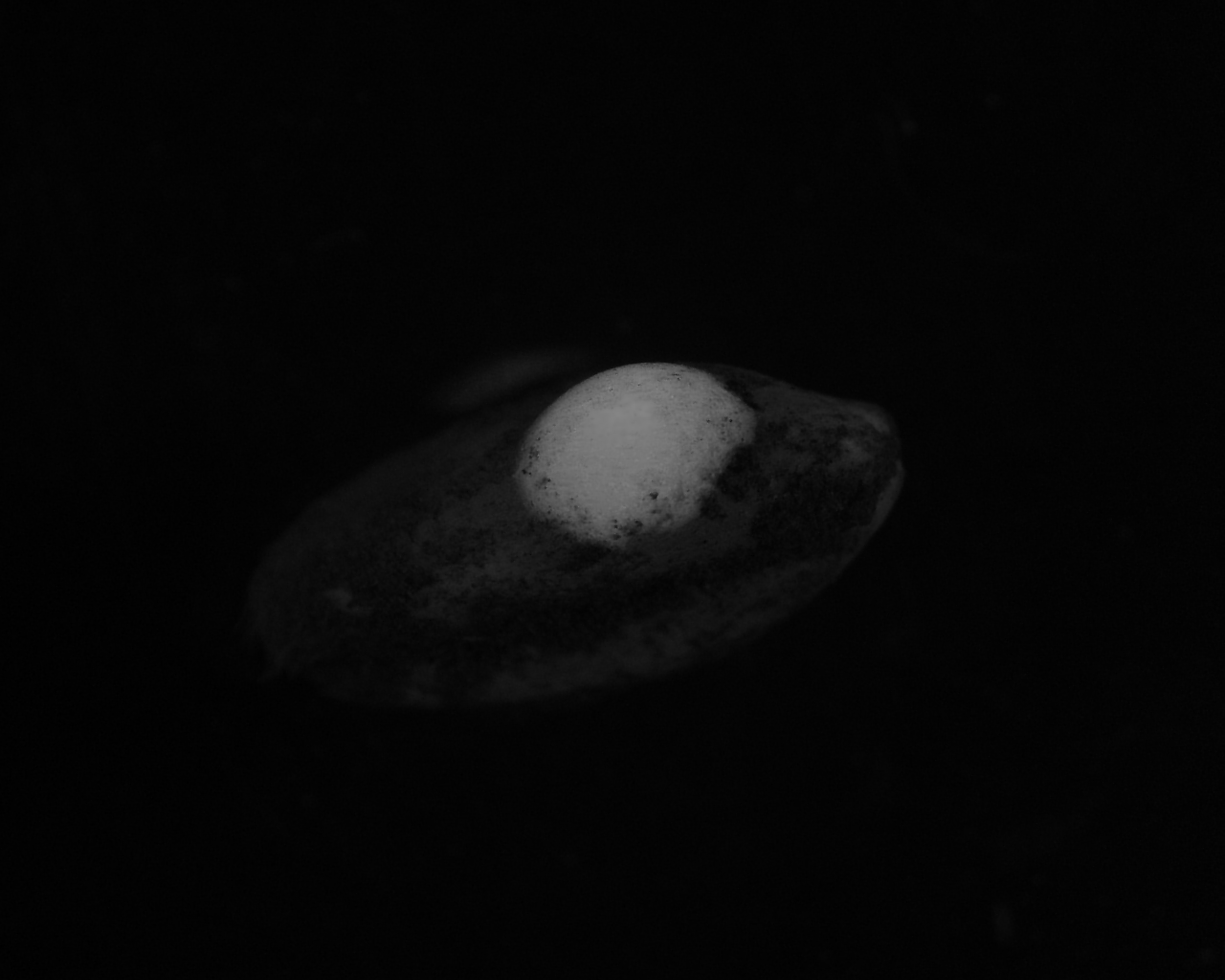

Supplement: S3 File — (ZIP) [file pone.0334274.s003.zip › LD55 4.tif]

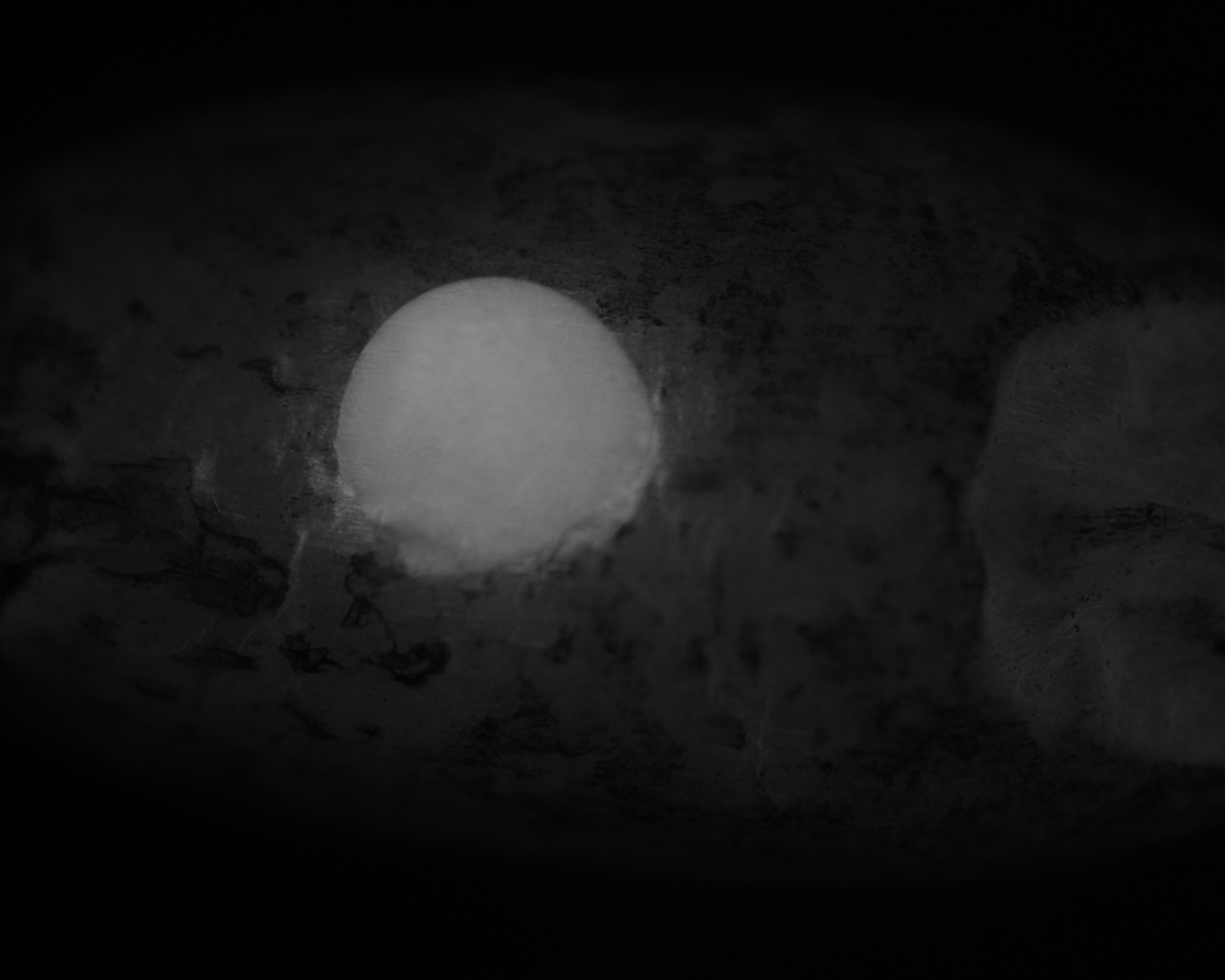

Supplement: S4 File — (ZIP) [file pone.0334274.s004.zip › GezawyDctrl 1.tif]

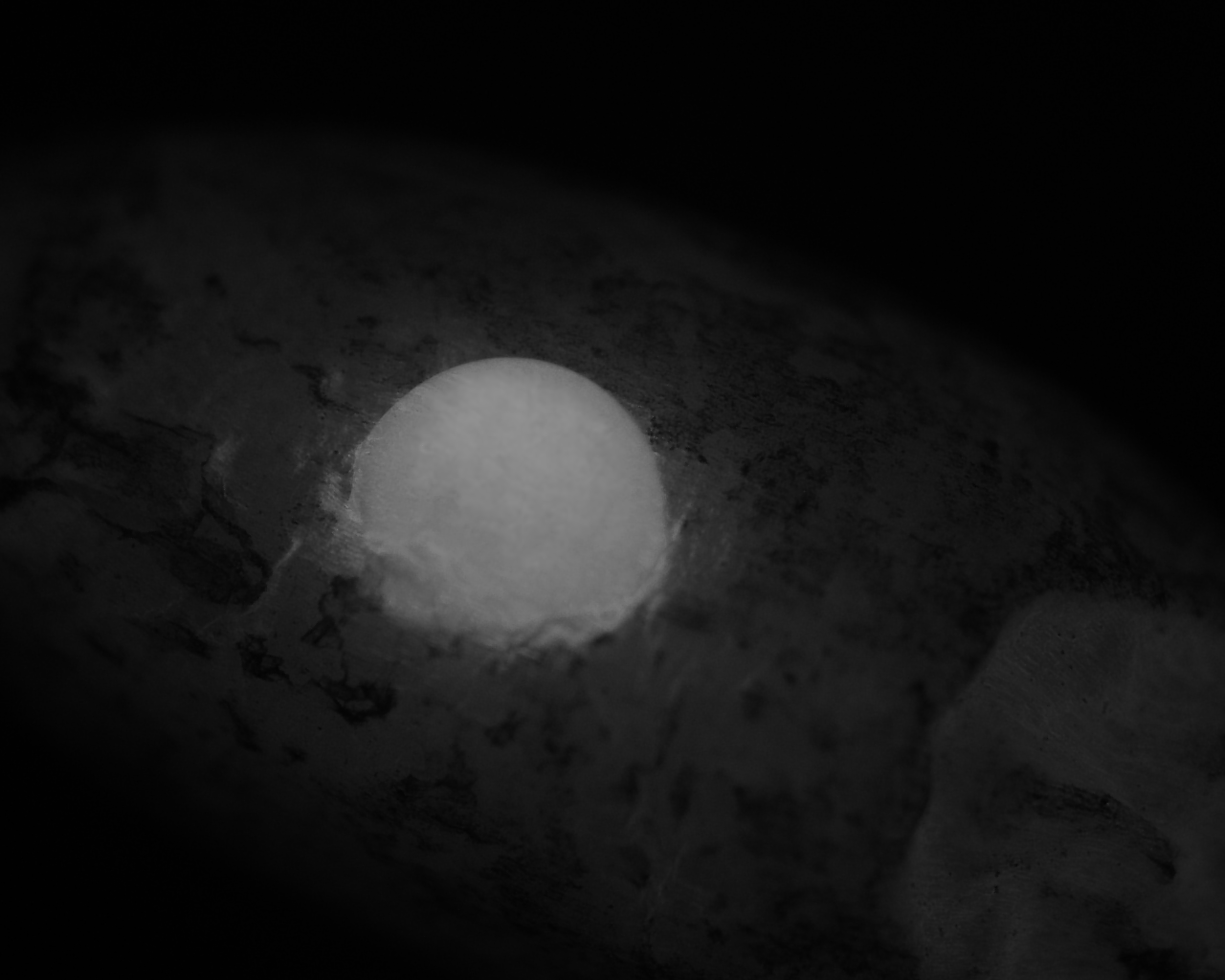

Supplement: S4 File — (ZIP) [file pone.0334274.s004.zip › GezawyDctrl 2.tif]

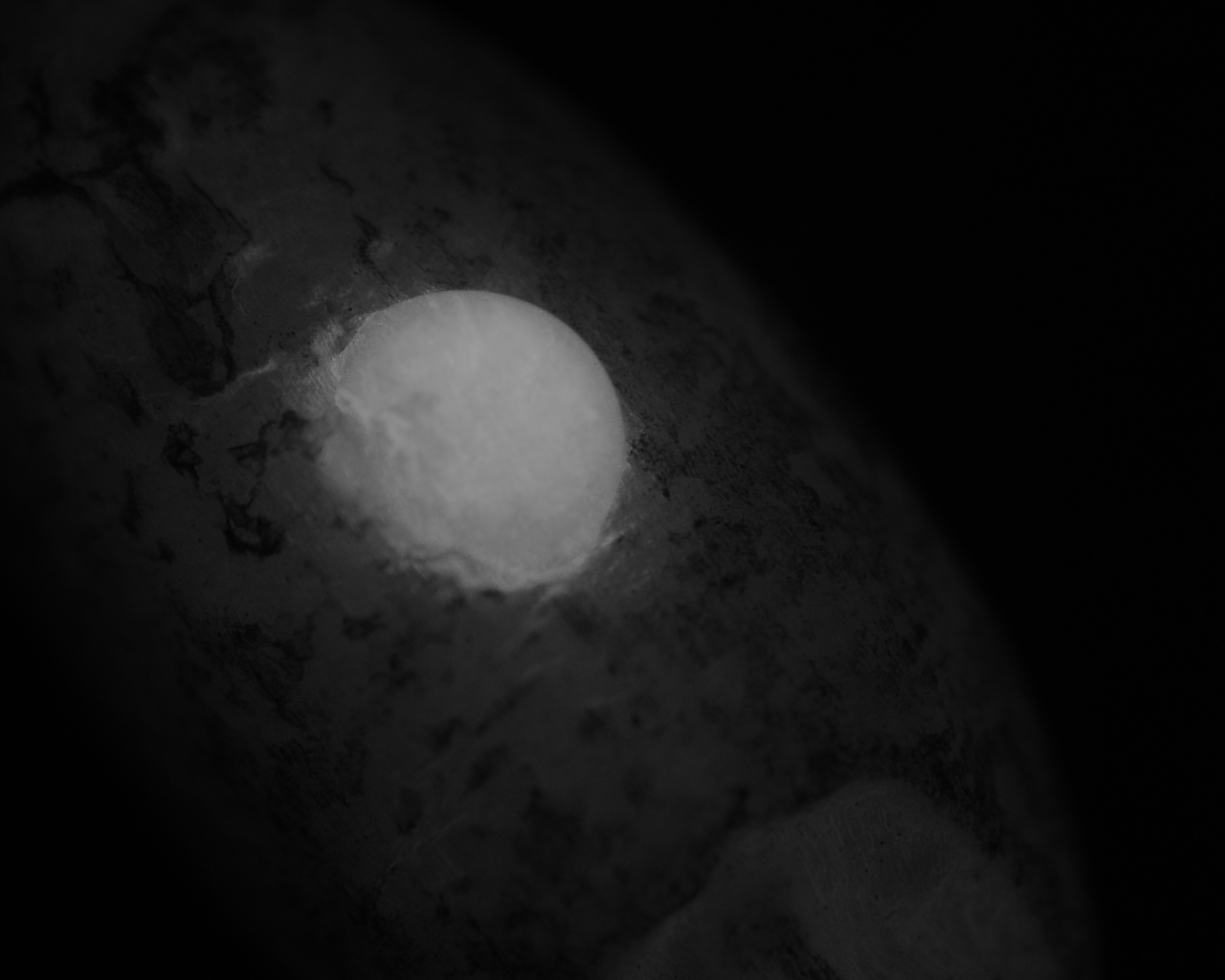

Supplement: S4 File — (ZIP) [file pone.0334274.s004.zip › GezawyDctrl 3.tif]

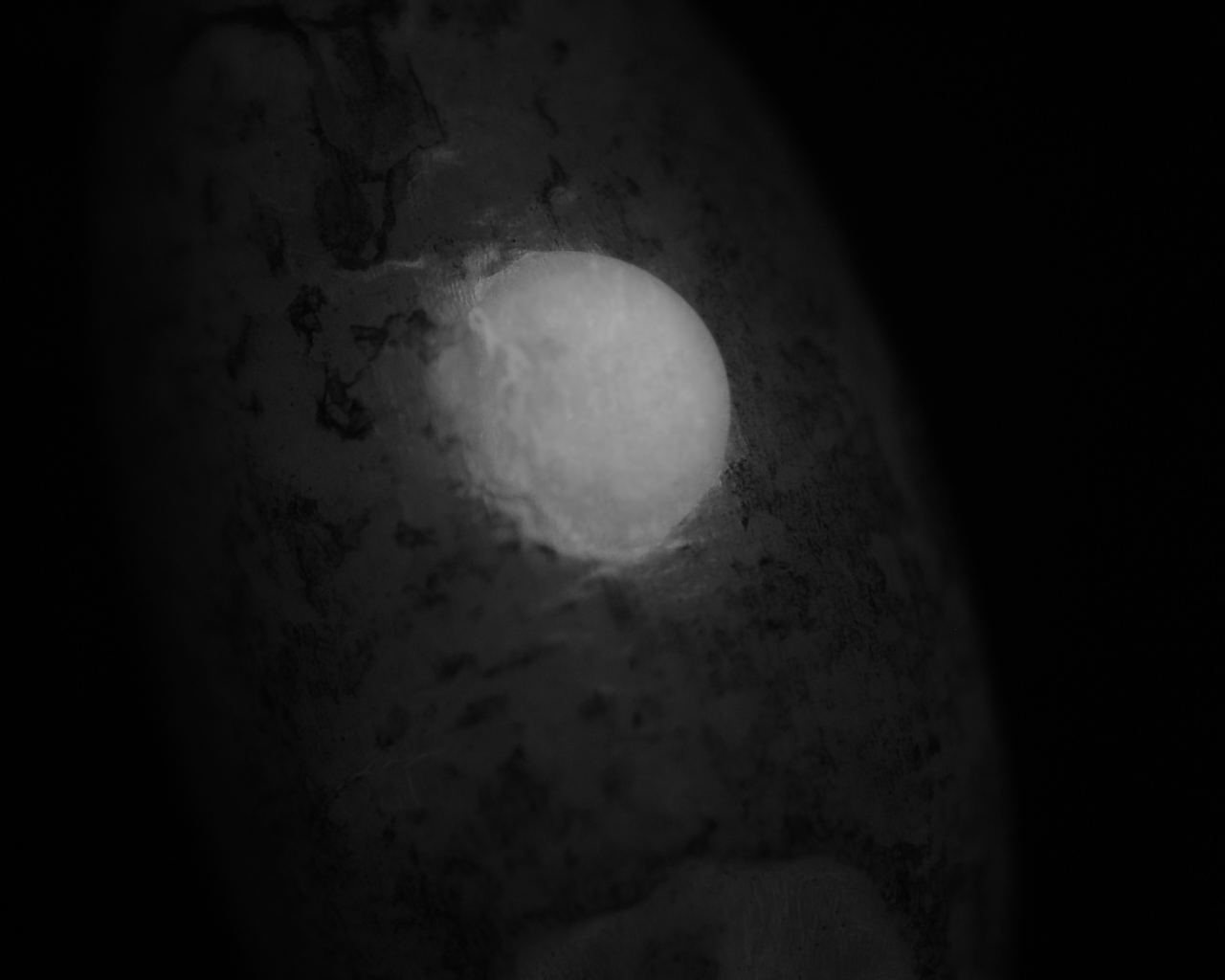

Supplement: S4 File — (ZIP) [file pone.0334274.s004.zip › GezawyDctrl 4.tif]

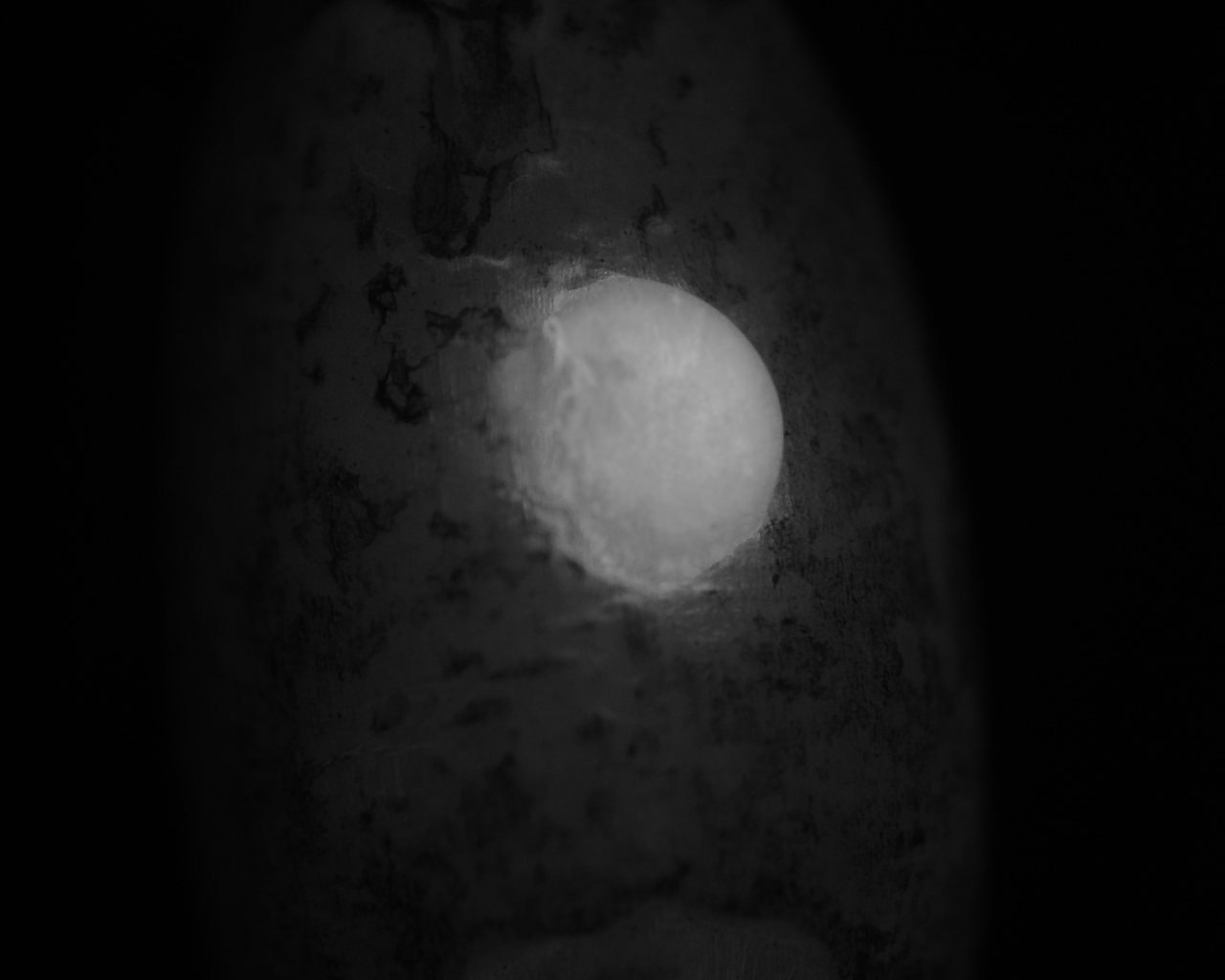

Supplement: S4 File — (ZIP) [file pone.0334274.s004.zip › GezawyDctrl 5.tif]

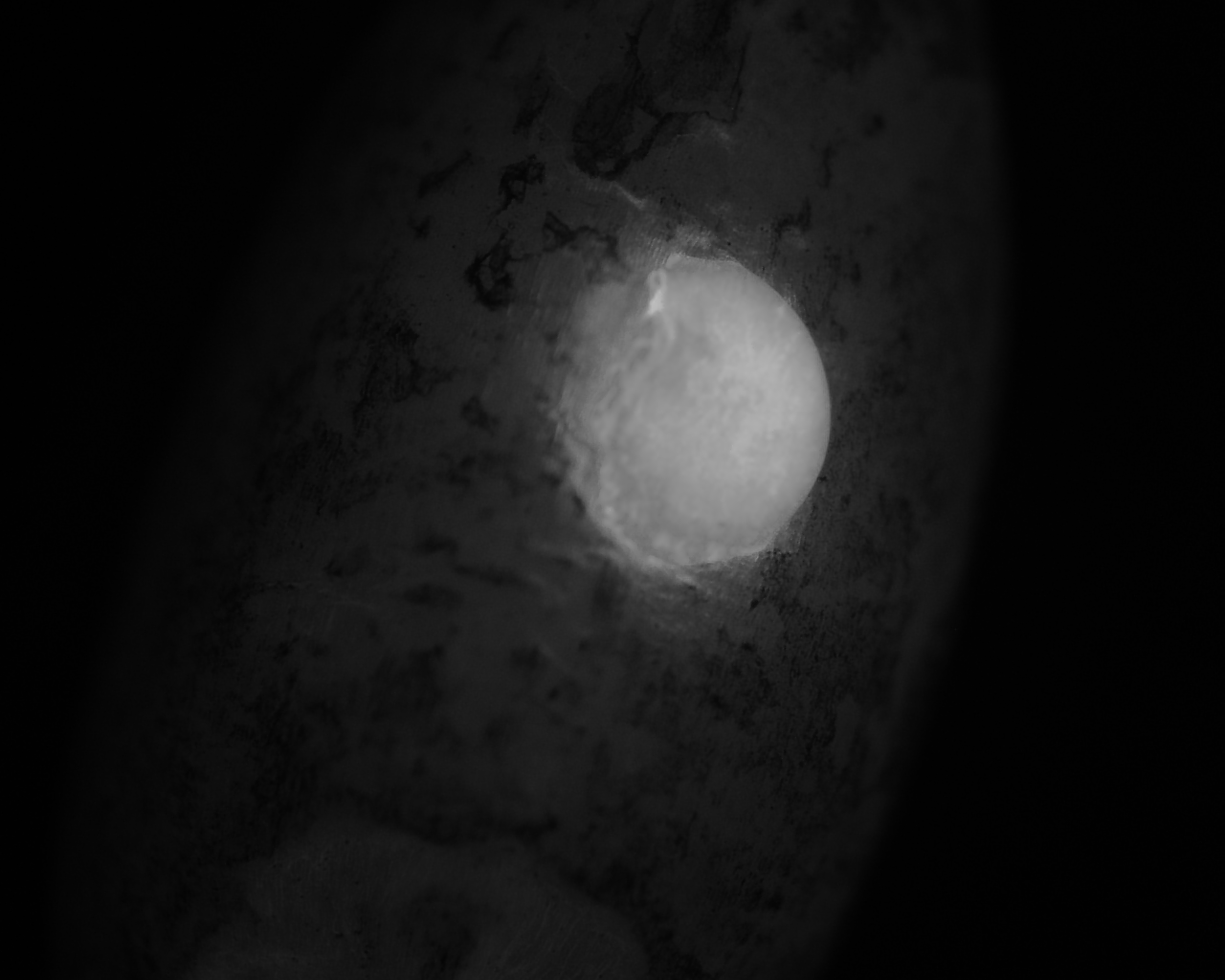

Supplement: S4 File — (ZIP) [file pone.0334274.s004.zip › GezawyDctrl 6.tif]

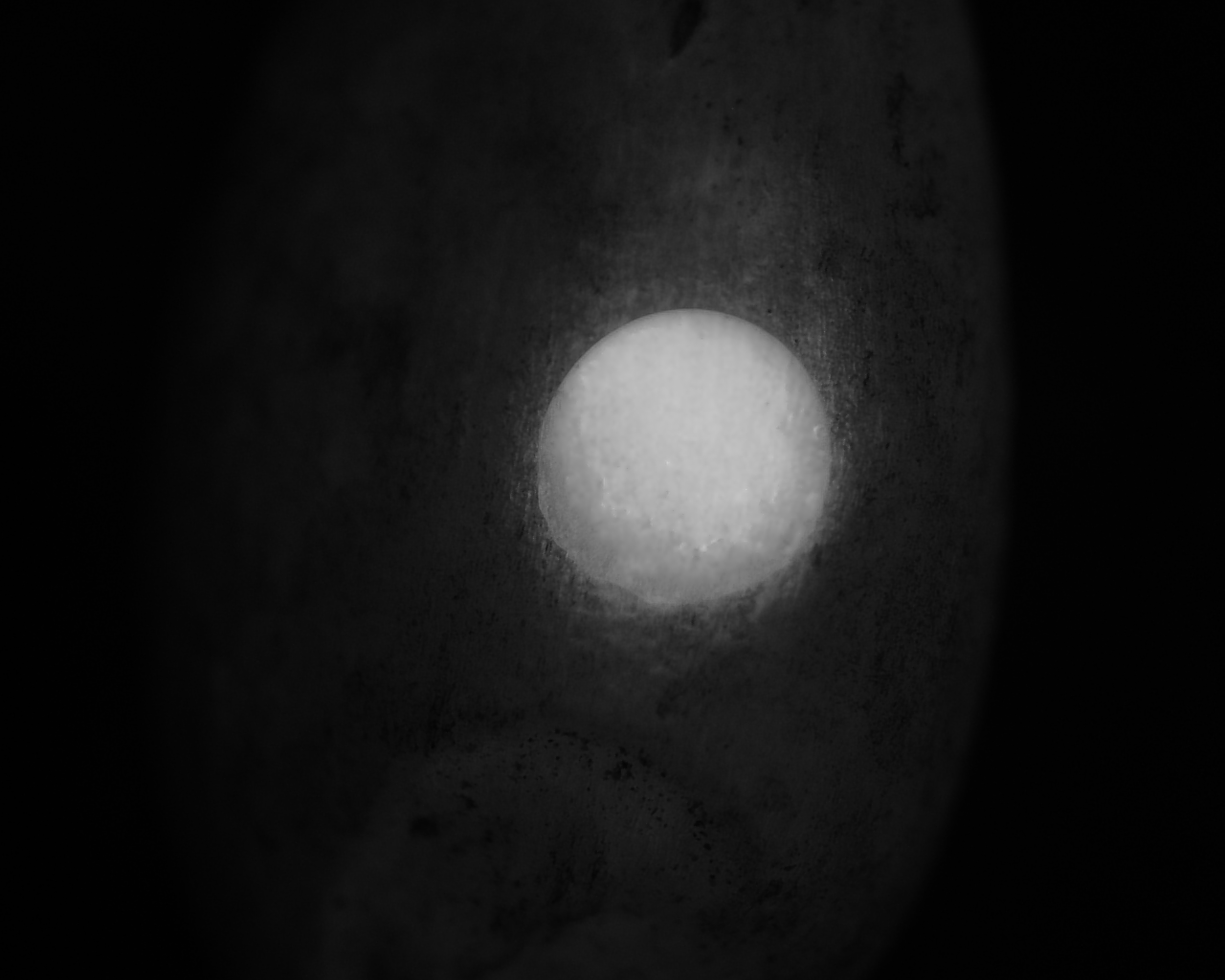

Supplement: S4 File — (ZIP) [file pone.0334274.s004.zip › GezawyDD00 1.tif]

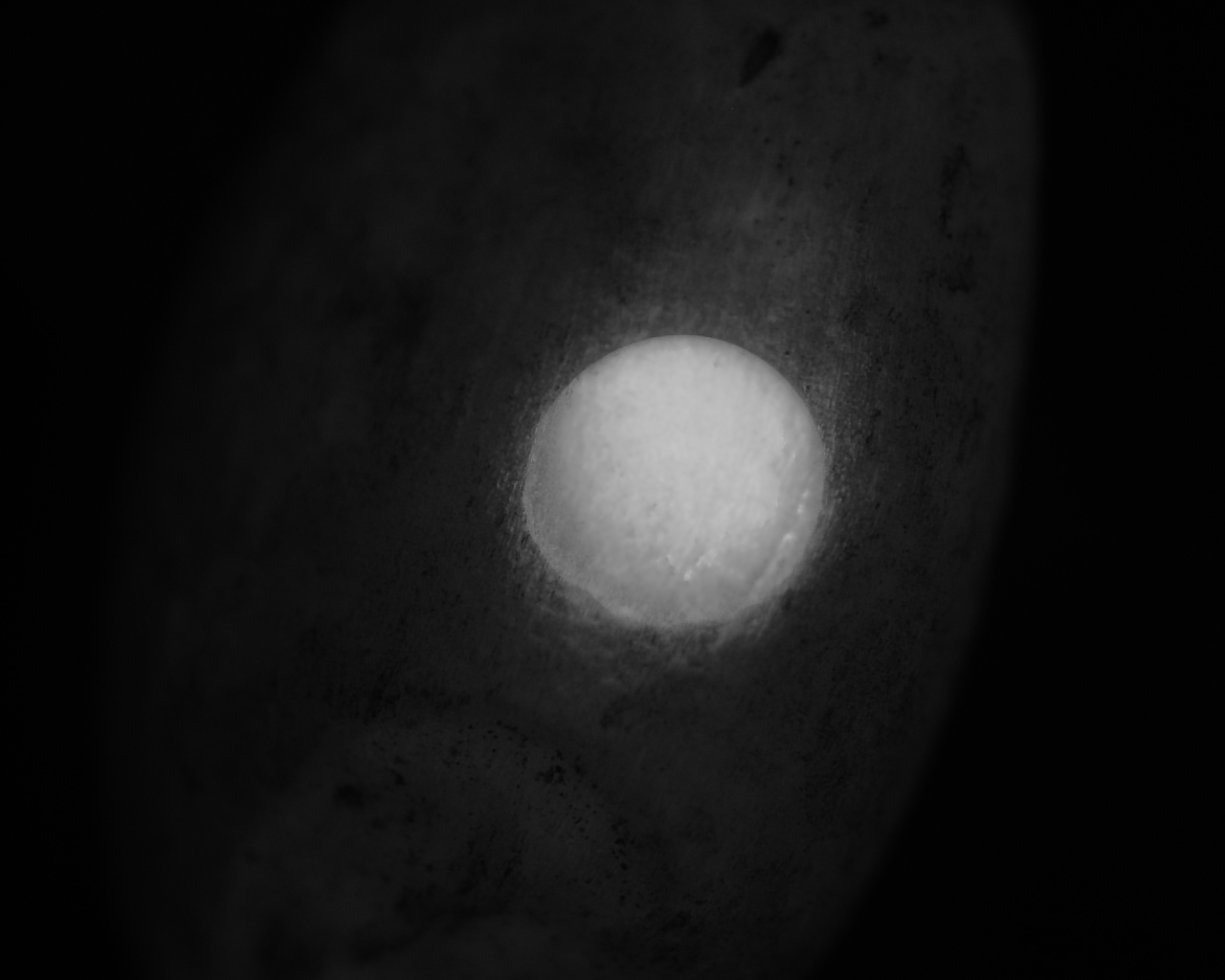

Supplement: S4 File — (ZIP) [file pone.0334274.s004.zip › GezawyDD00 2.tif]

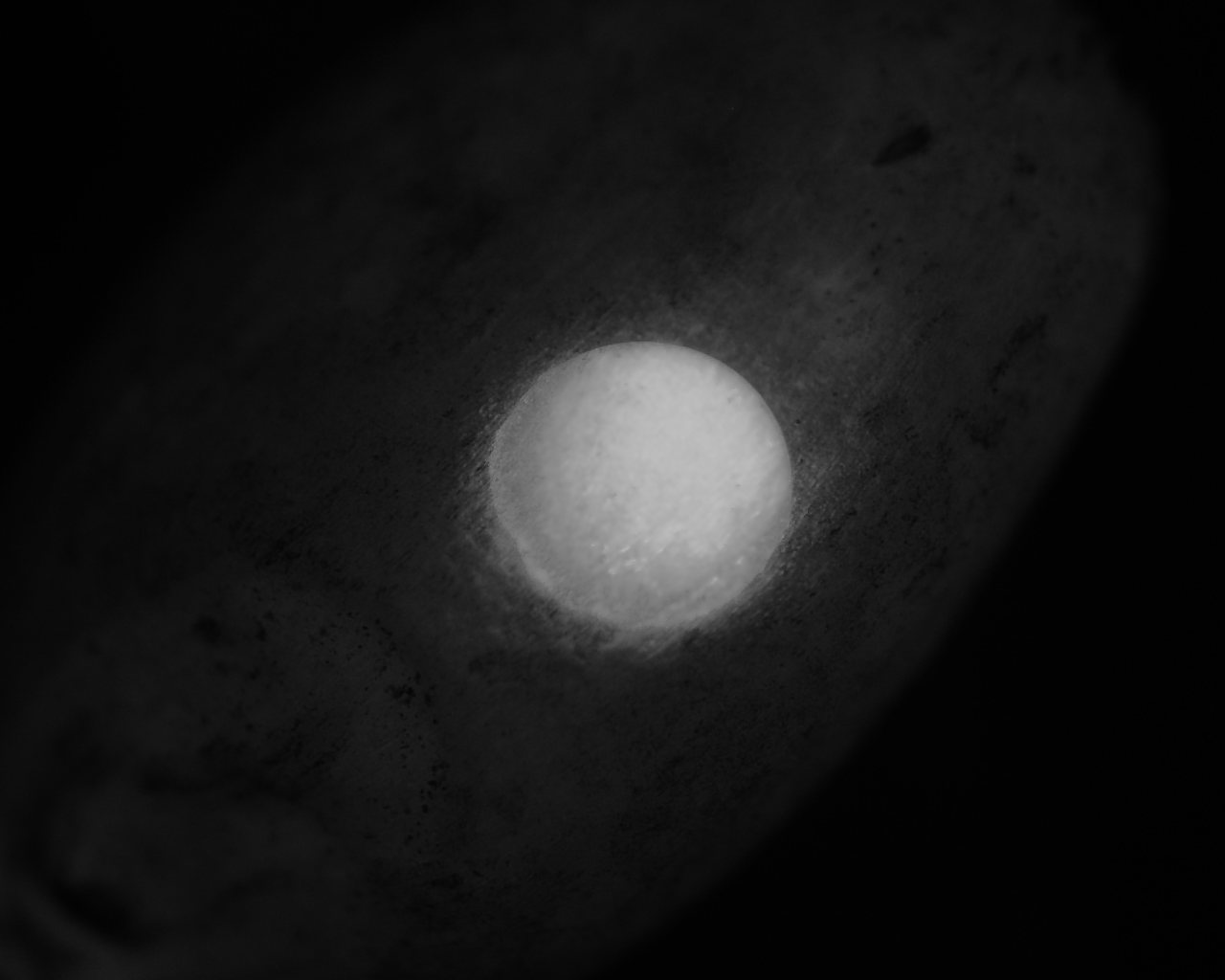

Supplement: S4 File — (ZIP) [file pone.0334274.s004.zip › GezawyDD00 3.tif]

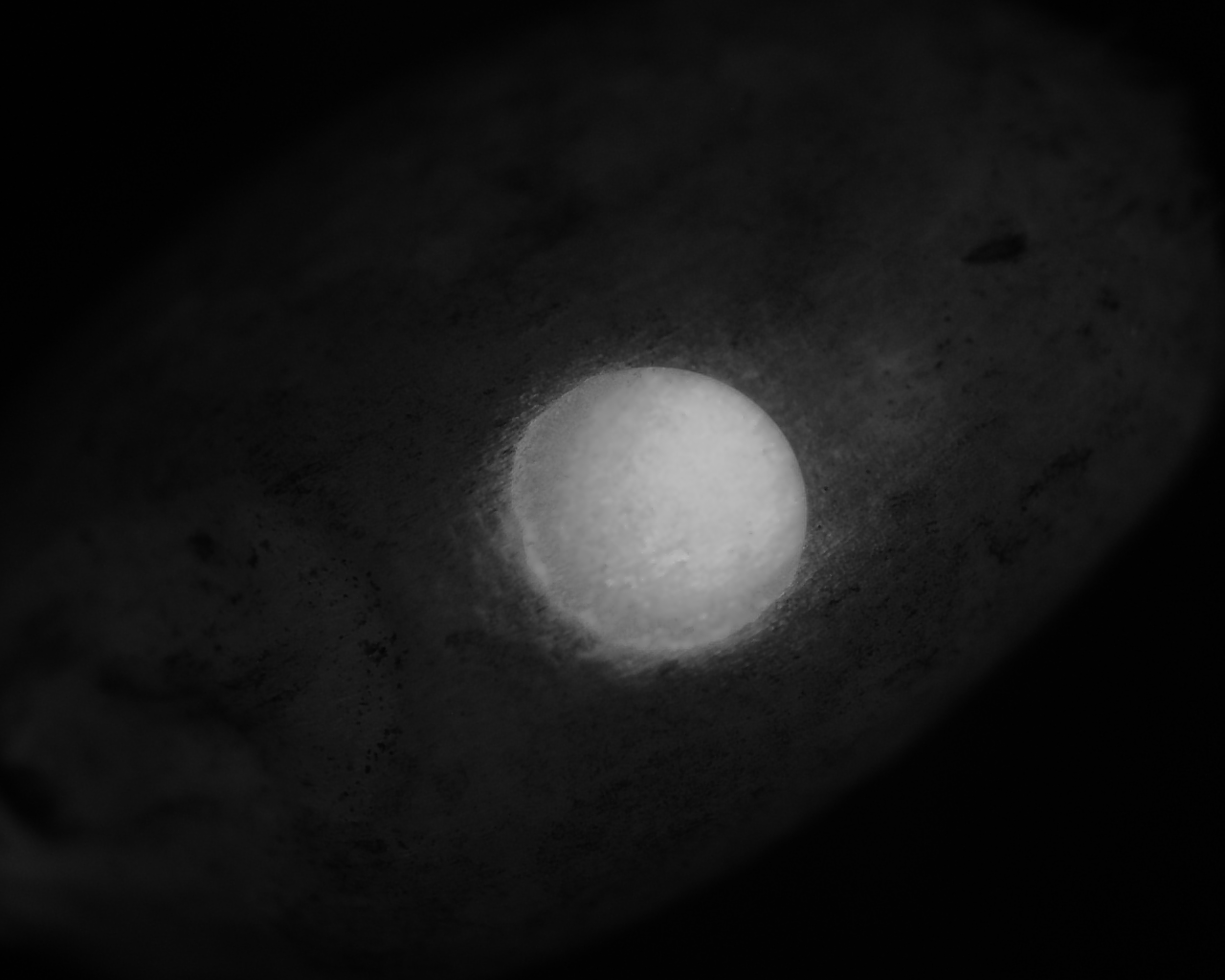

Supplement: S4 File — (ZIP) [file pone.0334274.s004.zip › GezawyDD00 4.tif]

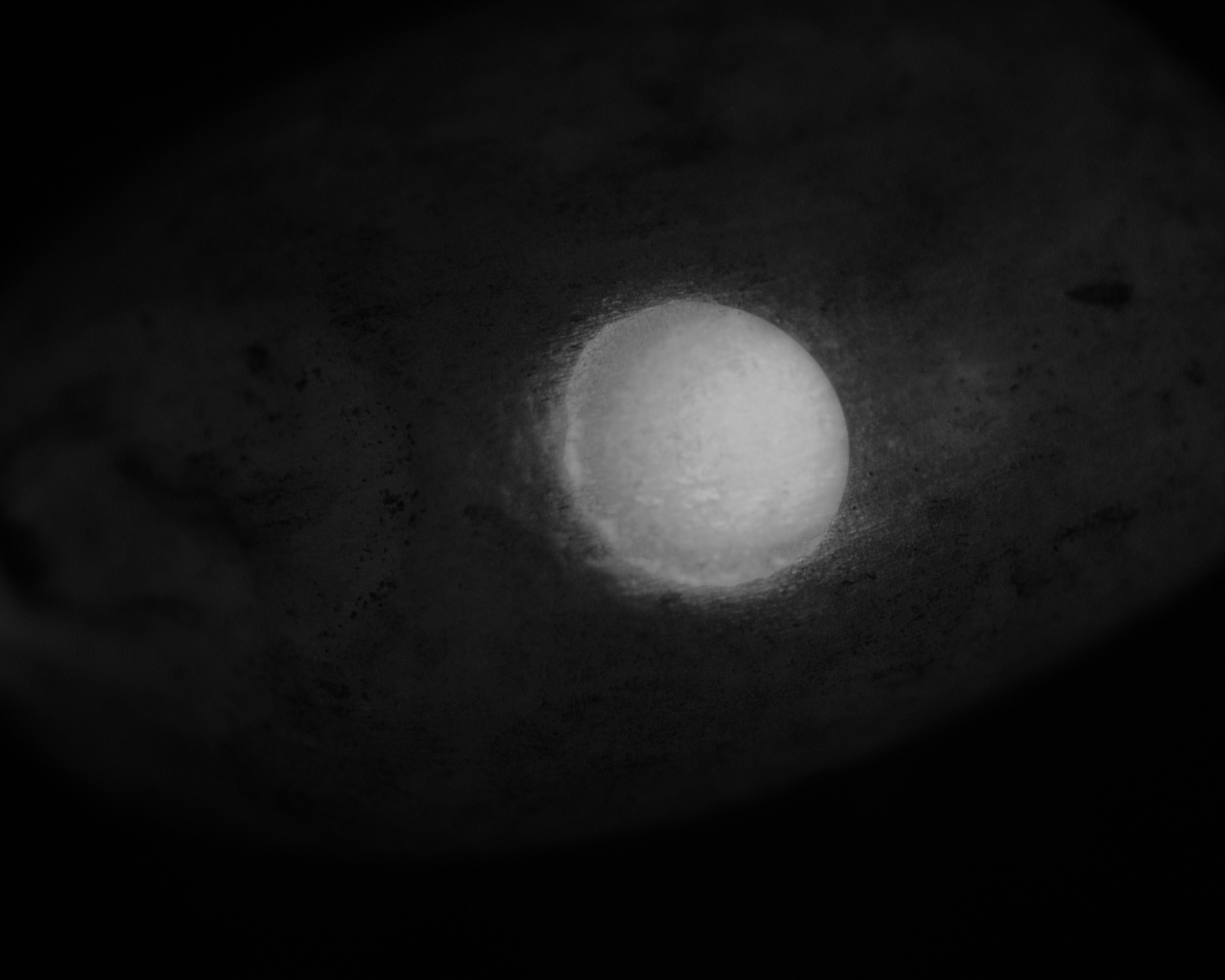

Supplement: S4 File — (ZIP) [file pone.0334274.s004.zip › GezawyDD00 5.tif]

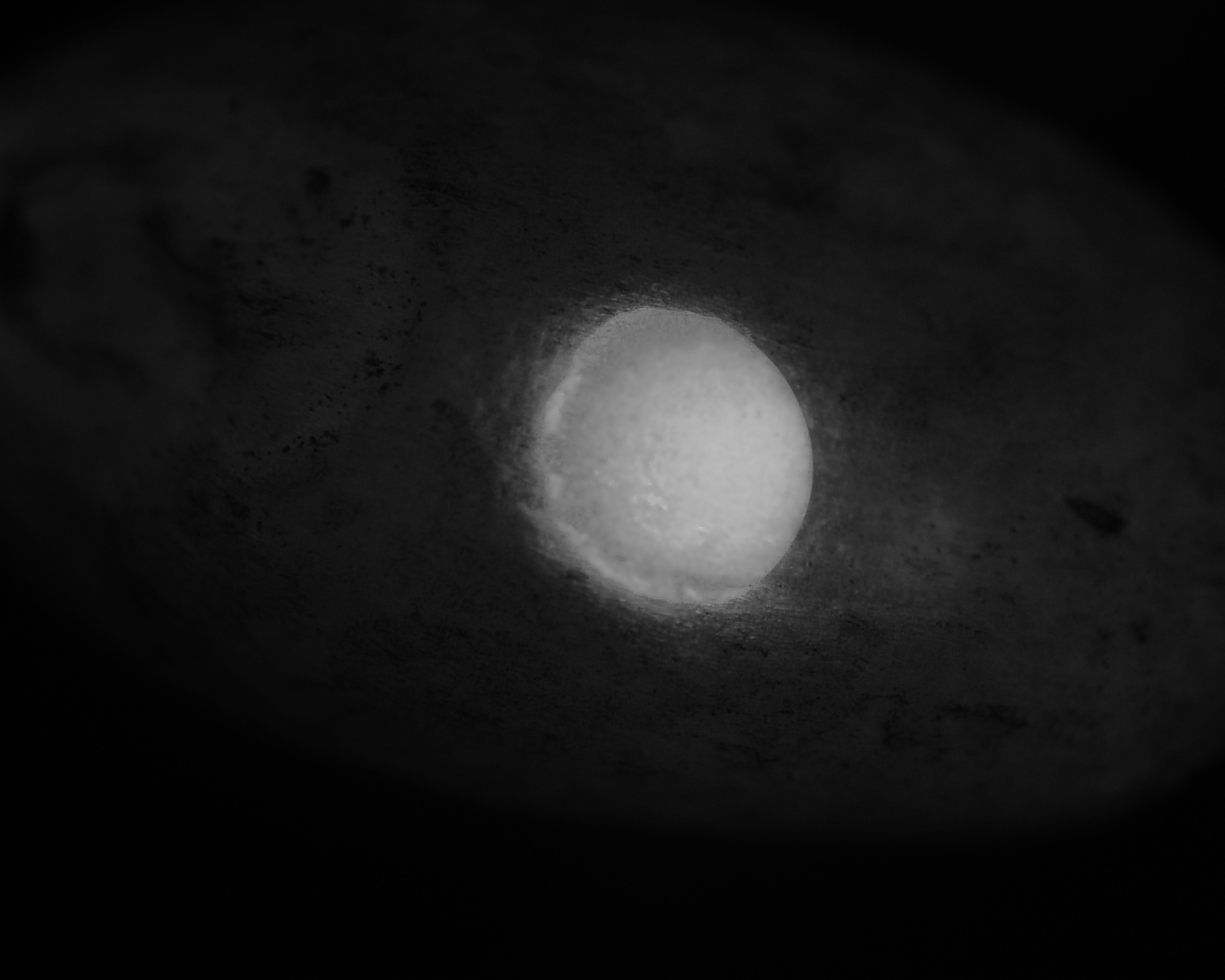

Supplement: S4 File — (ZIP) [file pone.0334274.s004.zip › GezawyDD00 6.tif]

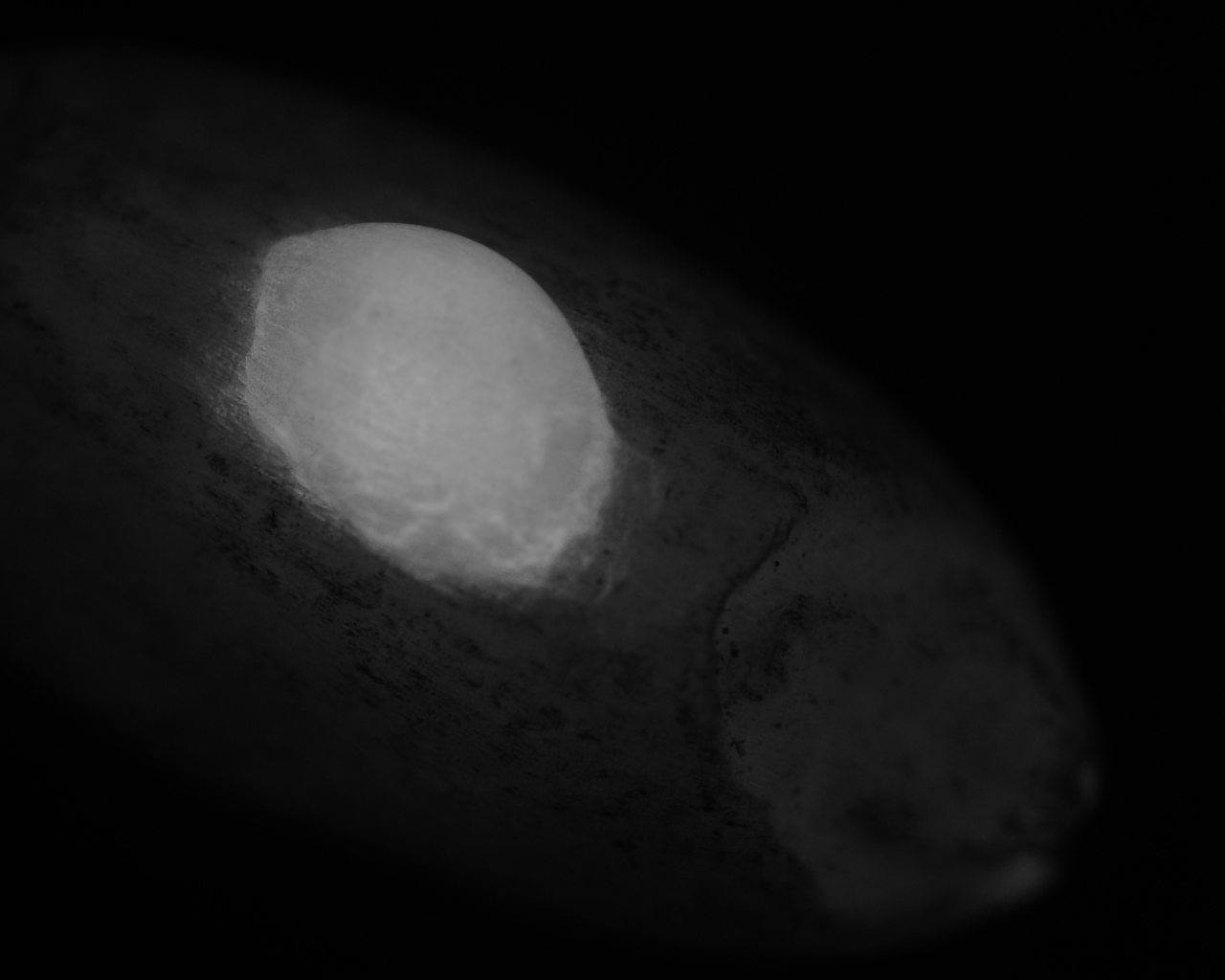

Supplement: S4 File — (ZIP) [file pone.0334274.s004.zip › GezawyDD22 1.tif]

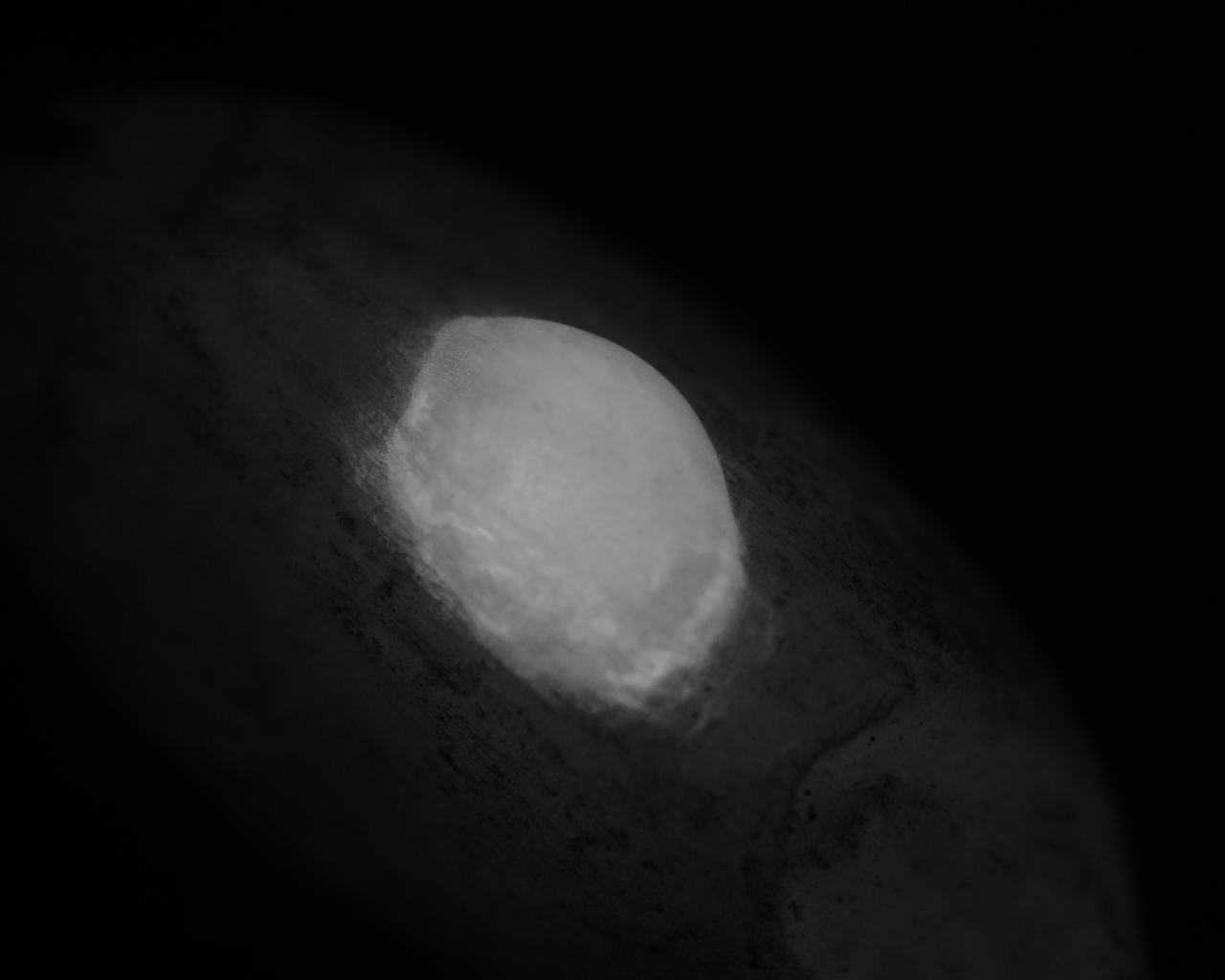

Supplement: S4 File — (ZIP) [file pone.0334274.s004.zip › GezawyDD22 2.tif]

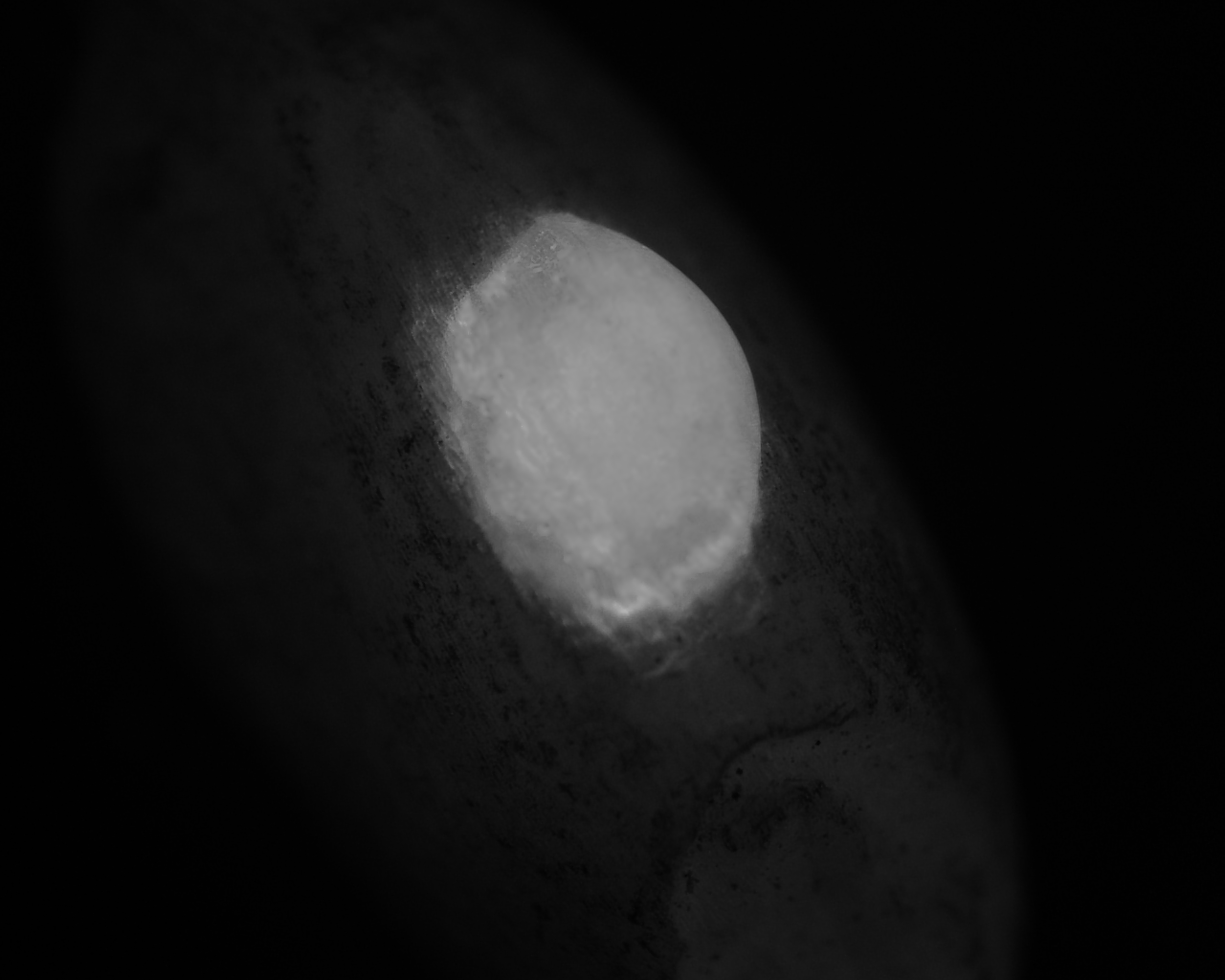

Supplement: S4 File — (ZIP) [file pone.0334274.s004.zip › GezawyDD22 3.tif]

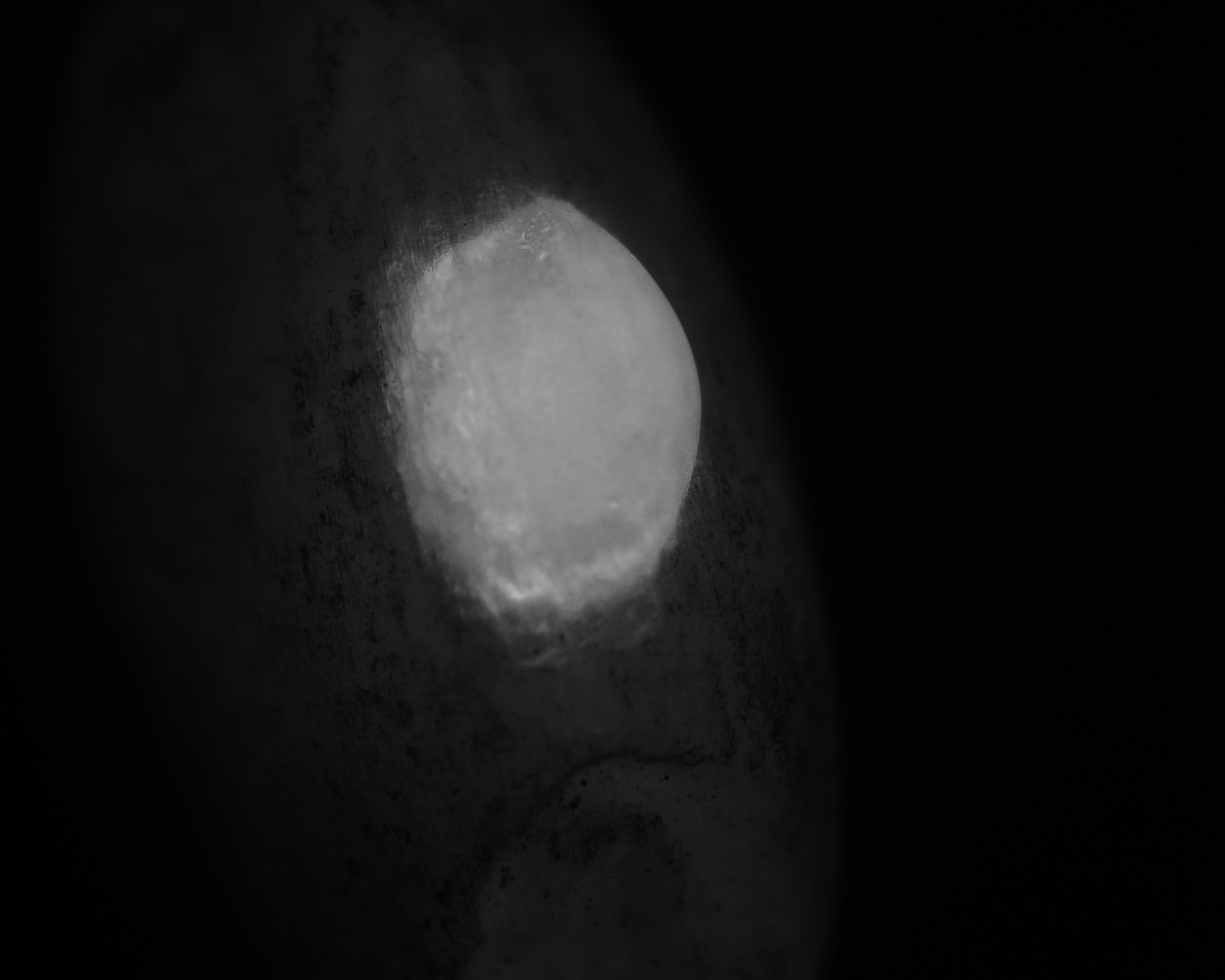

Supplement: S4 File — (ZIP) [file pone.0334274.s004.zip › GezawyDD22 4.tif]

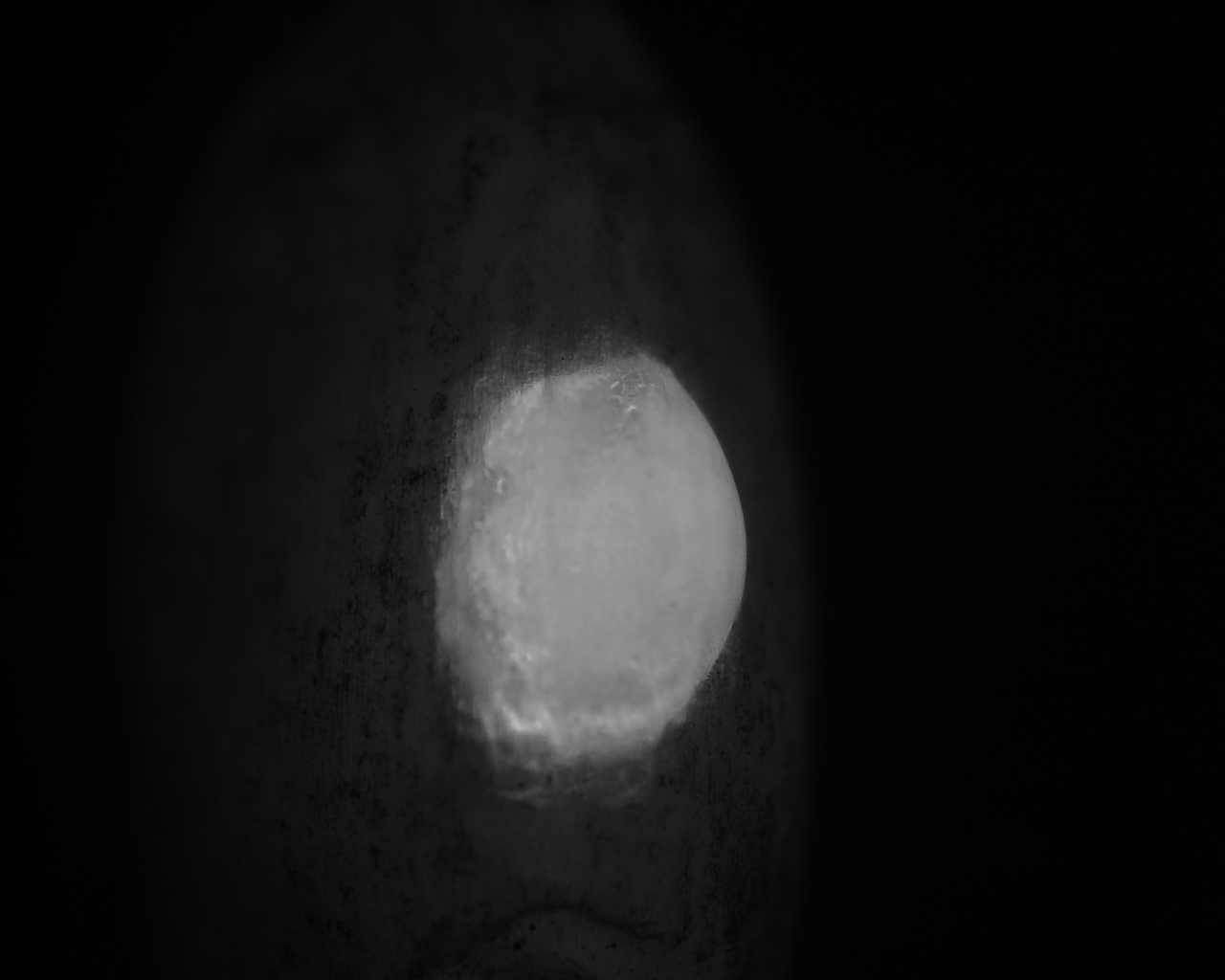

Supplement: S4 File — (ZIP) [file pone.0334274.s004.zip › GezawyDD22 5.tif]

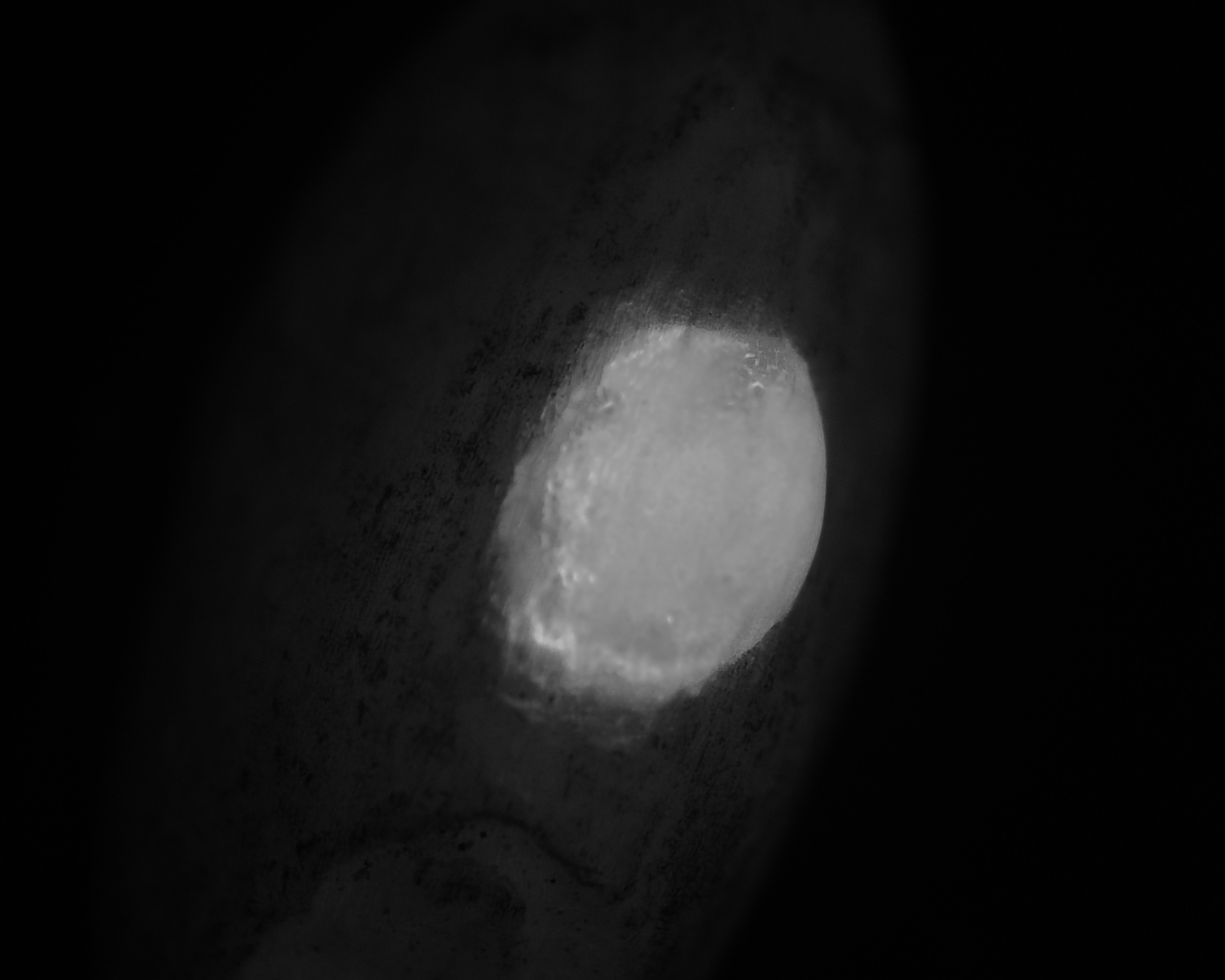

Supplement: S4 File — (ZIP) [file pone.0334274.s004.zip › GezawyDD22 6.tif]

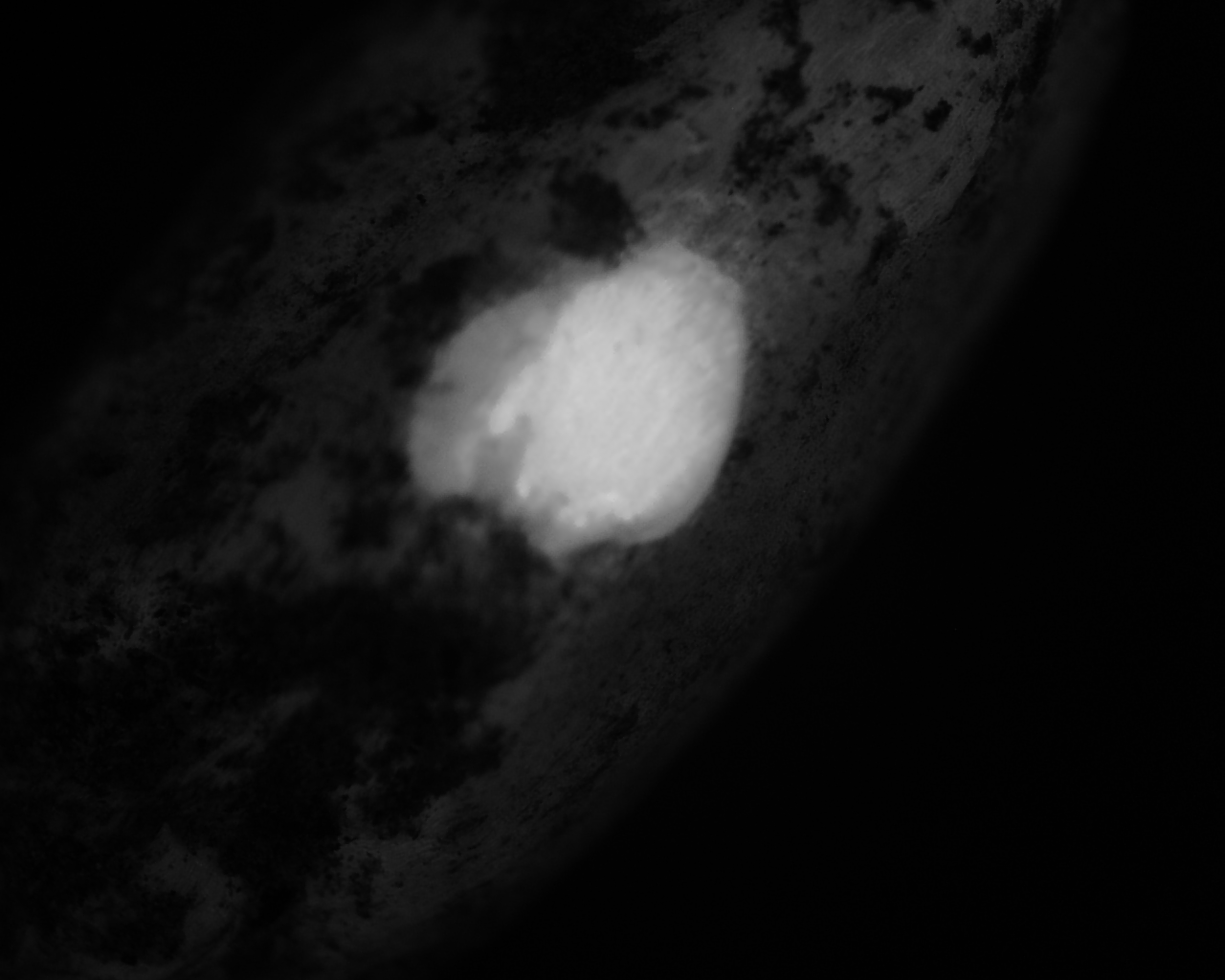

Supplement: S4 File — (ZIP) [file pone.0334274.s004.zip › GezawyDD33 1.tif]

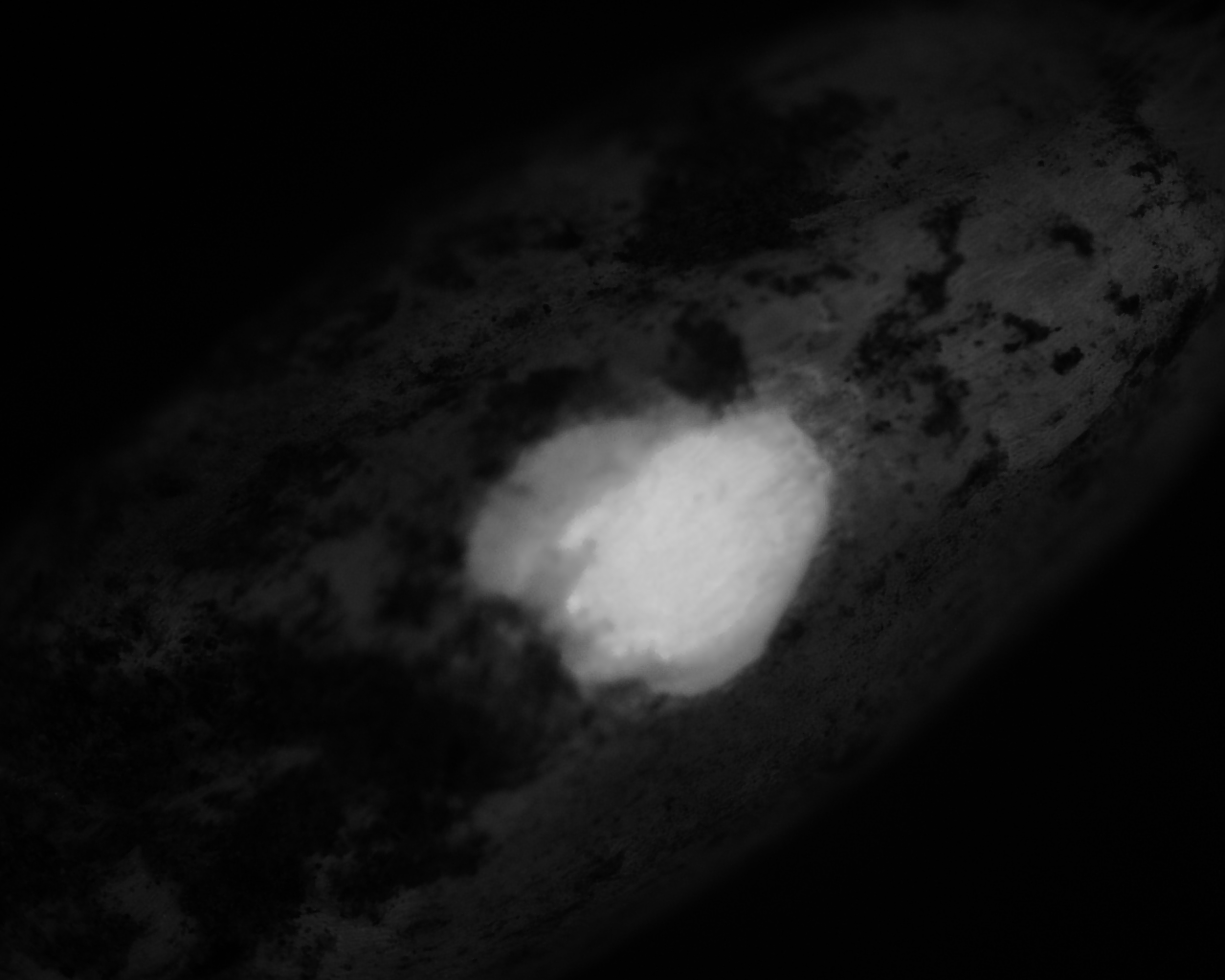

Supplement: S4 File — (ZIP) [file pone.0334274.s004.zip › GezawyDD33 2.tif]

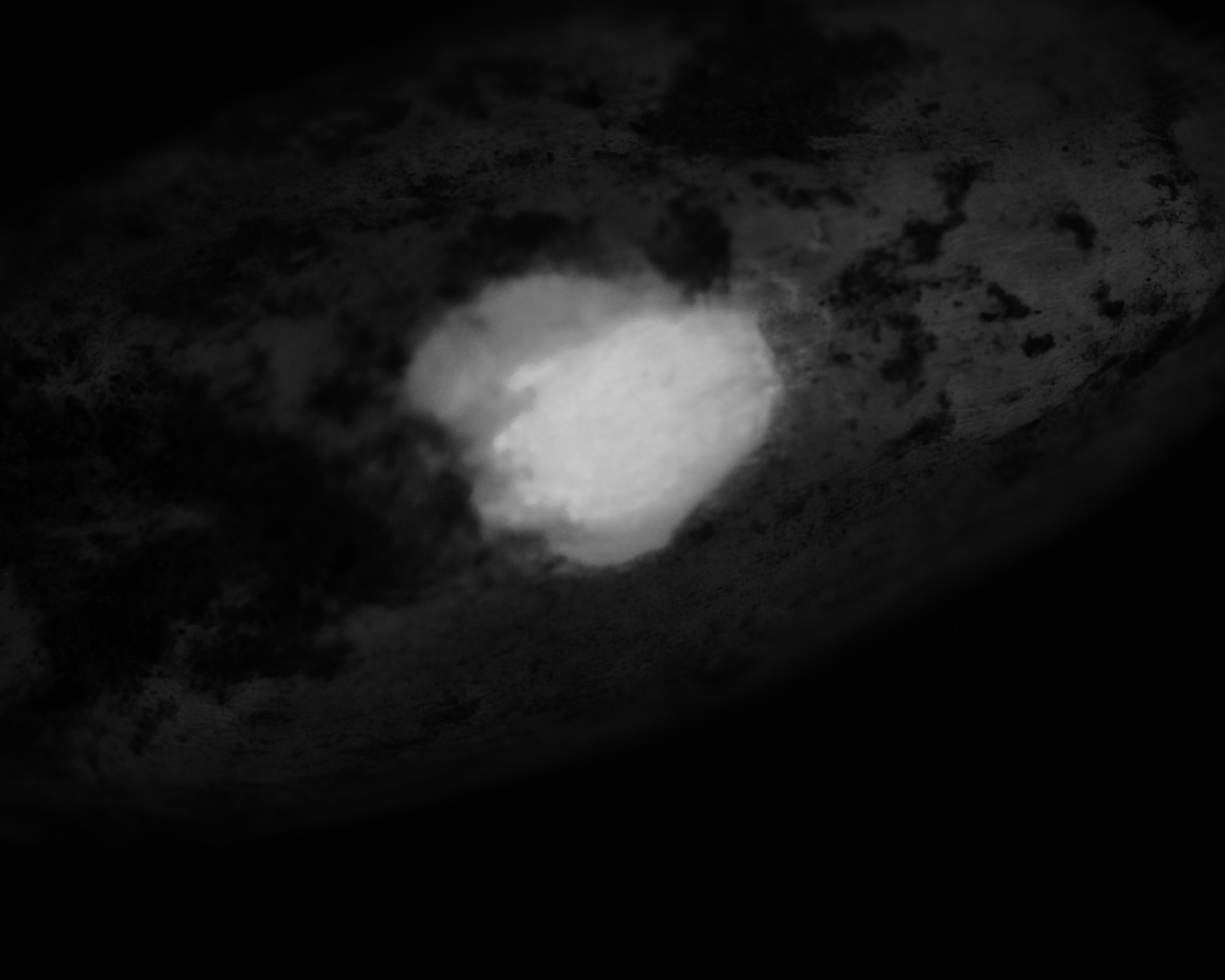

Supplement: S4 File — (ZIP) [file pone.0334274.s004.zip › GezawyDD33 3.tif]

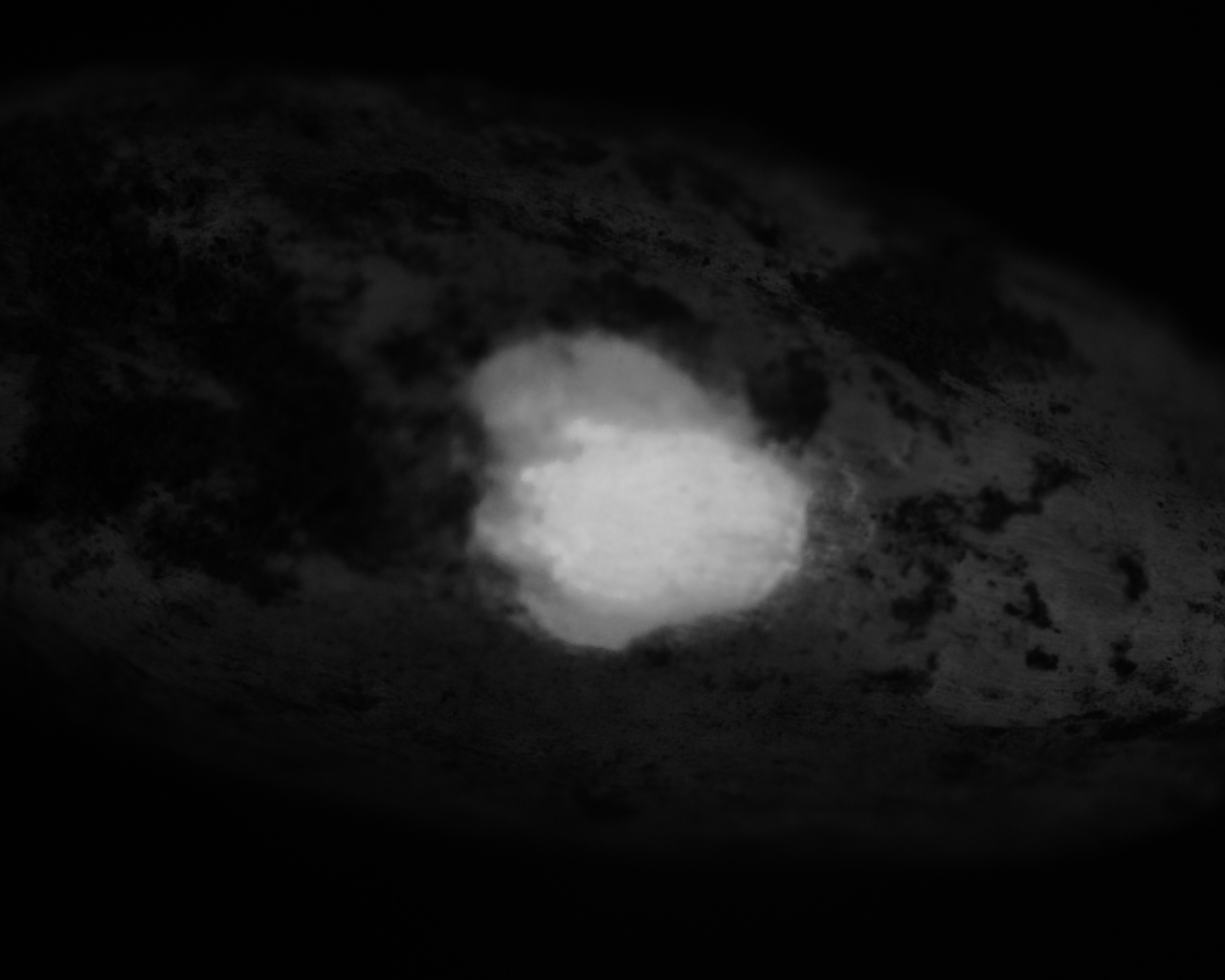

Supplement: S4 File — (ZIP) [file pone.0334274.s004.zip › GezawyDD33 4.tif]

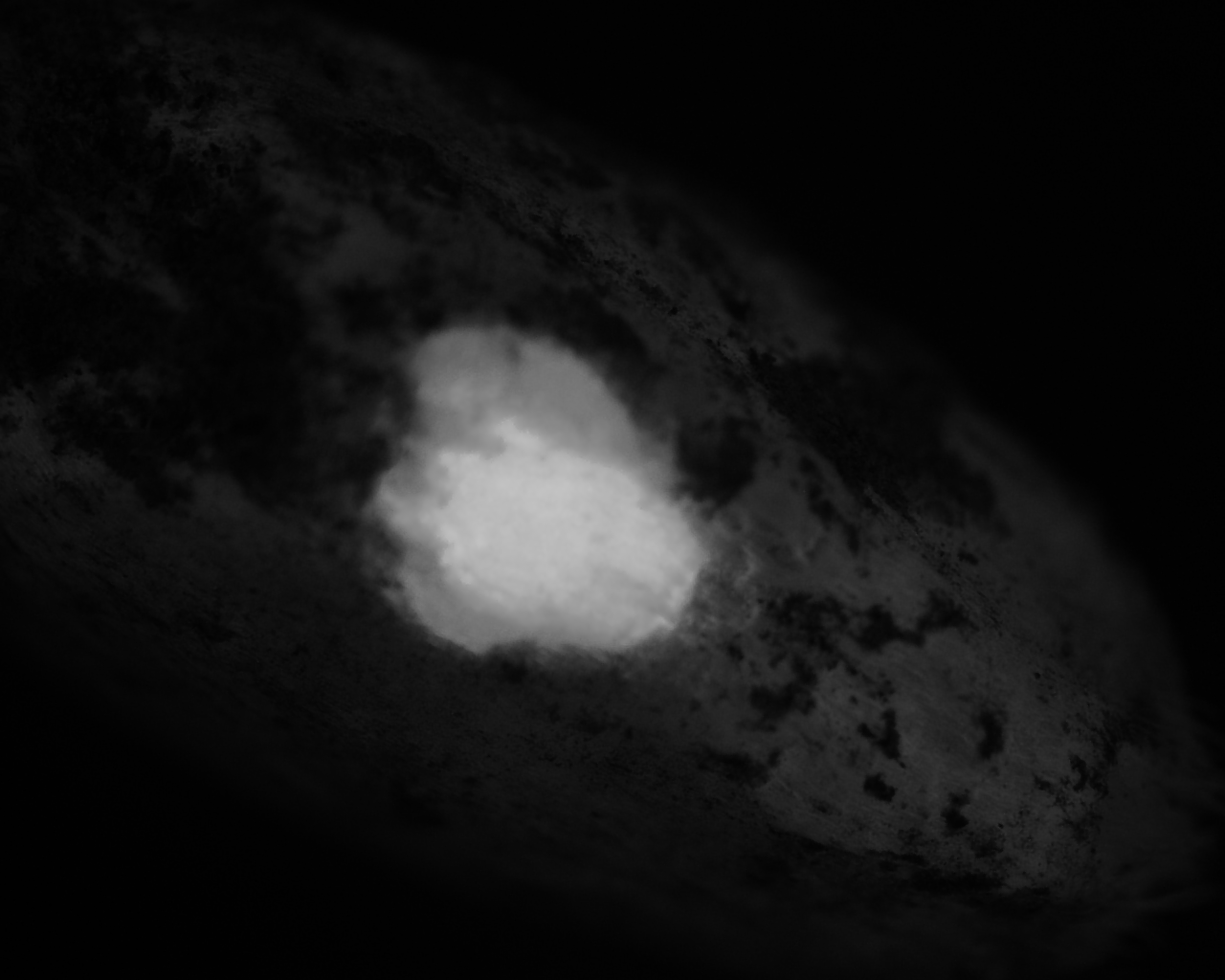

Supplement: S4 File — (ZIP) [file pone.0334274.s004.zip › GezawyDD33 5.tif]

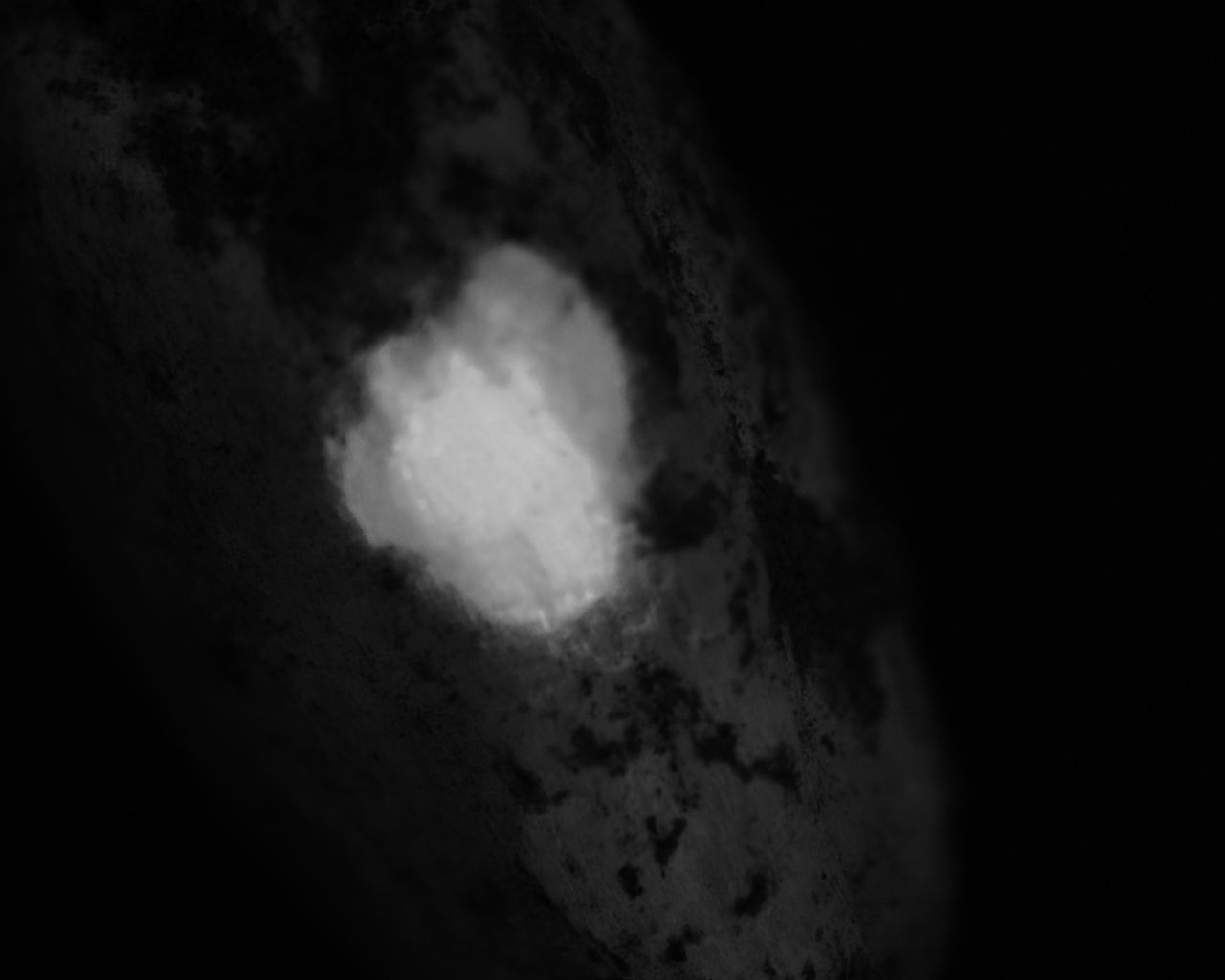

Supplement: S4 File — (ZIP) [file pone.0334274.s004.zip › GezawyDD33 6.tif]

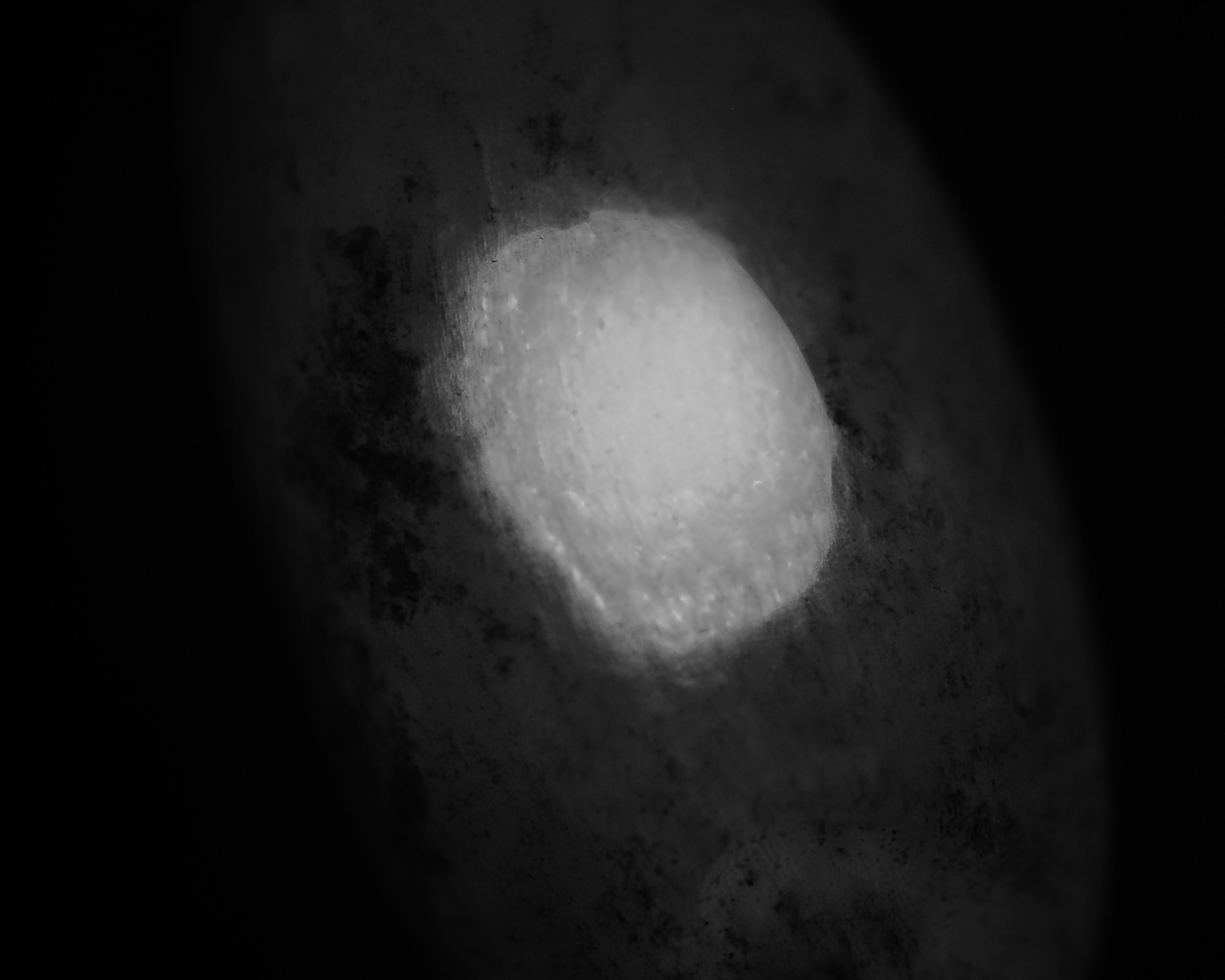

Supplement: S4 File — (ZIP) [file pone.0334274.s004.zip › GezawyDD44 1.tif]

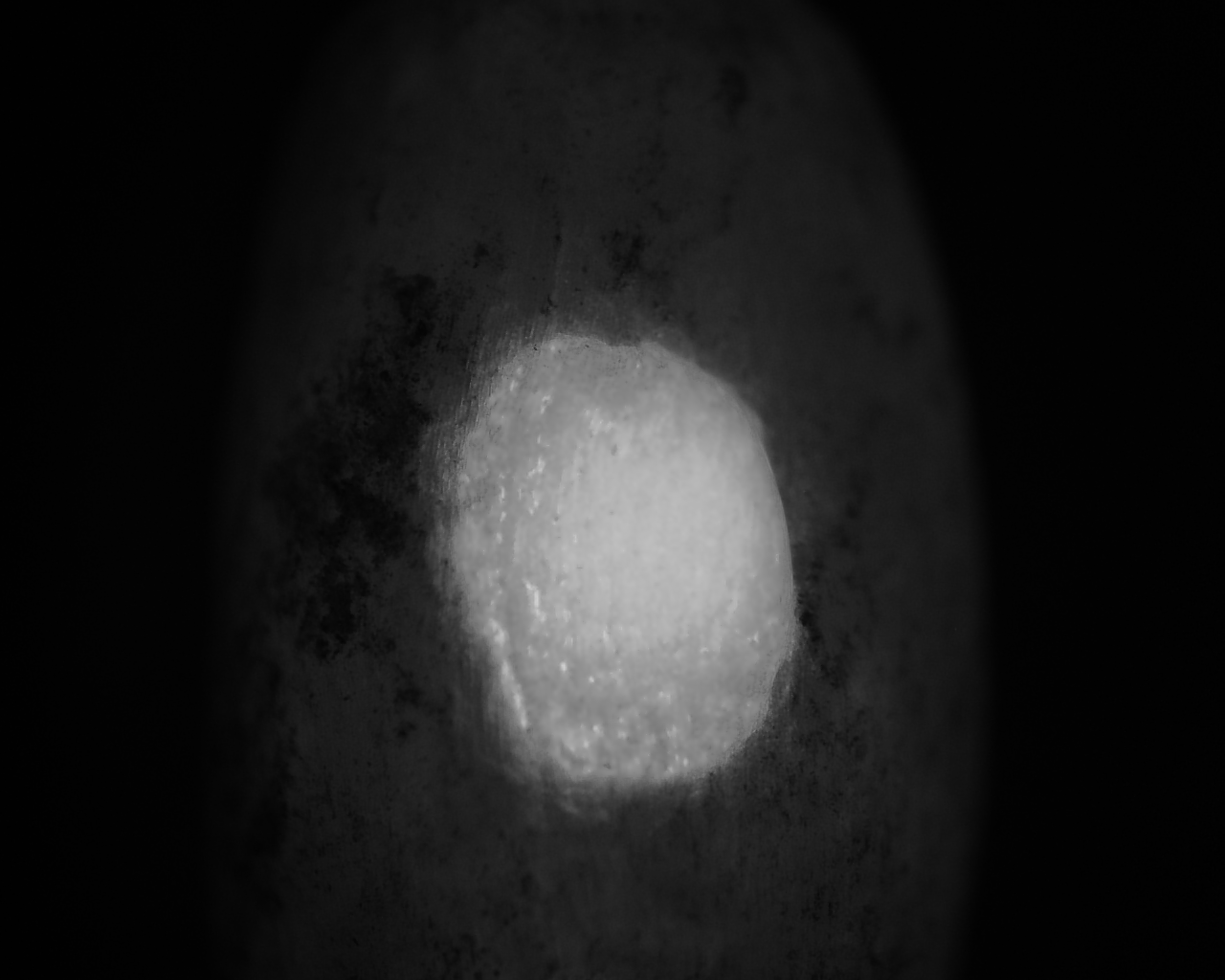

Supplement: S4 File — (ZIP) [file pone.0334274.s004.zip › GezawyDD44 2.tif]

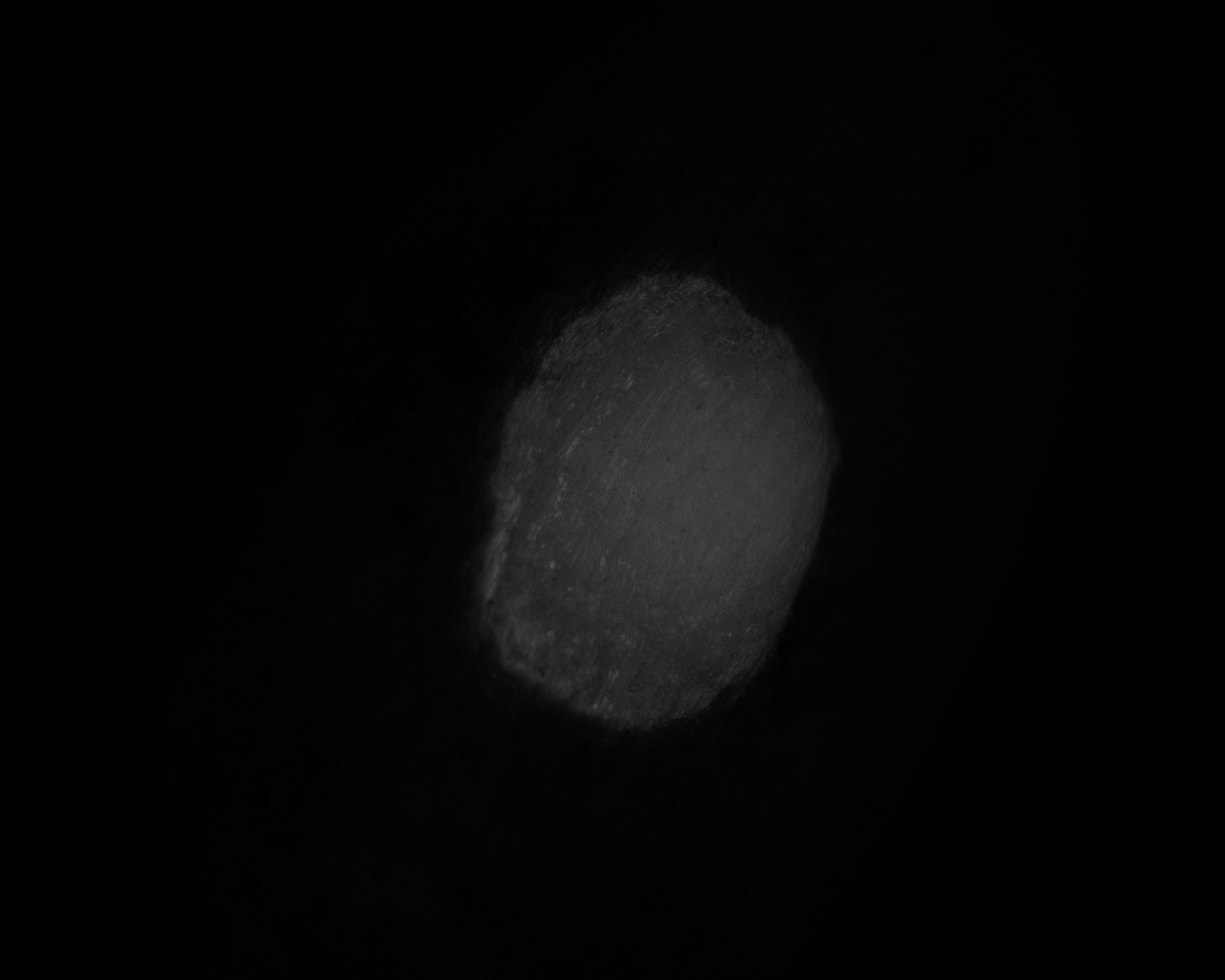

Supplement: S4 File — (ZIP) [file pone.0334274.s004.zip › GezawyDD44 3.tif]

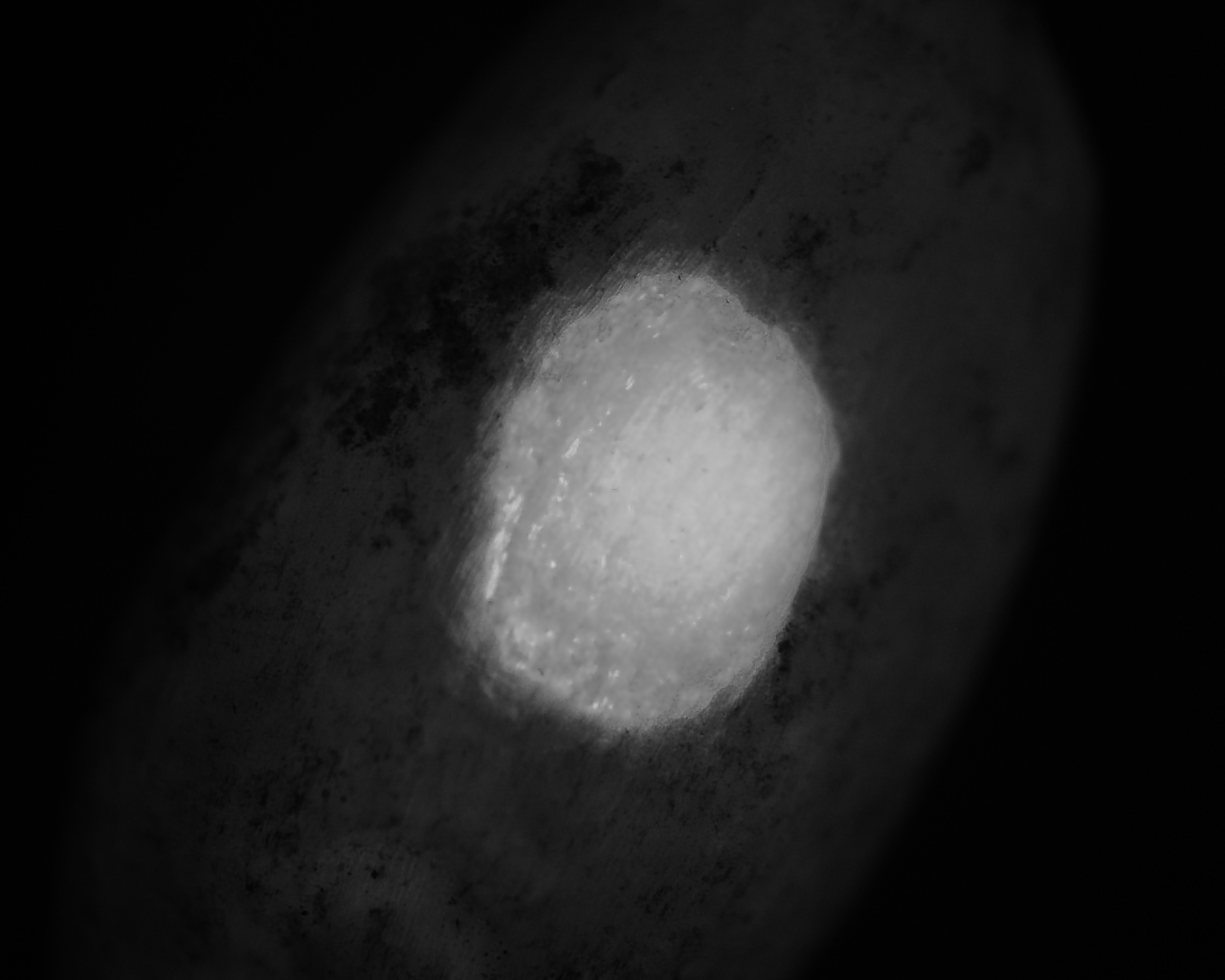

Supplement: S4 File — (ZIP) [file pone.0334274.s004.zip › GezawyDD44 33.tif]

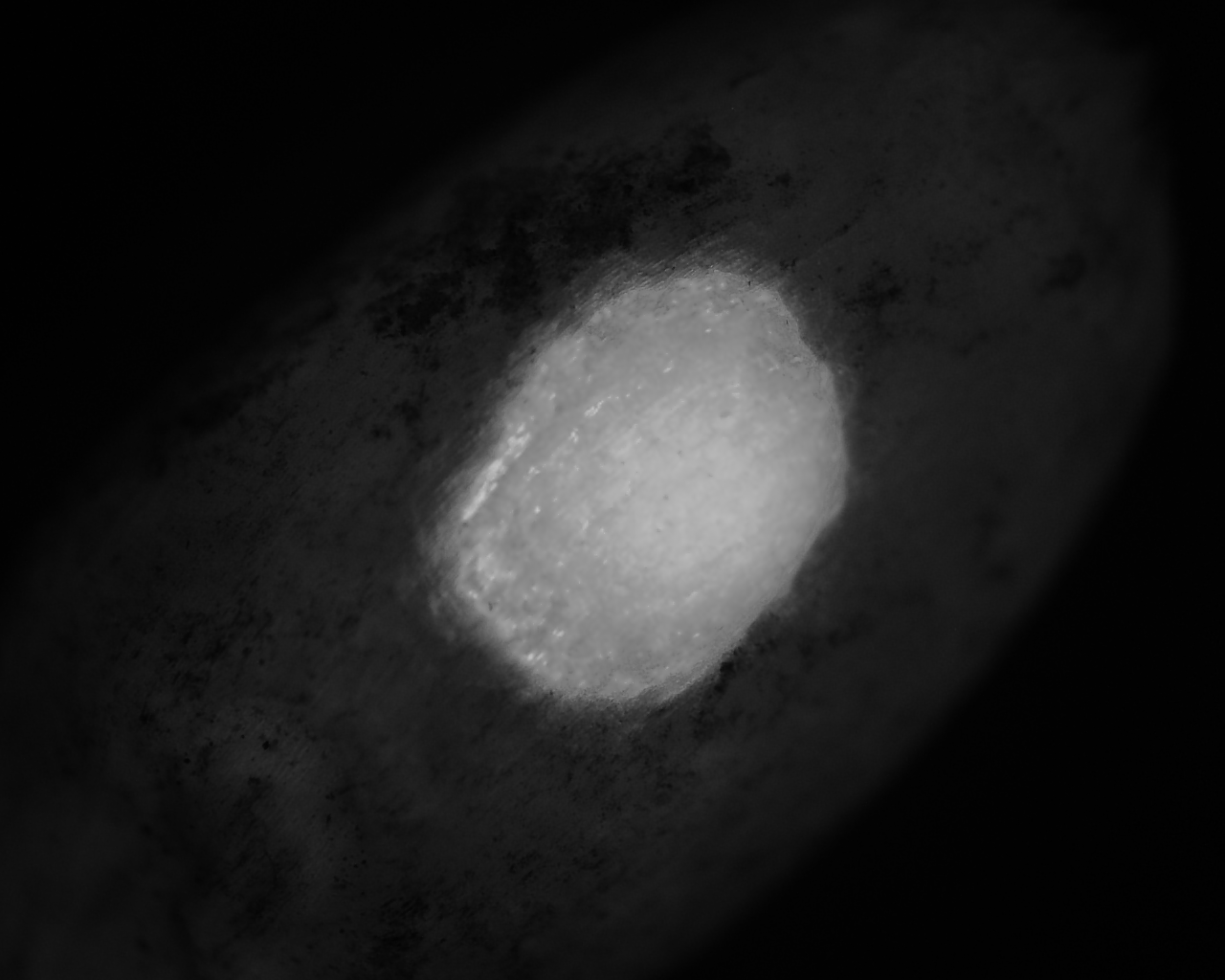

Supplement: S4 File — (ZIP) [file pone.0334274.s004.zip › GezawyDD44 4.tif]

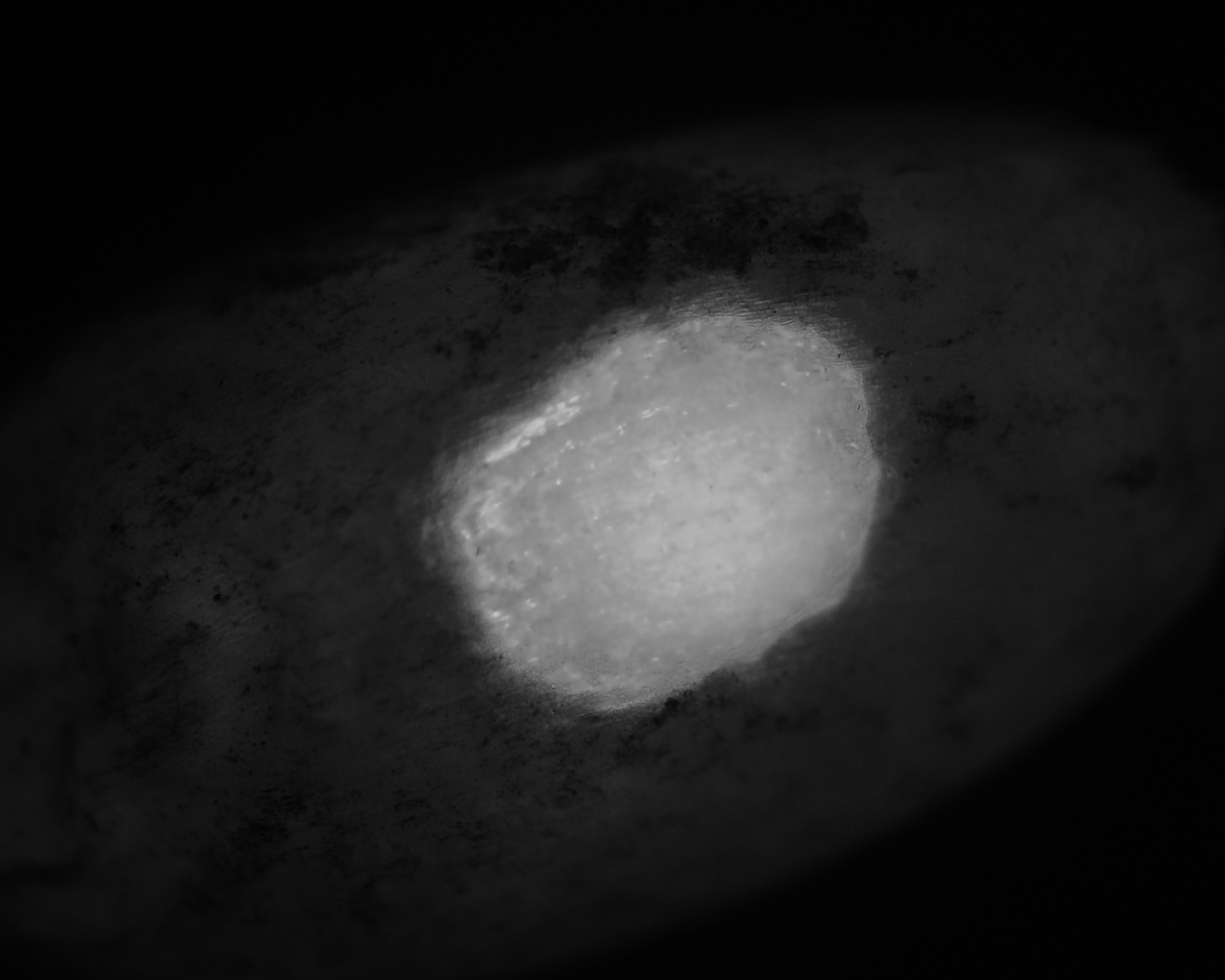

Supplement: S4 File — (ZIP) [file pone.0334274.s004.zip › GezawyDD44 5.tif]

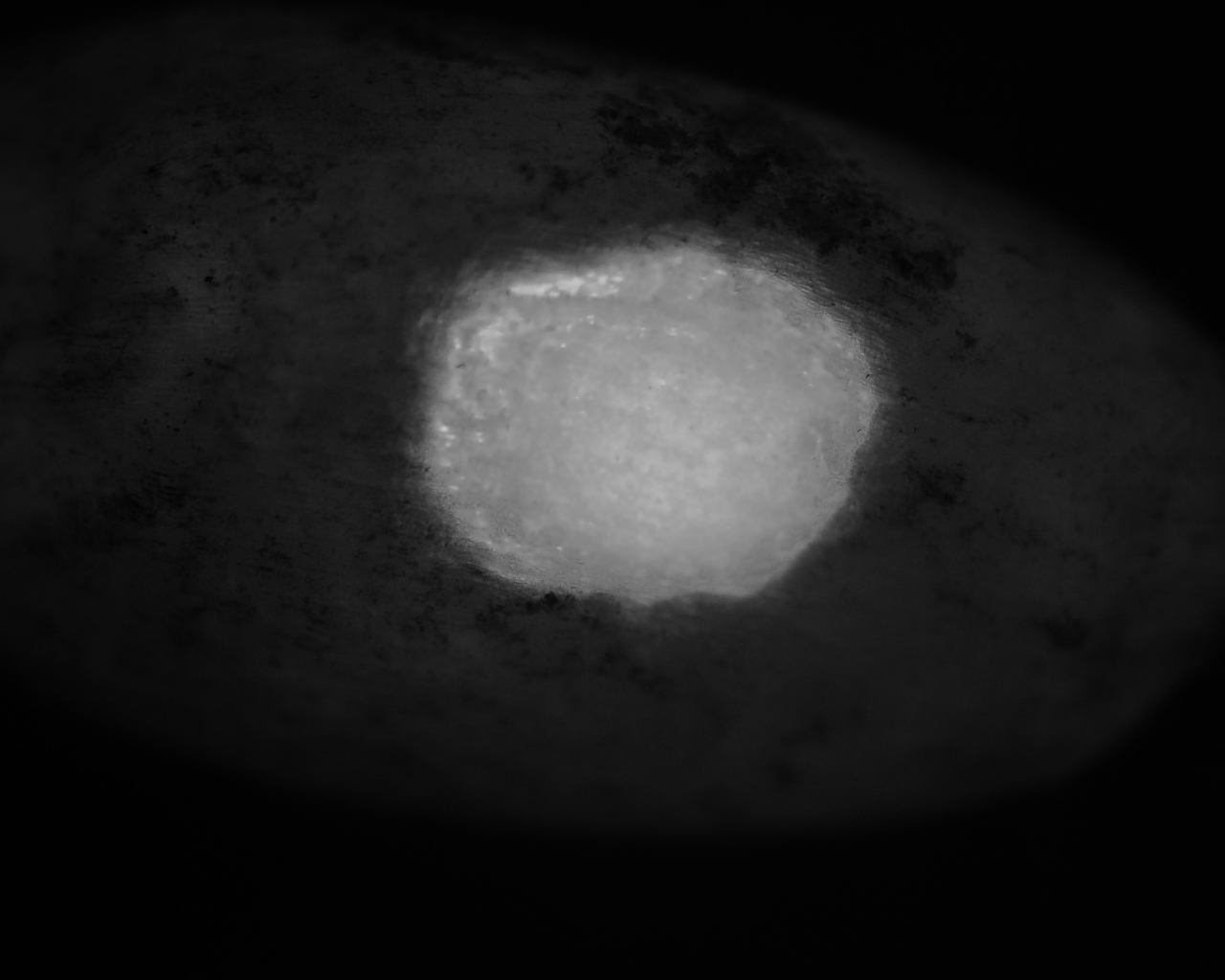

Supplement: S4 File — (ZIP) [file pone.0334274.s004.zip › GezawyDD44 6.tif]

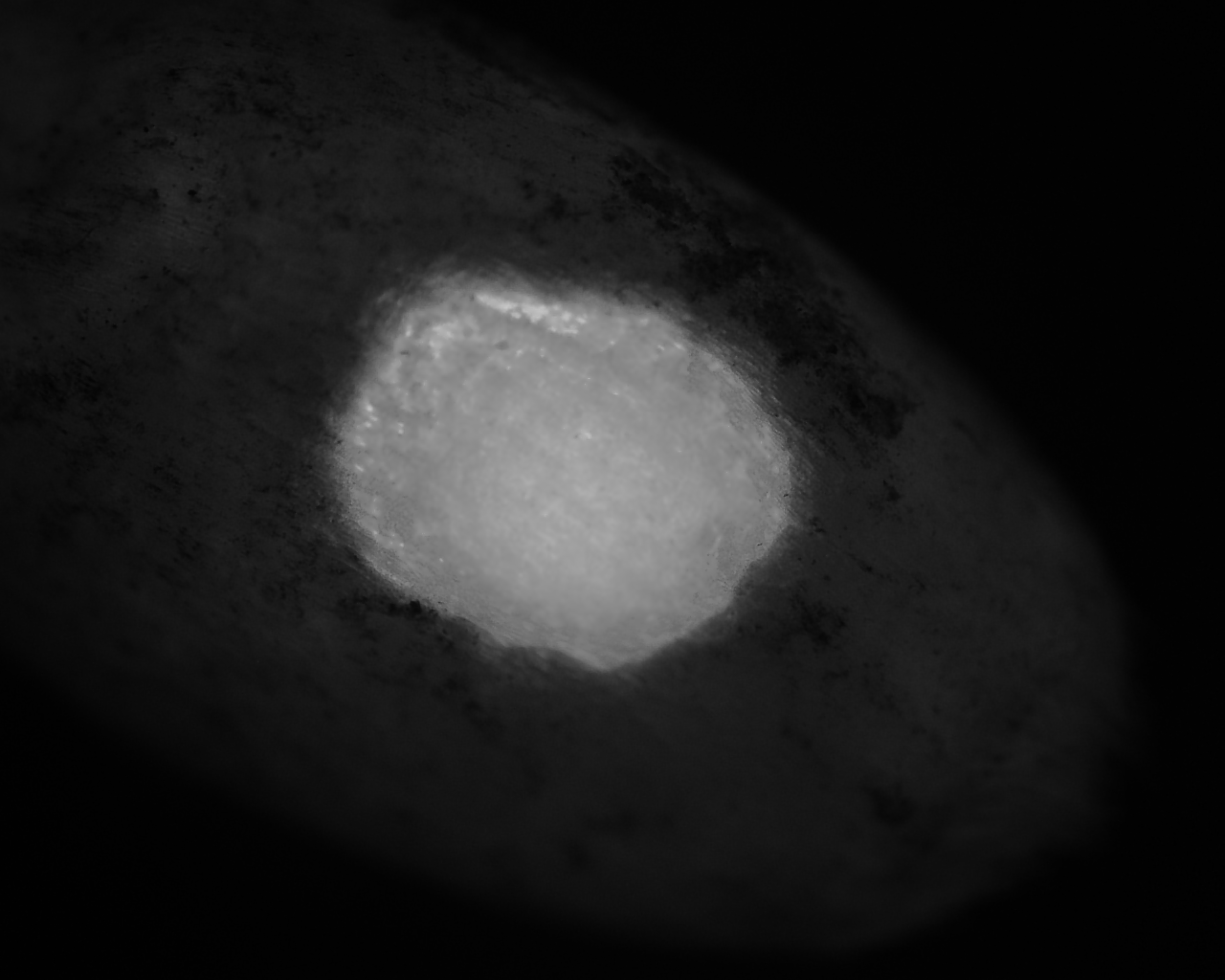

Supplement: S4 File — (ZIP) [file pone.0334274.s004.zip › GezawyDD44 7.tif]

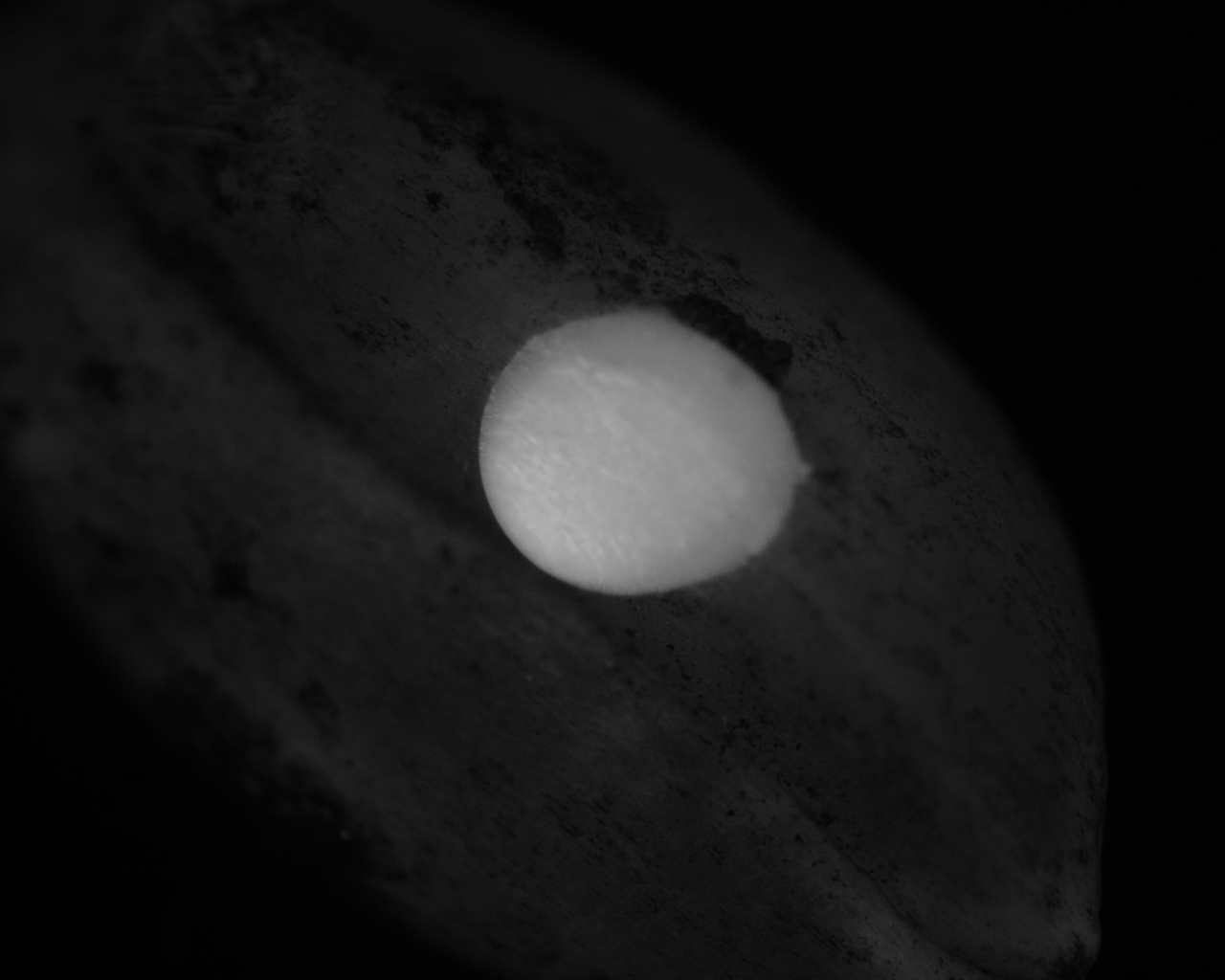

Supplement: S4 File — (ZIP) [file pone.0334274.s004.zip › GezawyDD55 1.tif]

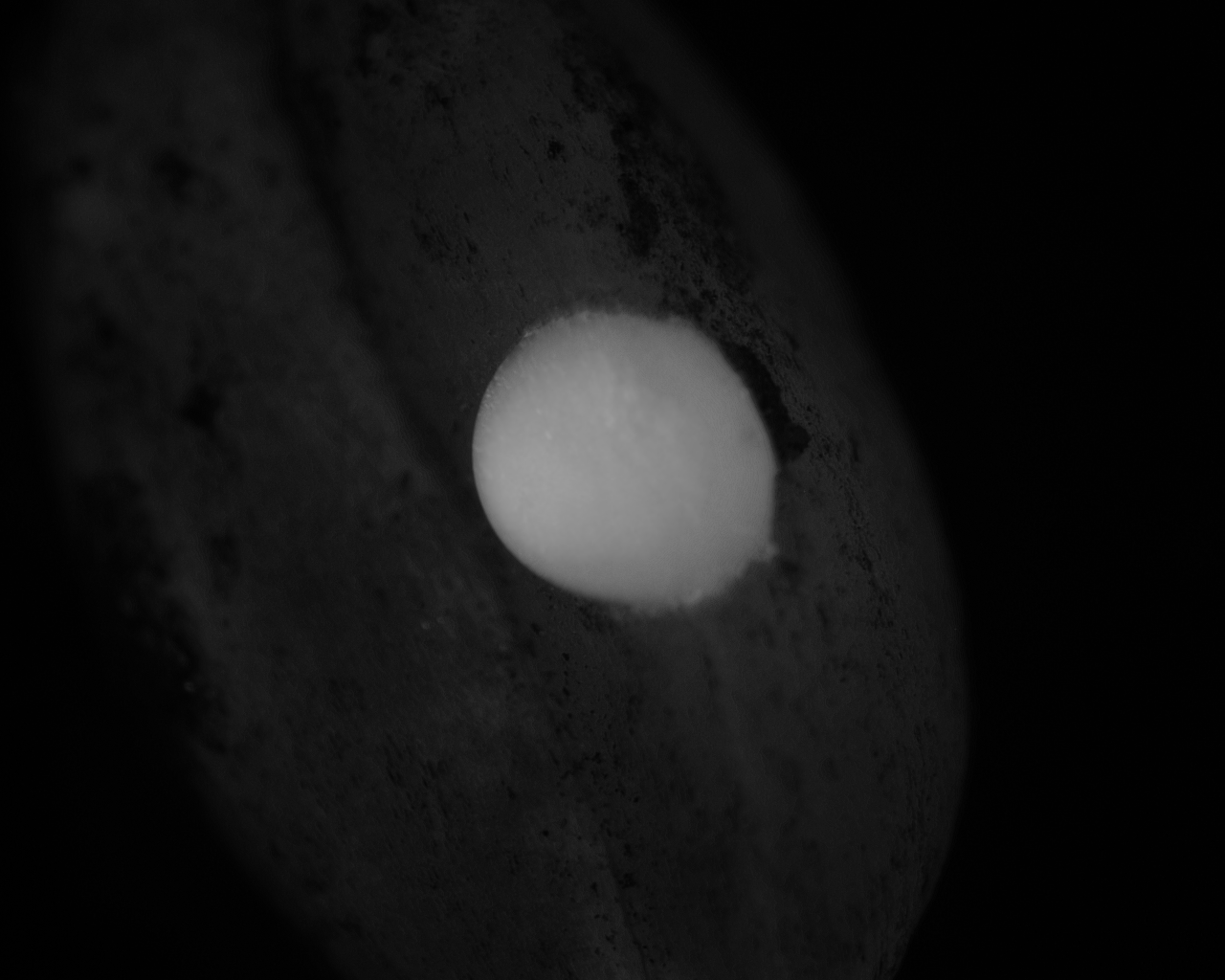

Supplement: S4 File — (ZIP) [file pone.0334274.s004.zip › GezawyDD55 2.tif]

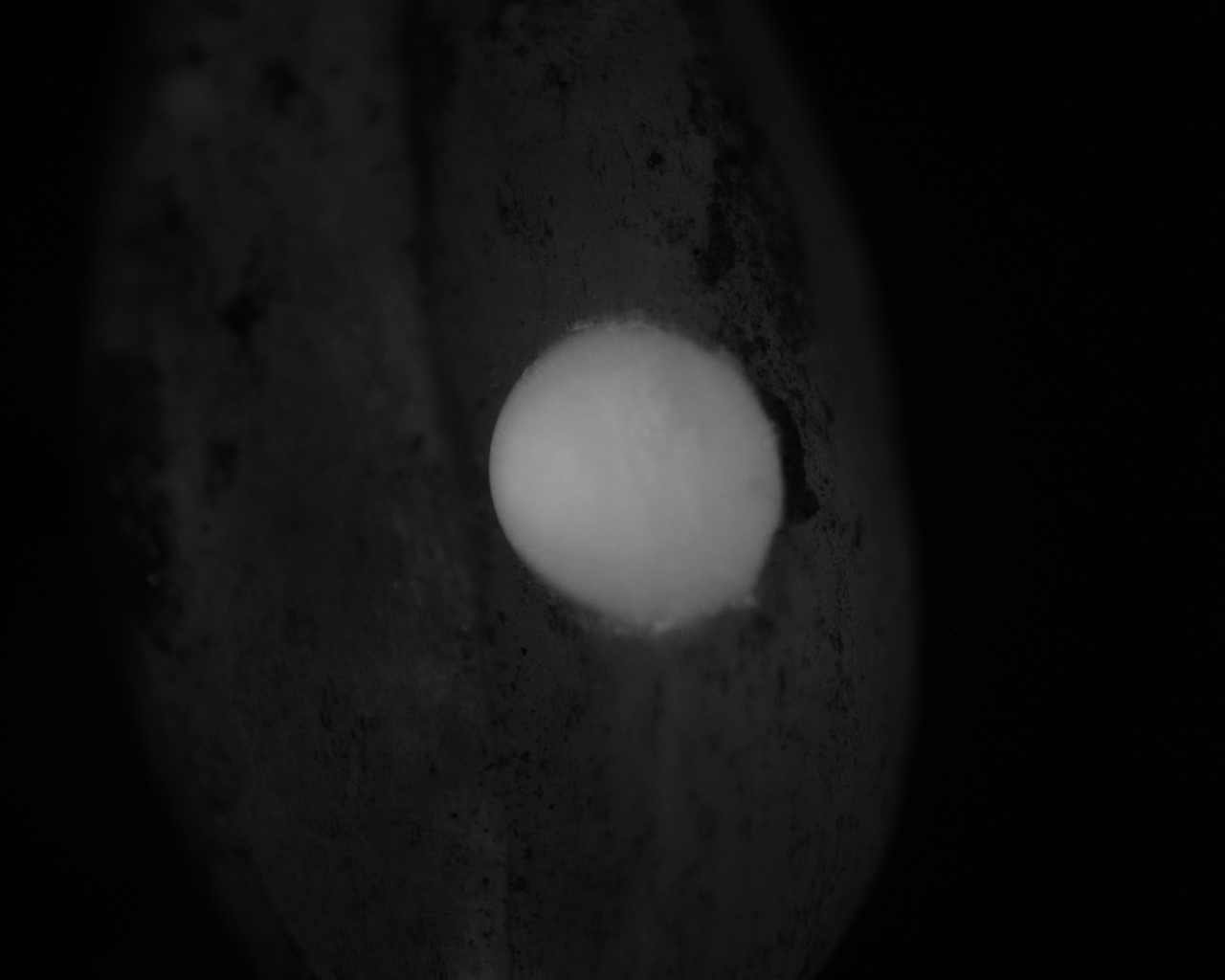

Supplement: S4 File — (ZIP) [file pone.0334274.s004.zip › GezawyDD55 3.tif]

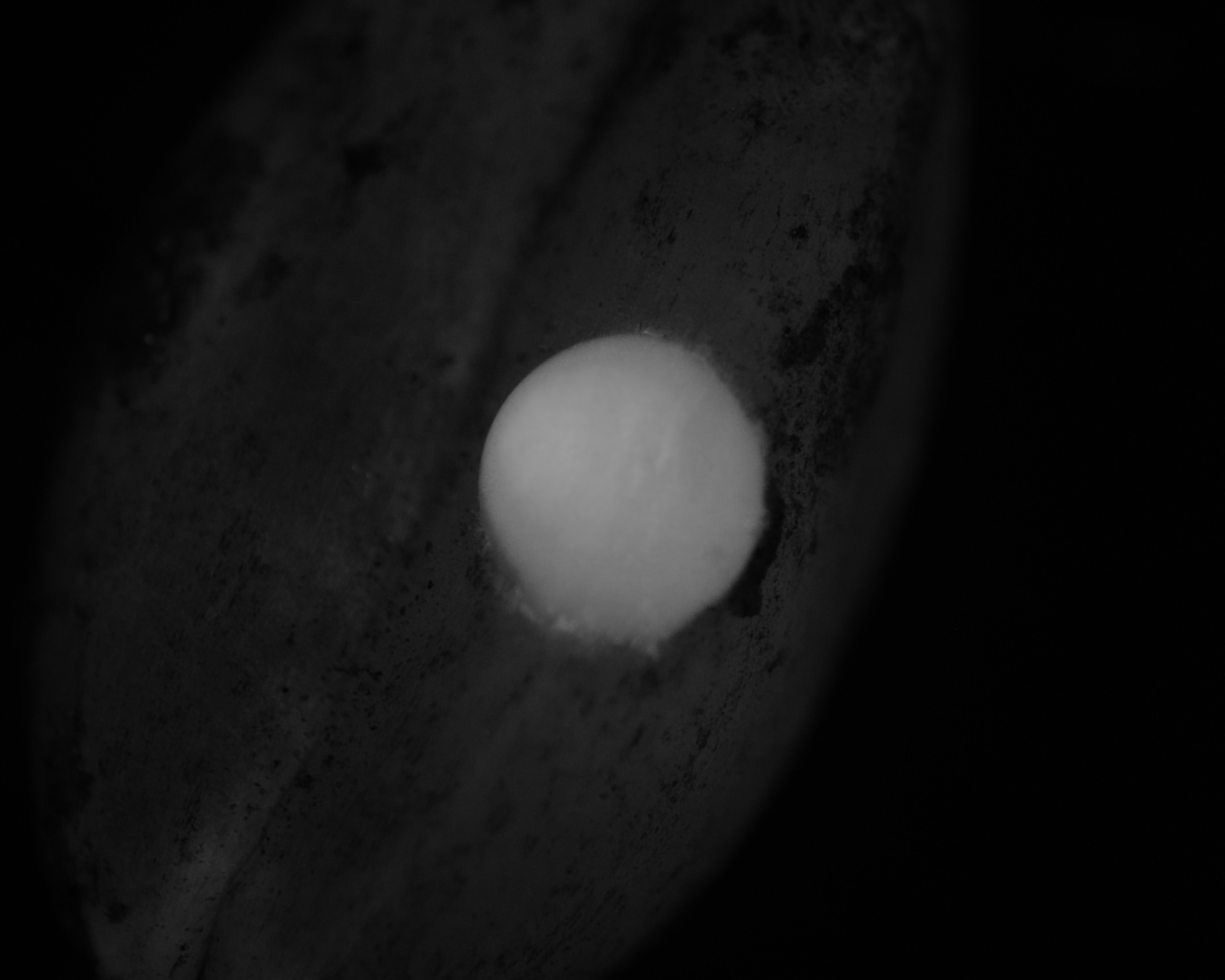

Supplement: S4 File — (ZIP) [file pone.0334274.s004.zip › GezawyDD55 4.tif]

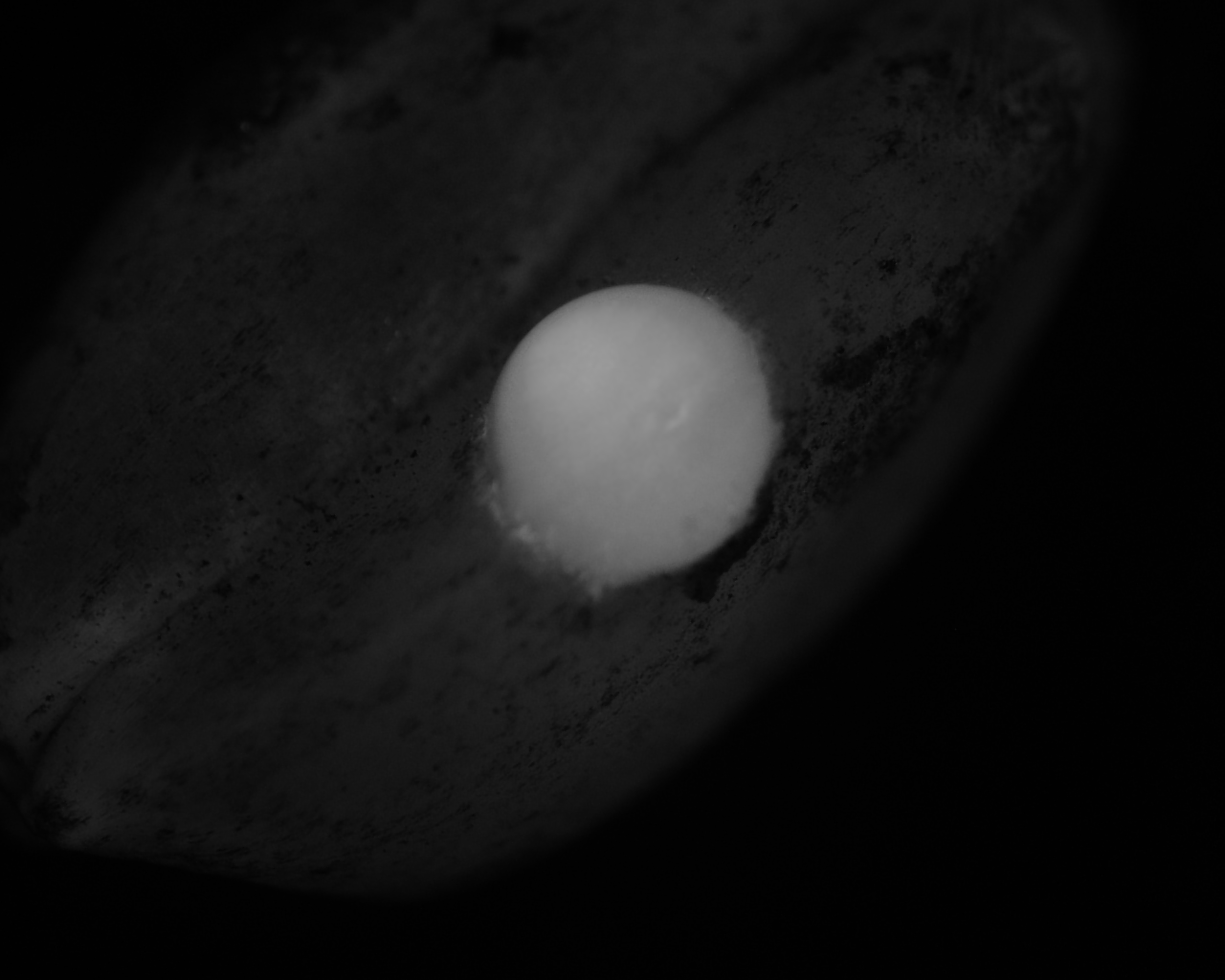

Supplement: S4 File — (ZIP) [file pone.0334274.s004.zip › GezawyDD55 5.tif]

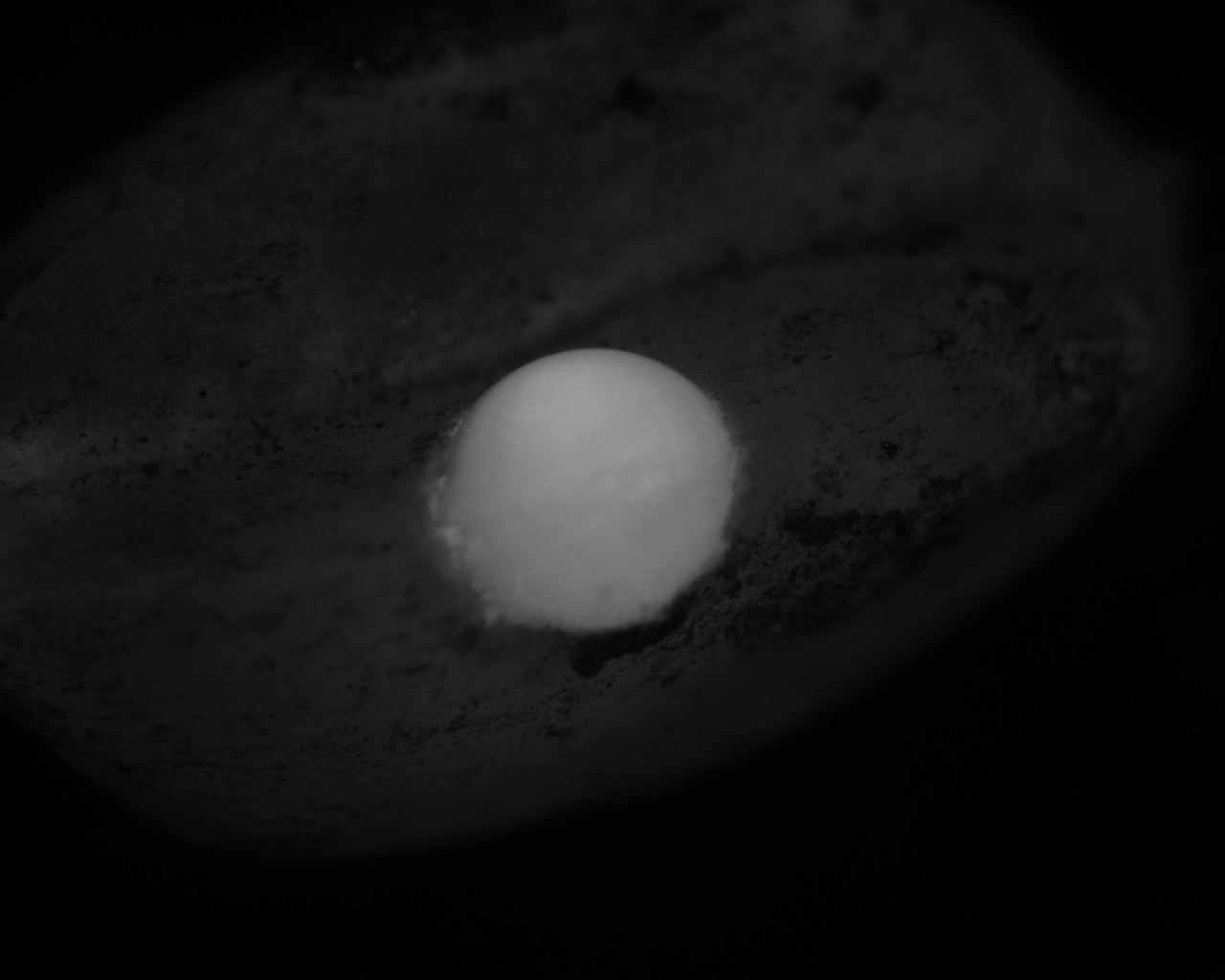

Supplement: S4 File — (ZIP) [file pone.0334274.s004.zip › GezawyDD55 6.tif]

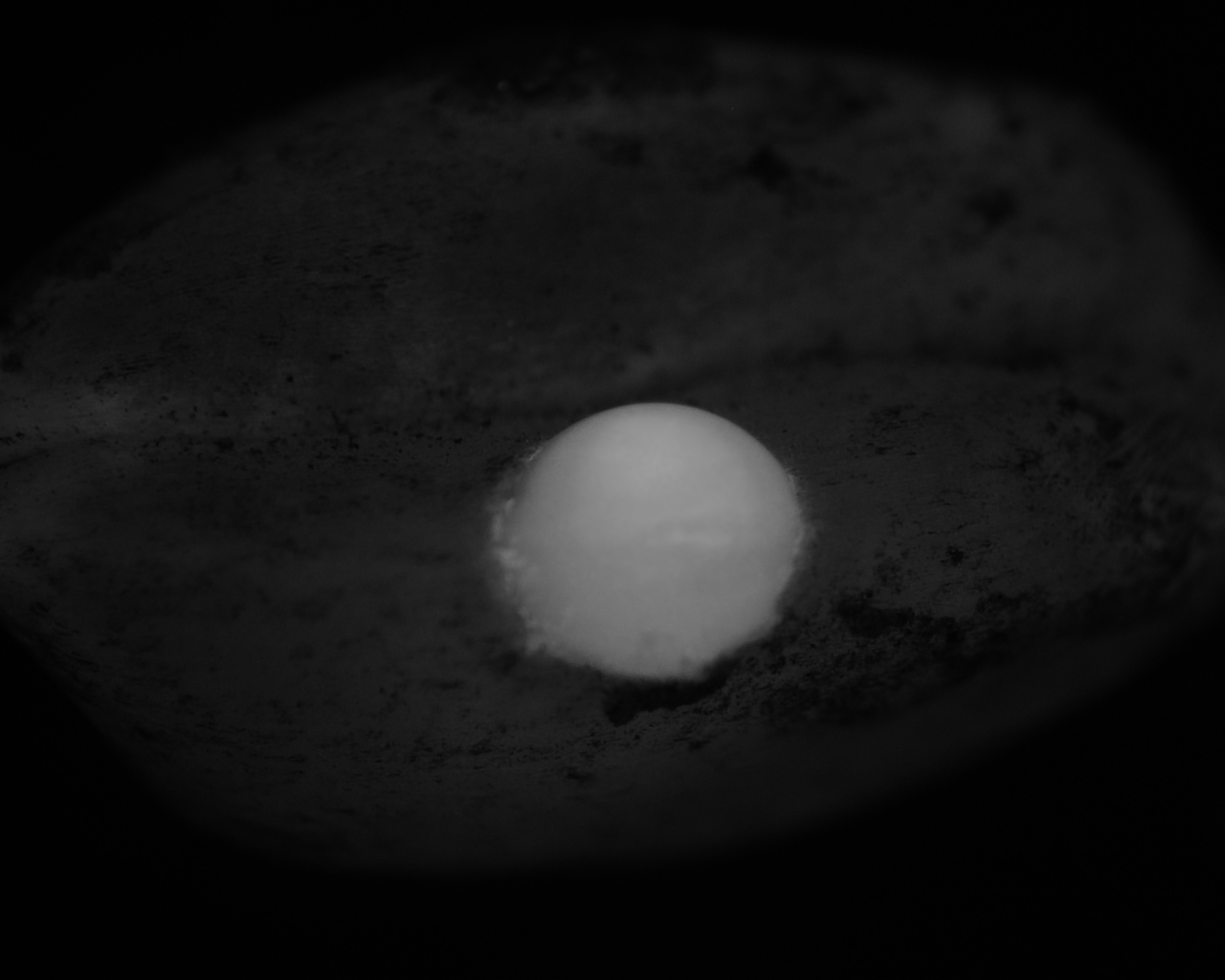

Supplement: S4 File — (ZIP) [file pone.0334274.s004.zip › GezawyDD55 7.tif]
